# Crystallization of Bulk Polymers With Chain Folding: Theory of Growth of Lamellar Spherulites

**DOI:** 10.6028/jres.065A.035

**Published:** 1961-08-01

**Authors:** John D. Hoffman, John I. Lauritzen

## Abstract

A systematic study of the problem of spherulitic growth in linear polymers in bulk has been carried out. A calculation of the radial growth of polymer spherulites is given for four models. These concern growth where the surface nuclei that control the rate are (1) bundlelike and coherent, (2) chain folded and coherent, (3) chain folded and noncoherent, and (4) bundlelike and noncoherent. The required modifications of nucleation theory are given. Then the radial growth rate laws are derived for each model, and the type of “spherulite” that would be formed discussed.

The model with chain folded and coherent growth nuclei leads to a typical lamellar spherulite. The properties of the individual chain folded lamellae that form the spherulite are predicted, including the change of step height with growth temperature, melting behavior, and the behavior on recrystallization. (Chain folded lamellae may also occur in specimens that are not obviously spherulitic.) Under certain conditions, the noncoherent model with chain folds can lead to a modified lamellar spherulite. None of the bundlelike models will lead to a typical lamellar spherulite, though a spherical microcrystalline object might be formed. It is concluded that lamellar spherulites consist largely of chain folded structures.

The factors that could cause chain folded crystals to appear in profusion in bulk polymers are discussed. The case of homogeneous initiation is considered first. Homogeneous initiation of chain folded nuclei in bulk will prevail if the end surface free energy of the bundlelike nucleus exceeds that of the folded. It is shown that the end surface free energy of the bundlelike nucleus, as calculated with a density gradient model, will be larger than had been supposed previously. It is therefore considered to be theoretically possible that the end surface free energy of the bundlelike nucleus may in some cases exceed that of the folded nucleus. Attention is given to the possibility that folded structures appear in large numbers because cumulative strain or large chain ends prevent the growth of bundlelike nuclei to large size, even when the latter type of nucleus is energetically favored when small. Heterogeneous initiation of folded structures is then considered.

Other topics mentioned include: (1) Conditions that might lead to nonlamellar or nonspherulitic crystallization in bulk, (2) the origin of the twist that is frequently exhibited by the lamellae in spherulites, (3) the transitions that may sometimes occur in the radial growth rate law, and (4) interlamellar links.

## ★1. Introduction^1^

In recent years there has been much interest in the growth of spherulites in bulk polymers. These objects are the principal site of the crystallization in a number of highly crystallizable linear polymers, at least under certain conditions. Further, the mechanical, optical, and dielectric properties of such polymers are known to be affected by the presence of spherulitic crystallinity. Hence the rate of spherulitic growth, and the type of crystals existing in the spherulites, are of prime importance in connection with any attempt to understand the physical properties of these systems. Except where specifically noted otherwise, this paper is confined to crystallization from the unoriented melt. Attention is directed mainly to polymers that may be represented as systems of flexible linear chains which can in time achieve a high degree of crystallinity.

In introducing the subject of the nature of the crystals in polymer spherulites, it is essential to first mention the important studies of Keller and coworkers [1, 2]^2^ on the nature of the platelike single crystals of linear polymers that may be deposited from supercooled dilute solutions. Polymer crystals formed in this manner consist of regularly shaped platelets that have a thinness or “step height” of roughly 50 to 250 A, depending on the degree of supercooling. (The step height is larger the lower the supercooling.) By electron diffraction experiments, Keller demonstrated that the long axes of the polymer molecules in such crystals are approximately perpendicular to the large and flat upper and lower surfaces. Since the polymer molecules were known to be much longer than the step height, the startling but nevertheless definite conclusion was reached that the upper and lower surfaces consist of *chain folds.* Stacks of thin platelets resembling a terraced pyramid are frequently formed in dilute solution preparations, often around what appears to be a spiral dislocation. In such cases, each terrace step corresponds to the thickness or step height of a single crystal. A theory describing the formation of single polymer crystals from dilute solution has been given by Lauritzen and Hoffman [3].

Spherulites in bulk polymers grow outward from a nucleation center that is frequently of a heterogeneous character. The radial growth of a spherulite is commonly the result of the formation of stacks of bladelike lamellae that grow outward from the nucleation center. As shown in electron micrographs of surface replicas of spherulites, these lamellae possess a thickness or “step height,” corresponding to the thin dimensions of the blade, that is commonly between 50 and 250 A. The resemblance between the system of steps seen in a bulk polymer and the steps seen in the stacks of chain folded platelets in a dilute solution preparation of the same polymer is most striking. It is well known from optical studies that the polymer chains in the crystalline matter in spherulites are approximately normal to the spherulite radius. Since the lamellae lie mostly parallel to the radius of the spherulite, it is therefore reasonable to assume that the polymer chains are more or less normal to the large flat surfaces of the lamellae. (Evidence interpretable as proof that the chain axes are nearly normal to the flat lamellar surfaces in polyethylene has been obtained by microcamera X-ray diffraction studies by Fujiwara [42]. Other investigators [4] have shown by optical methods that a similar condition probably applies in certain other cases.) The lamellae often twist as they grow outward, so that a given sector of a spherulite somewhat resembles a stack of propeller blades (twisted lamellae) radiating from a central point. We refer to an object fitting this general description as a *lamellar spherulite*.^3^

In view of the above, it is certainly reasonable to give strong consideration to the possibility that a lamellar spherulite formed in bulk consists of chain folded crystals of the same general type known to arise in dilute solution, and to consider spherulite growth mechanisms based on the chain folded pattern. At the same time, one must attempt to construct a lamellar spherulite on the customary bundlelike pattern. By carrying out calculations on both models, it is possible to arrive at certain conclusions concerning the existence of chain folded crystals in bulk polymers. In the course of such an attack on the problem, it is natural to see if any of the models are capable of predicting the existence of a nonlamellar spherulite.

While no attempt will be made in this paper to effect a detailed comparison of theory and experiment, or to give a complete survey of the experimental situation with respect to the nature of spherulitic crystallization in bulk polymers, it is of interest to mention some of the studies on which the above remarks on spherulitic structure in bulk are based. Much of what is known about lamellae in spherulites has been learned from studying electron micrographs. An outstanding example of this approach is to be found in the recent work of Geil [5] on polyoxymethylene, where clear evidence of the lamellar structure of bulk crystallized material is presented. Geil, Symons, and Scott [6] have shown that chain folded crystals with a rather similar step height are formed from dilute solution by polyoxymethylene; the resemblance between the terraces formed by stacks of chain folded crystals in dilute solution and the terraces seen in material crystallized in bulk is especially striking in this polymer. Eppe, Fischer, and Stuart have presented clear evidence of lamellar structure in bulk polychlorotrifluoroethylene [40]. This and other examples that could be cited render it clear that lamellar structures are common in linear polymers crystallized in bulk. Information on the orientation of the lamellae and polymer chains in spherulites, and the twist of the lamellae, has been obtained by optical microscopy together with a detailed theory of the extinction patterns of such objects due to Keller [7], Keith and Padden [8, 9], and Price [10]. The beautiful rings seen in spherulites in a polarizing microscope are a result of the twist of the lamellae.

The body of the paper begins with a discussion of homogeneous initiation of bundle and loop type structures in bulk. It is concluded that if homogeneous nucleation is the cause of the prevalence of folded structures in bulk, the end surface free energy of the bundlelike nucleus, *σ_e_*, must exceed that of the end surface free energy of the nucleus with chain folds, ***σ****_e_.* (A simple bundlelike nucleus model that explicitly involves the density gradient at the bundle end is used to bring out certain factors that may contribute to *σ_e_.*) Then the possibility that large chain ends or strain may tend to subdue growth of bundlelike nuclei to large size is considered. Finally, since it is by no means certain that spherulites are generally of homogeneous origin, heterogeneous initiation of chain folded lamellar structures is discussed.

With this background, attention is then directed toward the problem of calculating *the isothermal rate of radial growth of spherulites as a function of temperature* in supercooled bulk polymers. This property has been singled out for special emphasis because it can often be determined experimentally as a function of temperature, and does not depend on whether the spherulites are of heterogeneous or homogeneous origin.

Radial growth where the rate determining step is the formation of a two-dimensional *coherent* surface nucleus is treated first. (By the term “coherent” we mean to imply that the crystal structure and molecular orientation in the surface nucleus and in its polymer substrate are essentially the same.) Both *bundlelike* coherent surface nuclei and coherent surface nuclei with *chain folds* are considered.

Treatments of radial spherulitic growth by two-dimensional coherent surface nucleation have been given by Burnett and McDevit [11], Kahle and Stuart [12], Takayanagi [13], and Hirai [14]. We must now indicate why we shall add yet another.

First, it is considered desirable to give a detailed analysis of spherulitic growth for coherent nuclei with chain folds. Second, it is instructive to reexamine the theory of the radial growth rate for coherent bundlelike nuclei. It is evident that some problems connected with this model have not been emphasized previously, nor its full range of behavior elucidated. Third, it is necessary to treat the general problem of growth by monomolecular accretion, taking into account the fact that the activated state is in some instances reached in one step. This leads to an important revision of the nucleation and growth rate expressions, especially in the chain fold case. Fourth, it is important to consider the role of chain ends on the formation of polymer crystals. Finally, it is considered to be of special interest to examine closely the connection between a given model for the growth rate, and the physical structure and orientation of the crystalline bodies that this model actually implies in a spherulite. It will emerge that some of the models frequently cited in the literature and used for the analysis of radial growth rate data on spherulites will not lead to typical spherulitic structures.

It will be demonstrated that radial growth through the agency of coherent nucleation with chain folds can lead to a three-dimensional object recognizable as a lamellar spherulite. The existence of the lamellae, the orientation of these lamellae and the molecules in them with respect to the spherulite radius, the dependence of the step height on growth temperature, recrystallization behavior, and the twist exhibited by the lamellae, can be predicted. It is adduced that the coherent bundlelike model cannot lead to a lamellar spherulite.

The theory given here for the rate of radial spherulitic growth in bulk as controlled by coherent surface nuclei with chain folds is based mostly on an analysis by Lauritzen and Hoffman [3] on the appearance of polymer crystals with chain folds from dilute solution. Price [15] has independently treated some aspects of this problem.

The problem of calculating the rate of radial growth of a spherulite where the rate determining step is the formation of a three-dimensional *noncoherent* surface nucleus is treated next. (By the term “noncoherent” we mean to indicate that the orientation of the molecules in the surface nucleus is different from that in the polymer crystal to which it is attached, so that a definite interface exists between the substrate polymer crystal and the surface nucleus.) As before, *bundlelike* noncoherent surface nuclei, and noncoherent surface nuclei with *chain folds*, are treated. The concept of noncoherent surface nucleation in spherulites treated here is based largely on an interesting suggestion due to Price [16]. The radial growth rate laws are given, and the nature of the resultant physical structures is predicted.

The results obtained for the bundlelike noncoherent growth model resemble in general form those given previously by Flory and McIntyre [17], who proposed that the free energy of formation of the three-dimensional bundlelike surface nucleus is in some manner lowered in the vicinity of the growing boundary compared to the free energy of formation of the corresponding three-dimensional homogeneous nucleus. The present treatment is more explicit concerning the possible cause of the lowering of the free energy of formation of the surface nucleus. The noncoherent bundlelike model might possibly lead to a spherical and microcrystalline but non- lamellar object. The noncoherent chain folded model leads to a somewhat modified lamellar spherulite.

In all the calculations of the radial growth rate, an effort has been made not only to derive the rate laws near and somewhat below the melting point, but also to determine the type of behavior that might obtain at strong supercooling. Rather abrupt changes in the radial growth rate, and even the mode of crystallization, may occur with sufficient supercooling.

Toward the end of the paper, the radial growth rate equations are summarized in tabular form, and a discussion presented on various aspects of the initiation and growth of spherulites, and lamellar and nonlamellar crystallization in polymers.

## ★2. Rate of Homogeneous Initiation in Bulk for Bundlelike and Chain Folded Nuclei

### 2.1. Homogeneous Nucleation Theory

Turnbull and Fisher [18] give for the steady state rate of homogeneous nucleation in a condensed system the expression

$$I=K\frac{NkT}{h}\exp\;\left(\frac{\Delta S*}{k}\right)\;\exp\;\left(-\frac{\Delta H*}{kT}\right)\;\exp\;\left(-\frac{\Delta \phi *}{kT}\right)\;.$$ (1)

Here *T* is the absolute temperature, *h* Planck’s constant, *k* Boltzmann’s constant, ΔH* the heat of activation of the elementary jump rate process at the supercooled liquid—nucleus interface, ΔS* the corresponding entropy of activation, and Δϕ* the free energy of formation of a nucleus of critical size, i.e., a nucleus at the saddle point in the appropriate free energy surface. The constant *K* has a numerical value within several orders of magnitude of unity for most nucleation problems of interest, and may be ignored for the present.^4^ Equation (1) was derived on the basis that the nucleus contains a large number of segments or atoms, and is built up in a stepwise manner. The net nucleation rate was obtained by summation over all forward and backward reactions. The pre-exponential factor (*NkT*/*h*) exp (ΔS*/*k*) nuclei sec^−1^ mole^−1^ may be converted to $I_{0}=(NkT/hm_{0}{\bar{V}}_{a})$ exp (ΔS*/*k*) nuclei sec^−1^ cm^−3^, where *m*_0_ is the molecular weight of the length of polymer segment that enters the nucleus in an elementary process, and ${\bar{V}}_{a}$ the specific volume of the supercooled liquid.

The main problem is the calculation of Δϕ* in terms of the surface free energies and other parameters associated with the model under consideration.

### 2.2. Primary Bundlelike Nuclei: The End Surface Free Energy Problem

**Figure 1:**
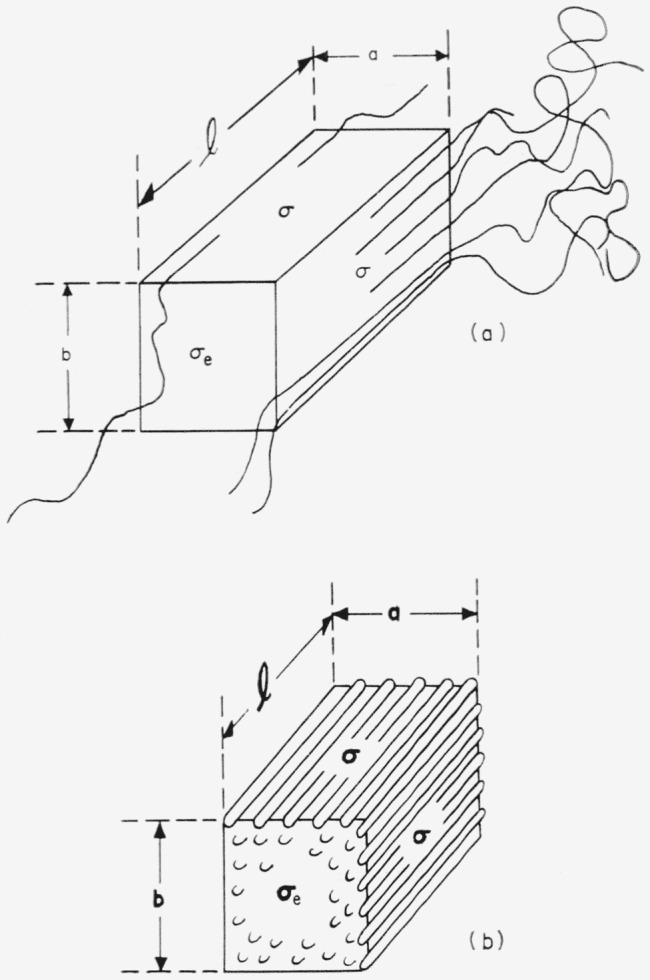
Bundlelike and chain folded primary nuclei. (a) *Bundlelike nucleus:* ordinary symbols are used to denote the dimensions *a, b*, and *l*, and the lateral and end surface free energies *σ* and *σ_e_.* A density gradient of considerable extent in the *l* direction will exist at the end surface. (b) *Nucleus with chain folds:* bold face symbols are used for the dimensions **a**, **b**, and **l**, and the lateral and end surface free energies ***σ*** and ***σ****_e_*. Well defined surfaces exist on all faces.

We employ a bundlelike primary nucleus that is a rectangular parallelepiped as shown in figure 1a. The quantity *σ* is the lateral surface free energy, and *σ_e_* is the end surface free energy. The surface free energies are defined as the work that is needed to isothermally form 1 cm^2^ of the appropriate type of surface from the required number of segments in the normal (interior) crystalline phase.

The rectangular parallelepiped model is not proposed with the intention of conveying the meaning that the cross section of a nucleus or crystal is necessarily rectangular. The cross section could have other shapes, e.g., a parallelogram, hexagon, or other polygon. The lateral faces of the crystal will correspond to some single preferred crystallographic plane, and the lateral surface free energy *σ* will correspond to the work required to form 1 cm^2^ of this surface. The particular geometrical model chosen is just the simplest that is sufficiently illustrative of the phenomena we wish to discuss. The slight modifications necessary to deal with other cross-sectional shapes have been outlined elsewhere [3].

#### Lateral Surface Free Energy (Bundles)

The lateral surface free energy *σ* refers to a definite and well defined surface, and a reasonably good estimate of its numerical value can be obtained. We should expect *σ* to be fairly close to that for a typical molecular crystal of approximately the same chemical composition as the polymer, since the molecules on the lateral faces are not “connected” through covalent bonds to the surrounding supercooled liquid. Rough estimates of *σ* may be surmised from the results of Thomas and Stavely [19] on homogeneous crystallization in fogs of supercooled droplets of simple nonchain organic compounds. From this work we would anticipate that the lateral surface free energy *σ* would frequently be in the range of 5 to 25 erg cm^−2^.

It is useful to indicate a method of estimating the lateral surface free energy for a polymer that should generally give a value that is more accurate than would be guessed simply by perusing the results on nonchain type molecular crystals quoted by Thomas and Stavely. Several authors [19, 20] have suggested that for a specific class of compounds the ratio of the work required to form a certain amount of surface phase to the heat of fusion of the same amount of bulk phase is approximately a constant. This ratio may be written as

$$\frac{\sigma }{(\Delta h_{f})d}=\alpha ,$$ (2)

where (Δ*h_f_*) is the heat of fusion in erg cm^−3^, *d* the lattice spacing in cm, and *σ* the surface free energy in erg cm^−2^. The constant *α* is about 0.5 for many metals [20], and about 0.3 for simple (non-chainlike) molecular crystals [19]. Using the values of *σ* obtained from carefully conducted homogeneous nucleation experiments with pure chain hydrocarbons dispersed in water recently quoted by Turnbull [21]^5^, we estimate that *α* is about 0.1 for the paraffin chain system. This value of *α* should hold quite well for the calculation of the *lateral* surface free energy *σ* of polyethylene (*σ* ~ 10 erg cm^−2^). It is to be expected that a fairly similar value of *α* will hold for the estimation of the lateral surface free energies of other linear polymers. Turnbull’s technique could be used to obtain more precise values of *α* appropriate to other types of chain structure.

#### End Surface Free Energy (Bundles)

The value of *σ_e_* appropriate to the bundlelike nucleus is difficult to assess. No reliable experimental values seem to be available. Flory [22] has treated the configurational contribution to the end surface free energy for a bundlelike crystal as a function of concentration. In this formulation, the end surface free energy at *v*_2_=1 (bulk phase) is proportional to *RT* ln *D*, where *D* is a parameter. No theoretical method of evaluating *D* was given. Also, Flory’s treatment does not deal explicitly with the density gradient region at the bundle ends, where important contributions to *σ_e_* will arise.

Insight into some of the factors that will contribute to the work required to build the end of a bundlelike nucleus with a flat end may be gained by noting the calculations in the appendix, section 10, for a cylindrical bundlelike nucleus with a density gradient at the bundle ends. This simplified treatment shows to the approximations indicated (see eq (A-20) of the appendix) that

$$\sigma_{e}=\frac{l_{d}(\Delta h_{f})\rho_{c}}{6\rho_{l}}+\frac{l_{d}{\left(\Delta \rho \right)}^{2}\rho_{0}\Gamma }{6\rho_{l}^{2}\rho_{c}}.$$ (3)

Here *l_d_* is the length of one of the diffuse bundle ends in cm, (Δ*h_f_*) the heat of fusion in erg cm^−3^, *ρ_c_* and *ρ_l_* the density in g cm^−3^ of the crystal and supercooled liquid, respectively, (Δ*ρ*) *= ρ_c_−ρ_l_, ρ*_o_= (*ρ_c_*+*ρ_l_*)/2, and Γ a constant in erg cm*^−^*^3^ that may reasonably be expected to exceed zero. The magnitude of Γ is related to the height of the maximum that will exist in the free energy somewhere in the bundle ends. This maximum must exist in order to cause phase separation, and may result from either repulsion or abnormal separation of the segments in the partly disordered region of the bundle ends. The derivation of eq (3) is valid only when the cross section of the nucleus contains a fairly large number of polymer molecules.

Equation (3) with Γ=0 may be used to obtain a reasonable lower limit on the value of *σ_e_* for a bundlelike nucleus with a flat end, which we call *σ_e_*_(min)_. The minimum value of *σ_e_* implied by eq (3) is surprisingly large. For example, with (Δ*h_f_*)=3×10^9^ erg cm^−3^, *ρ_c_*/*ρ_l_*=1.15, parameters that apply approximately to polyethylene,^6^ when taken together with the assumption that *l_d_*=10×10^−8^ cm = 10 A, lead to *σ_e_*_(min)_≅60 erg cm^−2^. Reflected in this result is the fact that a fairly large amount of work will be required to construct an interface between two phases that are connected together by chains containing covalent bonds.

The true value of *σ_e_* for polyethylene is probably in excess of this, since Γ may be greater than zero. Also, certain cumulative strain contributions are neglected in the derivation of eq (3), and these would further increase *σ_e_.* From the above we draw the conclusion that it is not necessary to consider the effect of small *σ_e_* values, i.e., those substantially smaller than the lateral surface free energy *σ*, in calculating the properties of bundlelike nuclei.^7^ It is entirely possible that *σ_e_* for bundlelike nuclei may be at least several hundred erg cm^−2^ in some instances.

An analysis of the particular model used to arrive at eq (3) does not suggest a marked dependence of *σ_e_* on temperature. Nevertheless, even the simplified treatment outlined in the appendix indicates that, under certain circumstances, *σ_e_* could depend on temperature to a noticeable extent. In view of the above, *we must emphasize that the calculations to follow with σ_e_ treated as a constant are approximate*. Nevertheless, they are believed to be sufficient for the purpose of dealing with the question of how nuclei with chain folds might come to prevail over bundlelike nuclei in bulk. The symbol *σ_e_* in the expressions to be derived for the bundlelike nucleus may be thought of as representing an effective value containing contributions analogous to those shown in eq (3).

### 2.3. Rate of Homogeneous Nucleation for Bundlelike Nuclei

#### Region A (steady state nucleation)

In the temperature region near and somewhat below the melting point, which we designate region A, the primary nuclei are much larger than the unit cell, and the free energy of formation of a bundlelike crystal or embryo of the type shown in figure 1a may be written

$$\Delta \phi =2ab\sigma_{e}+2al\sigma +2bl\sigma -abl(\Delta f),$$ (4)

where *a*, *b*, and *l* are treated as variables. (Δ*f*) is the bulk free energy of fusion per unit volume of crystal. Both *σ* and *σ_e_* are regarded as constants. The problem is to calculate Δϕ* by finding the saddle point in the free energy surface described by eq (4).

The saddle point in the free energy surface described by eq (4) is found by setting ∂Δ*ϕ*/∂*a*)*_bl_*, ∂Δ*ϕ*/∂*b*)*al* and ∂Δ*ϕ*/∂*l*)*_ab_* equal to zero to get a*= 4*σ*/(Δ*f*), b*=4*σ*/(Δ*f*), and l*=4*σ_e_*/Δ*f*. As plotted on the orthogonal coordinates Δ*ϕ*, *l*, (*ab*)^1/2^, the saddle point is at l*=4*σ_e_*/Δ*f*, and (a*b*)^1/2^=4*σ*/(Δ*f*). Inserting these values into eq (4) the value of the free energy of formation at the saddle point is found to be

$$\Delta \phi *=\frac{32\sigma^{2}\sigma_{e}}{{\left(\Delta f\right)}^{2}},$$ (5)

which may be compared with the value 8*πσ*^2^*σ_e_*/(Δ*f*)^2^ for a bundlelike nucleus with a circular cross section. Then with eq (1), the steady-state nucleation rate is

$$\begin{array}{c} I_{A}=I_{0}\;\exp\;\left(-\frac{\Delta H*}{kT}\right)\;\exp\;\left(-\frac{32\sigma^{2}\sigma_{e}T_{m}^{4}}{T^{2}{\left(\Delta h_{f}\right)}^{2}{\left(\Delta T\right)}^{2}kT}\right),\\ \left[{\left(\Delta T\right)}^{-2}\text{law}\right] \end{array}$$ (6)

where we have set

$$\Delta f=\left(\frac{\Delta h_{f}\;(\Delta T)}{T_{m}}\right)\;\left(\frac{T}{T_{m}}\right).$$ (7)

Here Δ*h_f_* is the heat of fusion per unit volume of crystal at the equilibrium melting temperature, *T_m_*, and (Δ*T*) the degree of supercooling, *T_m_*–*T*, where *T* is the crystallization temperature. It has been shown in a previous study [23] that eq (7) is a good approximation in a glass-forming system.^8^

#### Region B (nonsteady state nucleation)

Observe from the foregoing that a*=b*=4*σ*/(Δ*f*). From this expression and eq (7) it is clear that at some high degree of supercooling (large Δ*T*), the *a* and *b* dimensions of a nucleus of critical size will approach their minimal values, *a*_min_ and *b*_min_. This will occur at a temperature *T_c_* corresponding to a degree of supercooling of approximately

$$\Delta T_{c}≅4\sigma T_{m}/(\Delta h_{f})a_{\min}.$$ (8)

As a rough approximation the product (*a*_min_*b*_min_) may be taken as the area corresponding to a nucleus with a cross section containing roughly 5 to 7 polymer segments, i.e., a body with at least one central molecule in an ordered environment. This is the smallest object that may be considered as a typical nucleus. (Note that *a*_min_ will be somewhat larger than the corresponding dimensions of the unit cell.)

Because Δ*T_c_* depends on the well-defined lateral surface free energy *σ*, for which numerical values can be estimated with reasonable accuracy, a fairly reliable conception of its magnitude can be obtained. Taking *σ*=5 erg cm^−2^, *a*_min_=*b*_min_=10×10^−8^ cm, *T_m_*=400 °K, and (Δ*h_f_*) = 10^9^ erg cm^−3^, Δ*T_c_* calculated from eq (12) comes to 80 °C. Δ*T_c_* should rarely be less than 30 or 40 °C, and in many cases it may be so large that it falls near or below the glass transition, which would render it inherently unobservable.

It is clear that the nucleation rate will change its character near and below *T_c_*. Attention is now directed to the interesting question of the nature of the nucleation process in the temperature region near and below *T_c_*, region B.

At and below *T_c_*, the free energy of formation may be written

$$\Delta \phi =2a_{\min}b_{\min}\sigma_{e}+l[2a_{\min}\sigma +2b_{\min}\sigma -a_{\min}b_{\min}(\Delta f)].$$ (9)

The coefficient of *l* is zero at *T_c_*, but becomes negative at lower temperatures. Thus, it is seen that the nucleus is formed by “increasing” its length *l* to its minimum possible dimension, which we call *l*_min_. Hence, in effect, we are calculating the steady-state nucleation rate for a nucleus of fixed dimensions *a*_min_, *b*_min_*, l*_min_. The modification of classical continuum free energy surface theory necessary to deal with this type of problem is mentioned in section 3.1. The appropriate free energy of formation is given by eq (9) with *l*=*l*_min_ The steady state nucleation rate in region B is

$$I_{B}=I_{0}\;\exp\;\left[-\frac{\Delta H*+\Delta H**}{kT}\right]\times \exp\;\left[\frac{a_{\min}b_{\min}l_{\min}(\Delta h_{f})(\Delta T)}{T_{m}k}\right],$$ (10)

[(Δ*T*)^+1^ law; will not be directly observable]

where the constant term 2*b*_min_(*a*_min_*σ_e_*+*l*_min_*σ*) is denoted as ΔH**. (Ordinarily, ΔH** will not exceed several kcal mole^−1^.) This (Δ*T*)^+1^ steady state nucleation rate expression appears to be new in nucleation and growth theory. It must immediately be pointed out, however, that it is very unlikely that such a rate law would be observed over any substantial range of temperature because of the effect of nonsteady-state nucleation near and below *T_c_*. In the case of such small nuclei forming by steady state nucleation in a strongly supercooled polymer, the additional effect of pre-existing embryos of minimal size must be taken into account.^9^

At any temperature *T*_1_ above the melting point of the polymer, the free energy of formation of an embryo always increases as its size increases. This is in contrast to the case of embryos in the supercooled liquid state, where the free energy of formation goes through a maximum at a saddle point so that embryos can become nuclei, and eventually stable crystallites. Nevertheless, numerous small embryos will exist in the normal liquid above *T_m_*, and the population of such embryos can be estimated by straightforward methods. (In the expression for Δ*ϕ*, Δ*T* simply changes sign above *T_m_*.) Now when a polymer specimen is rapidly cooled from a temperature *T*_1_ that is above *T_m_* to a temperature *T*_2_ in the strongly supercooled state, a number of these pre-existing embryos will be found to be of the critical size relevant to *T*_2_. The number of such pre-existing embryos that correspond to nuclei of critical size will be negligible if the stable nuclei arc large, as will be the case at low or moderate supercooling, but near and below *T_c_*, where the nuclei are of the minimal dimensions *a*_min_, *b*_min_, l_min_, the number of pre-existing embryos that correspond to nuclei of critical size will be high. Thus, near and below *T_c_*, this transport of bundlelike embryos from the normal melt to the strongly supercooled state will greatly *increase* the rate of injection of nuclei above that predicted by eq (10), or eq (6) as extrapolated into region B. Calculations show that this effect might become so pronounced somewhat below *T_c_* as to cause a very rapid and fine-grained crystallization to occur that might aptly be described as a “nucleative collapse” of the supercooled liquid state. (This effect may deter glass formation as noted in section 8.2.)

At sufficiently low temperatures, the rate of injection will eventually fall because of the increasing importance of the interfacial jump rate term, exp (−ΔH*/*kT*). Depending on the values of ΔH* and the various surface free energies involved, this could happen in regions A or B.

#### Summary

**Figure 2:**
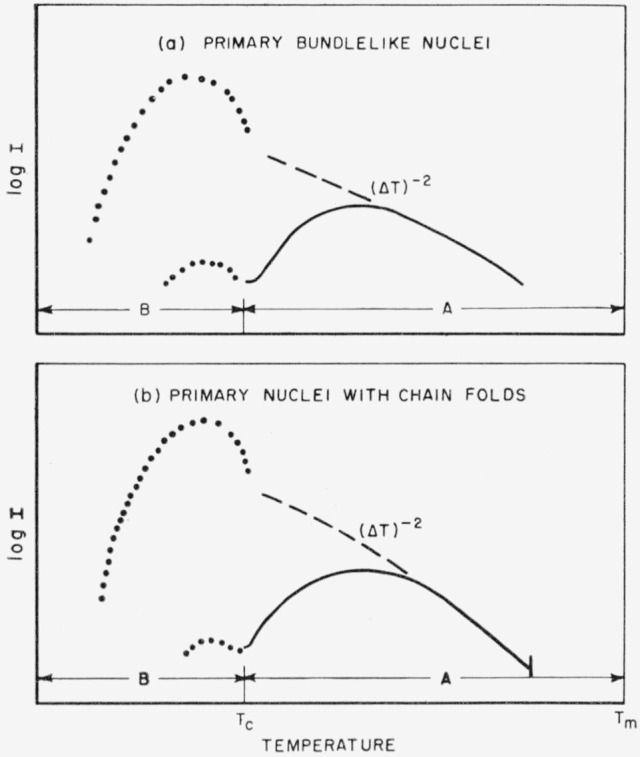
Rate of injection of homogeneous nuclei as a function of temperature. *I* is the homogeneous nucleation rate in nuclei per second per unit volume. — — — — behavior of steady state value of log *I* if effect of jump rate is small; ▬▬customary behavior where jump rate lowers log *I;…..* shows nucleation in excess of steady state value of log *I* in region B resulting from transport of nuclei of minimal size from above *T_m_.*

The overall picture of the homogeneous nucleation rate for bundlelike nuclei is shown in figure 2a. The interfacial jump rate term, exp (−ΔH*/*kT*), with its positive temperature coefficient, will commonly overcome the strongly negative temperature coefficient of the nucleation term, exp $\exp\;[-32\sigma^{2}\sigma_{e}T_{m}^{4}/T^{2}{\left(\Delta h_{f}\right)}^{2}{\left(\Delta T\right)}^{2}kT]$, and cause a maximum to appear in the steady state nucleation rate in region A (see solid line). If the nucleation rate is still observable at all at and below *T_c_*, the excess nucleation rate characteristic of nonsteady state nucleation in region B may be seen.

### 2.4. Primary Nuclei With Chain Folds

Quantities that are closely related or specific to *chain folds* are denoted by *bold face* symbols. Note especially that

$$\begin{array}{r} \text{folded nuclei}\\ \text{or crystals} \end{array}\{\begin{array}{l} \sigma_{e}=end\;surface\;free\;energy\\ \sigma \;=lateral\;surface\;free\;energy \end{array}$$

The corresponding quantities for bundlelike systems are

$$\begin{array}{r} \text{bundlelike}\\ \text{nuclei or}\\ \text{crystals} \end{array}\}\begin{array}{l} \sigma_{e}=end\;surface\;free\;energy\\ \sigma \;=lateral\;surface\;free\;energy \end{array}$$

The specific type of folded nucleus to be discussed is shown in figure 1b.

#### Lateral Surface Free Energy (Folded Crystals)

The lateral surface free energy ***σ*** for the folded nucleus refers to an abrupt phase boundary, since no polymer molecules pass through this surface and connect the supercooled liquid and crystalline phases. The quantity ***σ*** is thus similar in general character to the quantity *σ* for the bundlelike system, i.e., *σ*≅***σ***, and eq (2) applies to its estimation. Thus, ***σ*** will usually fall between 5 and 25 erg cm^−2^.

#### End Surface Free Energy (Folded Crystals)

The end of the chain folded nucleus, unlike the end of the bundlelike nucleus, has a well defined phase boundary. Hence, *the folded nucleus has abrupt phase boundaries on all its faces.* As a consequence, the treatment of chain folded nuclei can be approached with more certainty than can bundlelike nuclei under like circumstances. Both ***σ*** and ***σ****_e_* may to a good approximation be assumed to be independent of temperature and other variables.

The value of the surface free energy ***σ****_e_* for a folded nucleus is related to the work required to form a fold [3]:

$$\sigma_{e}=\sigma_{e0}+q/2A_{0}.$$ (11)

Here **q** is the work required to form a fold, and *A*_0_ is the area of the cross section of the polymer molecule. An important contribution to **q** will arise from the internal rotational potential of atoms or groups of the loop itself, i.e., from the stiffness of the polymer chain. Then values of **q** in the range of roughly 1 to 10 kcal per mole of loops are to be expected. For molecules with the cross-sectional area ordinarily encountered, say 20×10^−16^ cm^2^, this means that **q**/2*A*_0_ might be expected to run from roughly 15 to 150 erg cm^−2^. (1 kcal/mole of folds=6.95×10^−14^ ergs/fold.) The quantity ***σ****_e_*_0_ is the contribution of ***σ****_e_* due to factors other than folding, and is probably not in excess of ***σ***. Thus we might expect ***σ****_e_* to be somewhere from 15 to 40 erg cm^−2^ to roughly 150 to 175 erg cm^−2^ for chains of normal flexibility and cross section. It is to be expected that ***σ****_e_* will generally be larger than ***σ*** in any specific case.

### 2.5. Rate of Homogeneous Nucleation With Chain Folds

#### Region **A**

By simply replacing *σ_e_* and *σ* in eq (4) with ***σ****_e_* and ***σ***, and proceeding as before, we get

$$\begin{array}{c} I_{A}=I_{0}\;\exp\;\left(-\frac{\Delta H*}{kT}\right)\;\exp\;\left(-\frac{32\sigma^{2}\sigma_{e}T_{m}^{4}}{T^{2}{\left(\Delta h_{f}\right)}^{2}{\left(\Delta T\right)}^{2}kT}\right)\\ \left[{\left(\Delta T\right)}^{-2}\;\text{law}\right] \end{array}$$ (12)

for the steady state rate of injection of loop type nuclei in bulk.

The auxiliary equation

$$l_{p}^{*}=4\sigma_{e}/(\Delta f)≅4\sigma_{e}T_{m}/(\Delta h_{f})\;(\Delta T)$$ (13)

which defines the length or “step height” of the primary nucleus, is obtained in the derivation of eq (12). A numerical calculation using the estimated values of ***σ****_e_*, and reasonable values of the other parameters in eq (13), reveals that the step height of the primary nucleus should frequently lie between 100 and 500 A for a degree of supercooling of 20° C. (As will be noted subsequently, the step height of chain folded lamellae growing by monomolecular accretion may initially be substantially less than that given by eq (13).) Observe from eq (13) that the step height of the primary nucleus increases as the degree of supercooling decreases.

The derivation of eqs (12) and (13) is based on the idea that the primary nucleus is large, i.e., the critical size is reached after many successive steps. Therefore, the free energy surface may be treated as a continuum. As will be seen in section 5.1, this assumption cannot be used for monomolecular growth with chain folds. For the latter, a different theory of the rate of nucleation and step height will be given.

Important restrictions exist on the path of nucleation on the free energy surface for loop type nuclei that are not evident in the simplified derivation indicated above. In particular, the step height of the primary nucleus, $l_{p}^{*}$, may be regarded as essentially invariant as the nucleus is built up. The theory for the constancy of the step height of loop type primary nuclei has been given in detail elsewhere [3]. The step height of one primary nucleus as compared with another is restricted to a narrow range of values centering about $l_{p}^{*}$ because of the steepness of the sides of the saddle point in the free energy surface, and the fact that the chain folds prevent (or seriously deter) an increase of length after a loop is laid down.^10^ This type of restriction does *not* apply to bundlelike nuclei.

#### Effect of Molecular Weight on Nucleation in Upper Part of Region **A**

The critical volume of the primary folded nucleus, v*, is $a*b*l_{p}^{*}=64\;\sigma^{2}\sigma_{e}/{\left(\Delta f\right)}^{3}≅64\sigma^{2}\sigma_{e}T_{m}^{3}/{\left(\Delta h_{f}\right)}^{3}{\left(\Delta T\right)}^{3}$. For the parameters ***σ***=5 erg cm^−2^, ***σ***_e_=25 erg cm^−2^, *T_m_*=400 °K, and (Δ*h_f_*) =10^9^ erg cm^−3^, this comes to 2.6×10^−16^/(Δ*T*)^3^ cm^3^. At relatively low supercooling, e.g., (Δ*T*) = 10 °C, this would give v*=2.6×10^−19^ cm^3^. If it is now assumed that the average length of the polymer molecules, *l_m_*, is 5000 A, and that the cross-sectional area is 20×10^−16^ cm, the mean volume per molecule is calculated to be 10^−19^ cm^3^ molecule^−1^. In this case, the nucleus would have to be formed from more than one polymer molecule. If the polymer molecule has large chain ends that are excluded from the crystal, it follows that the folded nucleus will occasionally possess a chain that emanates bundle-fashion from the plane of the chain folds. This leads to no serious limitation on the theory of nucleation as presented above. At Δ*T*≥14 °C, the primary nucleus could form from one molecule.

If the chain ends are so large as to be practically completely excluded from the crystal, a serious limitation on the ability of the polymer to form a primary nucleus with chain folds will occur as the step height approaches one half the length of the molecule. For the parameters cited above, $l_{p}^{*}=4000A/(\Delta T)$ and *l_m_*=5000 A. Then when Δ*T*≤1.6 °C, chain folded primary nuclei cannot form if chain ends are excluded from the crystal. For materials with higher ***σ****_e_* values or lower molecular weight, this limitation will appear further below *T_m_.* For example, with ***σ****_e_*=50 erg cm^−2^ and *l_m_* = 2000 A, this limitation would appear at Δ*T*≤8 °C. This means that simple homogeneous nucleation with chain folds will not be possible very near the melting point if chain ends are strictly excluded from the crystal. (The same is of course true for bundlelike nucleation at some low supercooling if chain ends are excluded from the crystals.) For polymers of high molecular weight, this limitation on folding is not apt to be encountered in the region where the rate is commonly observed.

It will be possible to tolerate a certain concentration of small chain ends as defects in a polymer crystal. In this case, the degree of supercooling at which chain folds would have difficulty in forming because of chain end effects would be substantially smaller than indicated above. Also, a number of short chains may be included bundle fashion in the nucleus.

#### Region **B**

The expression given for **I***_A_* will hold from temperatures near *T_m_* on down to considerably lower temperatures. Then there will be an increase in the rate of injection of loop type nuclei at a degree of supercooling Δ*T_c_*=4***σ****T_m_*/(Δ*h_f_*)**a**_min_ resulting from the transport of pre-existing loop type embryos, resembling ∪ shaped kinks, from the melt to the supercooled state. The argument for the existence of this nonsteady state nucleation effect is similar to that given previously for bundlelike nuclei, and need not be repeated here.

Note that the expressions for Δ*T_c_* for the bundlelike nucleus and the loop type nucleus are similar, and involve only the lateral surface free energy. The type of nucleus of minimal dimensions that will actually be deposited near and below *T_c_* will be the one that has the lowest free energy of formation, and this will depend largely on which has the lowest end surface free energy. The same symbol is used to denote the transition temperature for both types of nuclei, since it is generally clear which kind is meant.

#### Summary

A schematic diagram of the rate of homogeneous injection of folded nuclei as a function of temperature is shown in figure 2b. The solid line indicates the manner in which the interfacial jump rate term exp (−ΔH*/*kT*) will generally lower the nucleation rate at some degree of supercooling, causing a maximum to appear in **I_A_.** (This maximum would ordinarily appear between 0.8 to 0.9 *T_m_.*) It is probable that the **A**→**B** transition will frequently be unobservable because the jump rate term lowers the nucleation rate so much that *T_c_* falls near or below the glass transition. The pip mark just below *T_m_* represents the temperature above which the molecular length is too small to accommodate chain folding if large chain ends are excluded from the crystal.

### 2.6. Bundlelike *Versus* Folded Nuclei in Bulk

As will be brought out subsequently, there are substantial reasons for believing that lamellar spherulites formed in bulk are composed of crystals with chain folds, each individual lamella having a thickness corresponding to the step height associated with the chain folds. The intriguing question of how folded structures could nucleate and then propagate in linear polymers crystallized in bulk will now be discussed in the light of what may be said about homogeneous nucleation in the bundlelike and folded patterns, the possibility that heterogeneous nucleation may be involved in the initiation act, and the fact that under certain conditions chain ends or strain may interfere with the formation of large bundle like objects, even under conditions where small bundlelike nuclei are formed easily.

Consider first the problem of how folded nuclei might predominate in bulk crystallization *on the assumption that the folded structures that are observed are of homogeneous origin*. (This assumption is by no means proved in any case of bulk crystallization known to the author, but serves as a convenient starting point for the discussion.) We will deal only with crystallization near the melting point.

There is no difficulty in explaining why crystals with chain folds are deposited from a sufficiently dilute solution of a linear polymer. Lauritzen and Hoffman [3] have discussed this in terms of the rate of injection of bundle and loop type nuclei in solution which are^11^

$$I_{\text{bundle}}=I_{0}\;\exp\;\left(-\frac{\Delta H*}{kT}\right)\;\exp\;\left(-\frac{32\sigma^{2}\sigma_{e}}{{\left(\Delta f\right)}^{2}kT}\right)\times \exp\;\left(\frac{16\sigma^{2}\log_{e}v_{2}}{A_{0}{\left(\Delta f\right)}^{2}}\right)$$ (14)

$$\begin{array}{c} I_{\text{loop}}=I_{0}\;\exp\;\left(-\frac{\Delta H*}{kT}\right)\;\exp\;\left(-\frac{32\sigma^{2}\sigma_{e}}{{\left(\Delta f\right)}^{2}kT}\right)\\ \left(\text{dilute solution}\right) \end{array}$$ (15)

Equation (14) has been recast to accord with the particular notation and geometry used in the present paper. (The parameters *σ*, *σ_e_, I*_0_, ΔH*, and (Δ*f*) might have somewhat different values in dilute solution than in the bulk phase.) The quantity *v*_2_ is the volume fraction of polymer. The lateral surface free energy for the bundlelike nucleus in solution will be essentially the same as the lateral surface free energy of the loop type nucleus in solution, i.e., *σ≅****σ***. The end surface free energies *σ_e_* and ***σ****_e_* will differ to a significant extent.

The important point to note in comparing these two expressions is that, *independent of the values of σ_e_ and* ***σ****_e_ that are chosen*, *I*_loop_ *will always exceed I*_bundle_ *if v*_2_ *is taken to be sufficiently small*, *since the exponent in eg* (14) *containing* log*_e_ v*_2_ *will be a large negative number under these conditions.* Numerical estimates given in an earlier paper indicate that even if *σ_e_* is taken to be considerably smaller than ***σ****_e_*, folded nuclei will predominate at concentrations of less than about 1 to 10 percent. The term in eq (14) involving log*_e_ v*_2_ results from the fact that a number of different polymer molecules must be gathered together to form a bundlelike nucleus, whereas only a few polymer molecules (and often a single one) can form a loop type primary nucleus. Therefore, there is a large configurational entropy contribution to the formation of a bundlelike nucleus in dilute solution that does not arise in the case of the loop nucleus.^12^ In the case where *σ_e_* is larger than ***σ****_e_*, folded nuclei will predominate across the entire concentration range (see below).

For this same basic reason, a folded crystal is more stable in sufficiently dilute solution than a bundlelike one *of the same size and shape* even if *σ_e_* is smaller than ***σ****_e_.* Specifically, it can be shown [3] that the total end surface free energy per unit area for the two types of crystal may be written as 12

$$\sigma_{\text{end bundle}\;(\text{tot}.)}=\sigma_{e}-(kT/2A_{0})\;\log_{e}v_{2}$$ (16)

and

$$\sigma_{\text{end lop}\;(\text{tot}.)}=\sigma_{e}.$$ (17)

The term in eq (16) containing log*_e_ v*_2_ arises from the configurational entropy effect mentioned earlier. Thus, even for the case *σ_e_*<***σ****_e_, σ*_end bundle (tot.)_ will exceed ***σ***_end loop (tot.)_ at some low concentration of polymer, and the folded crystal will be the most stable type in dilute solution because it has less total surface free energy. In the event *σ_e_*>***σ****_e_*, the folded crystal would be the most stable type across the entire concentration range when compared with a bundlelike crystal of the same size and shape.

We are thus led to a discussion of the relative homogeneous injection rates for bundle and loop type nuclei in bulk polymers. The appropriate comparison is given by eq (15) in the case of loop nuclei, and by eq (14) with *v*_2_→1 for bundlelike nuclei (*cf.* eqs (6) and (12)):

$$I_{\text{bundle}}=I_{0}\;\exp\;\left(-\frac{\Delta H*}{kT}\right)\;\exp\;\left(-\frac{32\sigma^{2}\sigma_{e}}{{\left(\Delta f\right)}^{2}kT}\right)$$ (18)

$$\begin{array}{c} I_{\text{loop}}=I_{0}\;\exp\;\left(-\frac{\Delta H*}{kT}\right)\;\exp\;\left(-\frac{32\sigma^{2}\sigma_{e}}{{\left(\Delta f\right)}^{2}kT}\right).\\ \left(\text{bulk phase}\right) \end{array}$$ (19)

Anywhere near the melting point, the exponents involving the surface free energies in these two expressions are by far the largest and most temperature dependent. Recall also that *σ* is essentially the same as ***σ*** for the two types of nuclei. It follows that *if homogeneous nucleation of folded nuclei is to prevail over any sensible temperature range in the bulk phase*, *the condition σ_e_*>***σ****_e_ must exist.*

While the condition *σ_e_*>***σ****_e_* for homogeneous nuclei would lead to the dominance of folded nuclei (and the resultant chain folded lamellar structures) in bulk polymers, this is not necessarily the only condition that can lead to a significant number of such structures in bulk. Other factors must be taken into account. For example, chain ends may in some instances prevent the formation of large bundlelike objects, as Flory’s theory [22] suggests. (Cumulative strain may have a similar effect.) Also, heterogeneous initiation, which is certainly a very common source of spherulite initiation, must be considered. However, before discussing these points it is worth commenting on the important case *σ_e_*>***σ****_e_*.

#### Conditions giving σ_e_>**σ**_e_ (chain folding the basic mode of nucleation and growth in bulk)

It has been shown for a bundlelike nucleus with a flat end and noncumulative strain that the minimum value of *σ_e_* is given by *l_d_*(Δ*h_f_*)*ρ_c_*/6*ρ_l_*, where *l_d_* is the length of the part of the bundle end where the density falls from the crystal to the supercooled liquid value (see eq (A-20) in the appendix). Further, it was estimated in section 2.2 for the particular case of polyethylene that *σ_e_*_(min)_ was in the vicinity of 60 erg cm^−2^ on the basis of the assumption *l_d_*=10 A. An analysis of ***σ****_e_* for folded polyethylene crystals based on data on single crystals formed in dilute solution due to Keller and O’Connor [1] suggests that ***σ****_e_* is probably between 30 and 75 erg cm^−2^ [3]. More recently, a value in the vicinity of 100 erg cm^−2^ has been suggested [41]. (Although these figures apply to folded crystals formed in dilute solution, the ***σ****_e_* value relevant to folded crystals formed in bulk should not differ greatly from that appropriate to dilute solution.) Thus, while it is not altogether certain that ***σ****_e_*_(min)_ is larger than ***σ****_e_*, it is clear for the particular model used that *σ_e_* for the bundlelike nucleus can readily exceed *σ_e_*_(min)_ = 60 erg cm^−2^, since Γ will generally exceed zero. With this considered, *σ_e_* could easily be as high as several hundred erg cm^−2^. Therefore, the possibility that *σ_e_*>***σ****_e_* for polyethylene would appear to exist.

Before drawing any conclusions from the above, several points must be made clear: (1) The value of *σ_e_*_(min)_ = 60 erg cm^−2^ is admittedly based, on the assumption *l_d_*=10 A. However, it is believed that this is if anything an underestimate, so *σ_e_*_(min)_ for the flat bundle end model without cumulative strain is probably even larger than the value cited. (2) The expression *σ_e_*_(min)_=*l_d_*(Δ*h_f_*)*ρ_c_*/6*ρ_l_* depends on the assumptions used in the simplified model for the heat content and entropy as a function of density in the bundle ends. For example, a narrower maximum in Δ*H*(*ρ*) would lead to a lower value of *σ_e_*_(min)_. Nevertheless, the estimate given for *σ_e_*_(min)_ is probably not significantly high on this account, and may be too low. (3) Cumulative strain at the flat bundle ends resulting from the density difference between the “connected” liquid and crystalline phases has been omitted from the calculations. However, this will in general lead *σ_e_* to be underestimated. As will be seen subsequently in the discussion of the hypothetical bundlelike “lamellar” structure, cumulative strain will occur at flat bundle ends of large extent, especially in the case where the chain axes are perpendicular to the plane of the bundle ends. Crudely, one can think of such strain as greatly increasing Γ in eq (3), causing *σ_e_* to attain values far in excess of *σ_e_*_(min)_. We regard that it is quite certain that *σ_e_*>***σ****_e_* for large nuclei with polymer chains that are perpendicular to flat end surfaces on account of cumulative strain alone. (4) The cumulative strain in the flat bundle end model may be reduced by allowing the chains in the (still flat) surface phase to subtend a certain angle ζ with respect to those in the crystal itself (see section 4.3). (This is related to the fact that the end surface of a bundlelike crystal will tend to be curved in order to minimize the surface energy, as pointed out by Matsuoka and Maxwell [25], and Frank [26].) Estimates obtained using appropriate variations of the model treated in the appendix suggest that *σ_e_* will be fairly large for the “tilted,” model even if the cumulative strain is completely removed. The bending of the chains at a nontetrahedral angle, and the abnormal separation of the chains at the boundary nearest the crystal, produce the required contributions to the heat content in the surface phase.

With the above remarks in mind, it seems reasonable to suppose that it is entirely possible that *σ_e_*>***σ****_e_* for polyethylene for the important case of flat end surfaces. This would lead in a natural way to a predominance chain folded nuclei in bulk in this polymer, as opposed to bundlelike nuclei with flat ends. From this illustrative example, we consider that it is at least not nonsense to propose that *σ_e_* may exceed ***σ****_e_* in some linear polymers.

The free energy of formation of a bundlelike nucleus with curved ends (“ellipsoidal” model) has not been explicitly considered here. This would probably require the use of considerations akin to those proposed by Cahn and Hilliard [27] for homogeneous nucleation in systems with density gradients. This model is not considered revelant to the problem of the nucleation and growth of bundlelike “lamellae” with large flat end surfaces. Thus, while it seems reasonable on the basis of our calculations to suppose that in some cases *σ_e_*>***σ****_e_* for nuclei with flat ends, no such simple calculations will provide as much information on whether or not the free energy of formation in bulk of a folded nucleus (which of course has flat ends) is less than that of an ellipsoidal bundlelike nucleus under similar conditions. Nevertheless, our surmise would be that the necessity of having the chain molecules go through a density gradient in the ellipsoidal bundlelike case might well be able to increase its free energy of formation to the point that folded primary nuclei are still the favored type in the bulk phase. This is not certain, however, and the possibility therefore exists that ellipsoidal bundlelike nuclei may form more readily at a specified supercooling than folded nuclei, even when folded nuclei are preferred to bundlelike nuclei with flat end faces. However, if strain or chain ends limited bundlelike growth, the macroscopically observable crystals would still be of the folded type even though coexisting with a number of bundlelike microcrystals or embryos (see below).

#### Possibility of chain folds in case σ_e_<**σ**_e_

We now mention certain conditions that might lead to the appearance of a considerable amount of chain folded material in a bulk polymer even in the case where *σ_e_*<***σ****_e_* for primary nuclei.

Consider the effect of chain ends on the formation of the two types of nuclei (or crystals) under discussion, namely the bundlelike and the folded. We deal principally with the case where the chain ends are assumed to be sufficiently large so that the majority cannot enter the crystal.

It is reasonable to ignore the effect of even large chain ends in constructing the expression for the free energy of formation of relatively small bundlelike *nuclei* of a polymer of high molecular weight. However, this is not the case for bundlelike *crystallites* where large chain ends are excluded. Flory has clearly indicated for this case that there are restrictions of an equilibrium character on the size of the bundlelike polymer crystals [22]. The restriction discussed explicitly in Flory’s paper refers to a limitation on the mean length of the crystallites. Flory’s theory of bundlelike crystallization also implies a limitation on the mean radius of the crystallites. The net result is that a large number of bundlelike microcrystals of varying sizes is predicted to exist in a polymer with a distribution of molecular weight. The restriction on the ultimate (equilibrium) size of bundlelike crystallites must be given consideration in dealing with the formation of large nuclei, and the growth of crystallites, in the bundlelike pattern. The description of bundlelike microcrystallinity just mentioned does not appear to suggest the existence of large numbers of lamellae of uniform thickness and extended “radial” dimensions of the type seen in lamellar spherulites.

Even the assumption that chain ends are totally excluded from the crystal will not cause folded nuclei or thin folded crystals to fail to grow to large dimensions in the “radial” direction, i.e., in the direction normal to the polymer chain axes. (Once formed, a folded crystal will grow slowly, if at all, in the chain axis direction because of the existence of the folds, and the slowness of the requisite internal “lengthwise” diffusion mechanism.) A chain end can readily be “denucleated” the short distance to the plane of the chain folds when it finds itself on the lateral (growing) surface, thus being rapidly and efficiently excluded from the interior of the folded crystal. Such chain ends would protrude outward from the fold plane on a short section of polymer chain.

Thus, thin chain folded crystals, once nucleated, should be able to grow to large “radial” dimensions, corresponding to a certain fraction or even the entire radius of a spherulite. The main limitation would arise with low molecular weight material where the molecular length was less than twice the step height, but all molecules that were significantly longer than this would be potentially crystallizable in a basically chain folded manner. Some of the chains (including rather short ones) may be included bundle fashion in the otherwise folded crystal.

The concept that chain ends are excluded from polymer crystals must not be pressed too far, especially in the case of small chain ends. A substantial number of sufficiently small chain ends may enter chain type crystals. (This evidently applies in the case of the —CH_3_ end groups in the solid solutions formed by the *n*-paraffins of different lengths.) In the case where a certain number per unit volume of such ends can be assimilated by the polymer crystal, the restrictions noted in Flory’s theory on the size of bundlelike crystals would be relaxed, i.e., larger crystallites would form. Similarly, any restrictions on the ultimate size of the step height due to finite molecular length would be minimized, as noted earlier in section 2.5.

The radial growth of bundlelike nuclei may be significantly reduced or even stopped by cumulative strain at the bundle ends resulting from the density difference of the liquid and crystalline phases. As mentioned earlier, this will certainly be the case for bundlelike nuclei with flat ends where the chain axes are perpendicular to the end face. This may possibly even occur for ellipsoidal bundlelike nuclei, or bundlelike nuclei with flat ends where the chain axes are inclined at an angle to the end surface. In such situations, the condition *σ_e_*<***σ****_e_* might exist for small nuclei or very small crystals, while cumulative strain leads to *σ_e_*>***σ****_e_* for large nuclei or crystals. Homogeneous nucleation of tiny bundlelike nuclei would then prevail over the folded type, but the crystals apparent on a large scale in the system would be formed on a basically chain folded pattern.

Even in the case where *σ_e_*<***σ****_e_* for small nuclei, and *σ_e_*>***σ****_e_* for large nuclei, the rate of homogeneous nucleation of lamellar spherulites with chain folds will be given approximately by eq (19), especially at moderate to low supercooling where the nuclei will be rather large. The nucleus, though starting on the bundlelike pattern, would begin folding as it grew and exhibit the overall energetics characteristic of a folded nucleus. At high supercooling, stable bundlelike nuclei would appear in profusion, though folding would occur as these grew to large size. In this region, the apparent homogeneous injection rate of folded objects would increase above that given by eq (19). This effect may strongly resemble the onset of region B.

The above remarks form the basis of the conception that even if *σ_e_*<***σ****_e_* for *small* nuclei, chain folded structures may under certain circumstances be the most prevalent and physically obvious form of bulk crystallization in linear polymers. Specifically, cumulative strain or sufficiently large chain ends may poison the coherent growth of bundlelike crystals to large dimensions, causing any strictly bundlelike crystals that were initiated to be of limited size: then any folded structures that were introduced into the system through the agency of either homogeneous or heterogeneous nucleation might be able to grow to very large size in the two dimensions normal to the chain axes in the same general sense that folded single crystals do in dilute solution. In this case, it is important to distinguish between the prevalence and ease of observation of a certain type of crystal on the one hand, and the rate of homogeneous initiation of small nuclei of limited growth potential on the other. For the case where *σ_e_*>***σ****_e_* for nuclei, the formation of folded structures will dominate the bulk crystallization process.

The remaining point concerning the origin of nuclei deals with the possibility of heterogeneous nucleation. In real polymer systems, the initiation of spherulites is for the most part of pseudohomogeneous or heterogeneous origin. (Pseudohomogeneous initiation, which refers to the essentially sporadic initiation events that may take place on the flat surfaces of weakly wettable heterogeneities, can imitate the truly sporadic initiation characteristic of homogeneous initiation.) As a result of special interactions, heterogeneities might conceivably have a strong predilection for producing structures containing loops on their surfaces, even if *σ_e_*<***σ****_e_* for the homogeneous process. This might cause chain folded structures to be prevalent in linear polymers crystallized in bulk. In the case where *σ_e_*>***σ****_e_*, chain folded structures of heterogeneous or pseudohomogeneous origin would arise in numbers far in excess of that characteristic of homogeneous nucleation if suitable heterogeneities were present, and these structures would certainly be the dominant form present. Certain questions relating to homogeneous, pseudohomogeneous, and heterogeneous nucleation will be discussed subsequently in section 8.

#### Summary

The following conclusions may be drawn. If folded nuclei are to predominate in the supercooled bulk phase of a polymer on a *homogeneous* basis over any important range of temperature, the end surface free energy of the bundlelike nucleus must for some reason exceed that of a folded nucleus (*σ_e_*>***σ****_e_*). (This statement refers specifically to nuclei with flat end surfaces.) Because of a certain flexibility in the parameters that define *σ_e_* and ***σ****_e_*, it is not possible to say *a priori* that homogeneous formation of folded nuclei should dominate the bulk nucleation mechanism. However, a plausible case can be made for supposing that *σ_e_* might well exceed ***σ****_e_* under certain circumstances. In any event, the quantity ***σ****_e_* is evidently considerably larger than has been assumed in the past. (A compelling theoretical case can be made for the dominance of folded nuclei of homogeneous origin in sufficiently dilute solution.) In the case of heterogeneous nucleation under the condition *σ_e_*>***σ****_e_* folded structures are to be expected. It is probably not absolutely necessary that *σ_e_* always be greater than ***σ****_e_* to have a significant amount of folded structures appear in bulk. Sufficiently large chain ends may poison the formation of large bundlelike crystallites, but at the same time not seriously affect the formation of large chain folded objects. Similarly, cumulative strain may abort the growth of bundlelike crystals; this corresponds to *σ_e_*<***σ****_e_* for small bundlelike nuclei, and *σ_e_*>***σ****_e_* for large bundlelike nuclei or crystals. Further, heterogeneities, a common source of spherulite initiation, might induce folded surface nuclei by specialized interactions.

Attention is now directed to the main problem of the rate of radial growth of spherulites, and the connection between the proposed mechanisms and spherulite structure.

## ★3. Rate of Radial Growth of Spherulites: Preliminary Considerations

### 3.1. The Two Types of Nucleation Problem in Spherulitic Growth

We will repeatedly encounter two types of nucleation problem in connection with the rate of radial growth of a spherulite. The first of these is quite similar to that already treated for primary nuclei in the previous sections, in that it deals with a nucleus that is built up, step by step, until a critical size is finally reached. Nucleation of this type has already been treated by Turnbull and Fisher [18], and is readily adapted to deal with surface nuclei that are gradually built up to critical size in a stepwise manner. The second type of nucleation problem commonly encountered is that where the maximum in the free energy barrier is reached in a single step, rather than in a large number of steps. This problem often arises when nucleation of a monomolecular layer is considered. The problem of monomolecular layer growth has been considered by Lauritzen and Hoffman [3].

**Figure 3:**
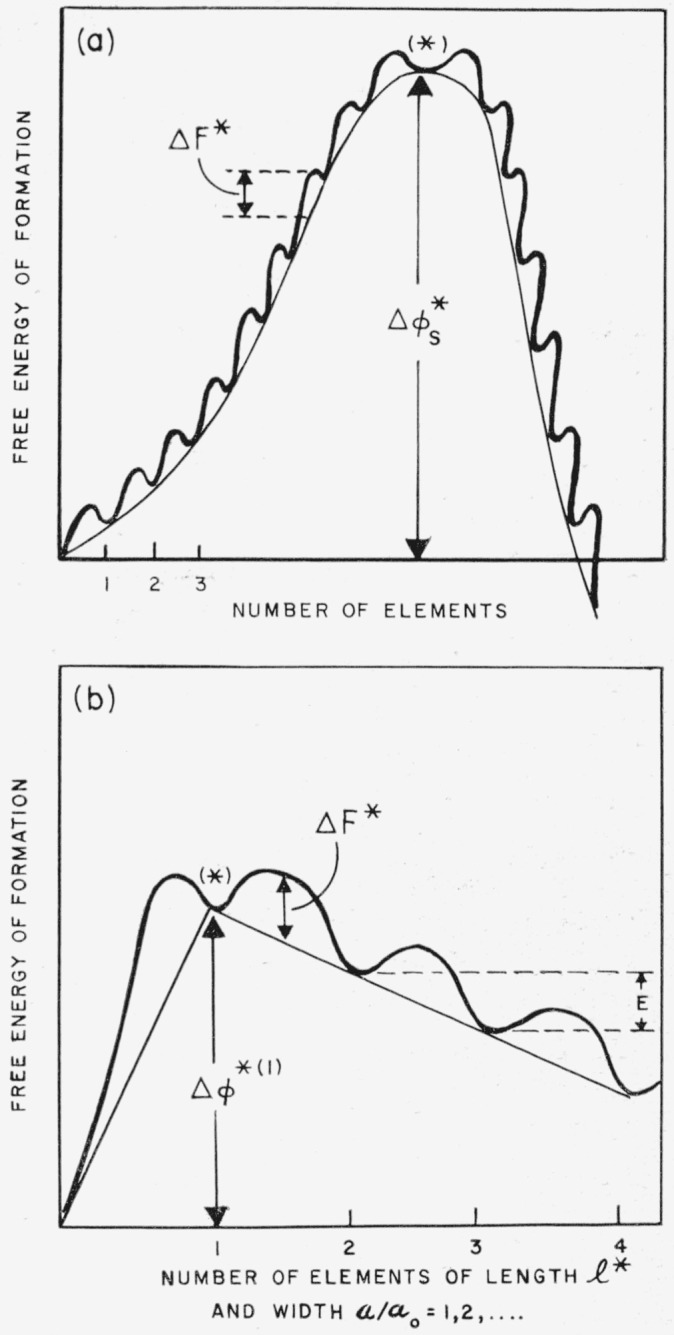
The two types of nucleation problem encountered in calculating the rate of radial growth of polymer spherulites. (a) Activated state (*) reached by successive addition of a large number of ele ments (Turnbull and Fisher, continuum free energy surface model). (b) Activated state (*) reached in single step, i.e., by the accretion of one element of length l*, as in certain cases of monomolecular layer growth (Lauritzen and Hoffman, discrete free energy surface model).

If the surface nucleus is built up by successive addition of a large number of elements until a nucleus of critical size is reached, the free energy of formation may be represented as in figure 3a. Each elementary forward reaction has a reaction rate of the form (*kT*/*h*) exp (*−w_f_*/*kT*), and each backward reaction a rate of the form (*kT*/*h*) exp (*−w_b_*/*kT*). Then by summing over all the forward and backward reactions in the manner described by Turnbull and Fisher, it is found that the overall rate of nucleation per unit area of substrate is

$$I_{s}=I_{s(0)}\;\exp\;\left(-\frac{\Delta F*}{kT}\right)\;\exp\;\left(-\frac{\Delta \phi_{s}^{*}}{kT}\right),$$ (20)

where ΔF* is the free energy of activation of the interfacial jump rate, $\Delta \phi_{s}^{*}$ the work required to build a nucleus of critical size, and *I_s_*_(0)_ a factor that does not depend strongly on temperature. As before, the term in ΔF* may be broken up to give ΔH*–*T*ΔS*. The quantity $\Delta \phi_{s}^{*}$ is, in general, calculated in a manner similar to that used previously for primary nuclei, i.e., the free energy surface is treated as a continuum. Each nucleus then rapidly grows across the substrate crystal and produces a new layer, or substantial fraction thereof. A new surface nucleus will then form on this new layer. Accordingly, the rate of growth on this crystal face depends on the rate of nucleation on the face. When the crystal face in question corresponds to that leading to radial growth of the spherulite the steady state rate of radial growth may be written

$$G=dr/dt=G_{0}\;\exp\;\left(-\frac{\Delta H*}{kT}\right)\;\exp\;\left(-\frac{\Delta \phi_{s}^{*}}{kT}\right).$$ (21)

(large surface nuclei built up step by step; $\Delta \phi_{s}^{*}$ calculated from continuum model of free energy surface)

Here *G*_0_ is a constant with the dimensions *lt*^−1^*, r* the radius of the spherulite at time *t.* The quantity *G*_0_ contains the factor exp (ΔS*/*k*), and certain relatively unimportant geometrical factors. The principal problem connected with calculating the radial growth rate for a model is the evaluation of $\Delta \phi_{s}^{*}$. As in the case of homogeneous nucleation, $\Delta \phi_{s}^{*}$ is the free energy at the saddle point described by the appropriate free energy function.

At low to moderate supercooling, the main temperature dependence in eq (21) is due to the $\Delta \phi_{s}^{*}$ term, the term in ΔH* being next in importance. The temperature dependence of *G*_0_ is negligible in comparison.^13^ For steady state surface nucleation, the term in $\Delta \phi_{s}^{*}$ always has a negative temperature coefficient. The term in ΔH* has the usual positive temperature coefficient.

The problem takes on a somewhat different character in cases where the activated state is reached in one step (fig. 3b). Although from a formal standpoint the free energy surface in question may have a saddle point, it is not correct to treat the free energy of formation Δ*ϕ* as a continuous function of the width *a* of the nucleus when the activated state is reached at *a*=*a*_0_. Then Δ*ϕ* is defined only for discrete values of *a.* The net rate is maximized when the length of the step element has a critical length l*, the nucleus being formed by passage over part of the barrier ridge which is not necessarily at the saddle point in the free energy surface treated as a continuum. For this problem, a summing of all the forward and backward reactions leads to a nucleation rate per unit area of surface of the form [3]:

$$I_{s}=I_{s(1)}\;\left[\frac{\sinh\;(E/2kT)}{1+\;\exp\;(-\Delta \phi_{s}^{*(1)}/2kT)\;\sinh\;(E/2kT)}\right]\times \;\exp\;\left[-\frac{\Delta H*}{kT}\right]\;\exp\;\left[-\frac{\Delta \phi_{s}^{*(1)}}{kT}\right].$$ (22)

Here $\Delta \phi_{s}^{*(1)}$ is the free energy function Δ*ϕ*_s_ evaluated for the case *v*=1, where *v* is the number of elements (i.e., the number of chains of critical length l*) laid side by side on the crystal substrate, and *E* the incremental increase of stability on adding each new element (see fig. 3b).

In the case under consideration, the activated state is reached when one such element of length l* attaches to the surface. This is what is meant by the statement that the activated state is reached in “one step”; the term “one step” does not refer here to the elementary process where a single polymer segment is laid down during the process of building up the length l*. The value of ΔF* and ΔH* relevant to the interfacial jump rate characteristic of the laying down of an entire step element of length l* may be larger than $\Delta F_{\mathrm{seg}}^{*}$ and $\Delta H_{\mathrm{seg}}^{*}$, which refer to the jump rate at the interface for the elementary segmental processes. The principal contribution to ΔF* and ΔH* probably comes from jump rate processes at the end of the nucleus, especially in the case of folded systems.

In most cases of practical interest, the term in brackets involving sinh (*E*/2*kT*) will be sufficiently independent of temperature to be taken into the pre-exponential. Then by applying the same arguments used earlier, *I_s_* may be transformed into an expression for the radial growth rate of a spherulite:

$$G=dr/dt=G_{0(1)}\;\exp\;\left(-\frac{\Delta H*}{kT}\right)\;\exp\;\left(-\frac{\Delta \phi_{s}^{*(1)}}{kT}\right).$$ (23)

(small surface nuclei where activated state is reached in single step; Δϕ*^(1)^ calculated from discrete free energy surface model)

It is emphasized that eq (23) frequently applies in the important case of growth by addition of monomolecular layers. If eq (21) is inadvertently applied in such instances, misleading or even erroneous results may be obtained; it is important not to confuse $\Delta \phi_{s}^{*}$ (continuum model) with $\Delta \phi_{s}^{*(1)}$ (discrete model) in calculating the work required to form the nucleus of critical size.

The calculations of the radial growth rate of spherulites to be given in this paper refers to experiments where an unoriented polymer is *first* heated well above *T_m_* and *then* rapidly cooled to the growth temperature. The less readily interpretable experiments where specimens are first quenched from the melt and then rewarmed to the growth temperature are discussed briefly in section 8.2.

### 3.2. The Jump Rate Term in Supercooled Liquids

Another point of interest is that the interfacial jump rate term may require modification, particularly if it depends strongly on segmental motions in the supercooled liquid polymer. If such motions dominate the interfacial jump rate process, it can be shown that the term exp [−ΔH*/*kT*] in the various equations that have been given may be replaced by the empirical expression based on the work of Williams, Landel, and Ferry [28]

$$\;\exp\;[4.12\times 10^{3}T^{2}/RT{\left(51.6+T-T_{g}\right)}^{2}]$$ (24)

that is valid between *T_g_* and *T_g_*+100°. Here *T_g_* is the glass transformation temperature. This is equivalent to the statement that ΔH* depends on temperature, and has the value 4.12 × 10^3^*T*^2^/(51.6 + *T−T_g_*)^2^. For the sake of simplicity, and for the reason that crystallization temperatures of interest often lie above *T*g+100°, we have not employed this empirical expression in the body of the paper. Near the melting point, the constant value ΔH*=20,000 cal mole^−1^ may be employed for trial purposes.

## 4. Polymer Crystal Growth by Coherent Bundlelike Surface Nucleation

### 4.1. Model

**Figure 4:**
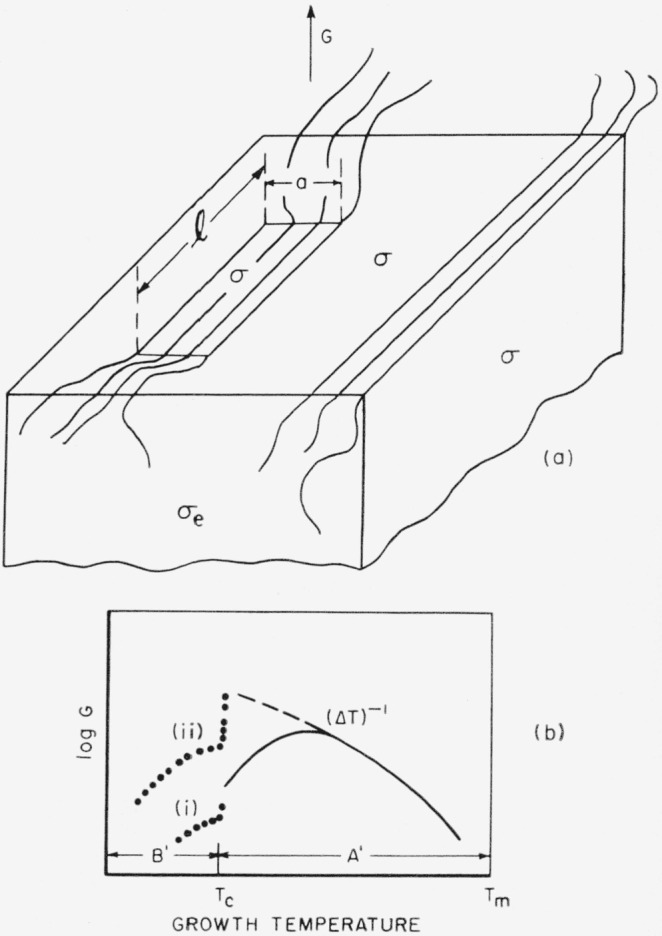
The coherent bundlelike surface nucleus model and its crystal growth rate behavior. (a) Coherent nucleus of length *l*, width *a*, and monomolecular layer thickness *b*_0_ on substrate crystal. Heavy arrow marked *G* indicates direction of crystal growth. (b) The logarithm of growth rate versus temperature, — — — — log *G* versus *T* if effect of jump rate is small; ▬▬ behavior where jump rate lowers log G causing maximum to appear in log *G* versus *T;*…. possible effect of interference with growth by excess nucleation in surrounding medium (cf. fig. 1(a)).

The model used is shown in figure 4a. The polymer molecules in the nucleus are colinear with those in the substrate, i.e., the surface nucleus is of the *coherent* type. The thickness of the surface nucleus is *b*_0_, corresponding to one molecular layer.^14^ Once formed, this nucleus, which has a length *l* and width *a*, leads to the rapid completion of a layer of thickness *b*_0_ on some large area of the growing face. Note that the molecules are oriented at a right angle to the direction of growth, which is marked “*G*”. The lateral surface free energy is *σ*, and the end surface free energy is *σ_e_.*

The coherent bundlelike surface nucleus model (or one of several simple modifications of it) has been presented at various places in the literature as a pattern for typical spherulitic growth. However, calculations on this model should be approached with full recognition of the fact that it is very improbable that it will lead to a typical lamellar spherulite. The relationship of this model to the structure of spherulites will be discussed shortly. Meanwhile, we shall treat the model as if it did produce a large crystal for which one could define a radial growth rate *G.*

### 4.2. Growth Rate for Bundlelike Coherent Nucleus Model

A prime will be used to distinguish the quantities connected with coherent surface nuclei from those belonging to primary nuclei. Later, a double prime will be used to denote quantities related to noncoherent nuclei.

#### Region A′

In region *A*′, i.e., from temperatures near *T_m_* on down to those corresponding to rather high supercooling, *a* and *l* may be regarded as not having reached their minimal values, and the free energy of formation may be written as

$$\Delta \phi =2ab_{0}\sigma_{e}+2b_{0}l\sigma -ab_{0}l(\Delta f).$$ (25)

Notice that no term involving *al* appears in this expression: for a strictly coherent nucleus, only the work required to build the sides enters. By setting (∂Δ*ϕ*/∂*a*)*_l_* and (∂Δ*ϕ*/∂*l*)*_a_* equal to zero, it is determined that a* = 2*σ*/(Δ*f*) and l* = 2*σ_e_*(Δ*f*). Then it is found that

$$\Delta \phi *=\frac{4b_{0}\sigma \sigma_{e}}{\left(\Delta f\right)}.$$ (26)

Since *a* and *l* are variables, so that the surface nucleus is not formed in one step, *G* is to be calculated with eq (21):

$$\begin{array}{c} G_{A\prime }=G_{0}\;\exp\;\left(-\frac{\Delta H*}{kT}\right)\;\exp\;\left(-\frac{4b_{0}\sigma \sigma {\;}_{e}T_{m}^{2}}{T(\Delta h_{f})(\Delta T)kT}\right).\\ \left[{\left(\Delta T\right)}^{-1}\;\text{law}\right] \end{array}$$ (27)

#### Region B′

The question must now be raised as to what behavior must be expected of the radial growth rate at high degrees of supercooling, provided that the jump rate term has not already caused *G* to fall to a low value.

Suppose that the crystals whose radial growth rate we have discussed were of heterogeneous origin, so that in region *A'* they were born near *t* = 0. Then at the *T_c_* transition in the supercooled bulk phase, a vast number of tiny bundlelike nuclei would be injected by the nonsteady state nucleation mechanism described in section 2.3 into the matrix in which the crystal was attempting to grow. Thus, at or near the *T_c_* transition characteristic of the pure bulk phase, which will take place at Δ*T_c_* ≅ 4*σT_m_*/(Δ*h_f_*)*a*_min_, the radial growth of the older and larger crystals would be rather abruptly slowed down because of depletion of crystallizable material, and impingements.

If it is assumed that the crystallites are of entirely homogeneous origin, a rather similar phenomenon can take place. Here *I_A_*, as calculated for a bundlelike nucleus in section 2 may be regarded as being proportional to the rate of injection. Barring interference from the jump rate term, the injection rate would rather abruptly attain a value considerably in excess of the extrapolated value of *I_A_*. Such a high injection rate at and below *T_c_* due to nonsteady state nucleation would lead to a massive number of impingements. Any crystallites formed in these circumstances would tend to be small, and have a significantly reduced growth rate.

#### Summary

A schematic diagram of the variation with temperature of the radial growth rate of a body that is governed by a coherent bundlelike surface nucleus is shown in figure 4b. Curves (i) and (ii) in region *B*′ are intended to represent different degrees of interference with the growth resulting from the incursion of a vast number of competing microcrystals resulting from nonsteady state nucleation. The solid line in region *A′*, which exhibits a maximum in *G* because of the effect of the jump rate term, shows the type of behavior that is most probable if the model is valid.

It is considered that the treatment of the secondary (coherent) bundlelike surface nucleus outlined in section 4.2 is more fully illustrative than that given previously using the customary pillbox surface nucleus (cf. reference [11]). The pillbox nucleus requires only a single surface free energy that corresponds to our *σ*, and leads to a nucleation term involving *σ*^2^. For a surface nucleus consisting of chain molecules lying on a flat surface, it seems better to distinguish between the lateral surfaces, and the end surface, giving a nucleation term involving the product *σσ_e_*. If the growth nucleus were assumed to form on the end of the bundle, so that the direction of growth was parallel to the polymer chains, then the pillbox nucleus with its single lateral surface free energy would be justified. However, this model would not lead to a “spherulite” where the polymer chains were normal to the radius.

### 4.3. Coherent Bundlelike Growth and Spherulite Structure

Though much attention has been given here and elsewhere to various coherent bundlelike growth nucleus models, it remains to be seen whether or not such a growth mechanism would lead to a roughly spherical object that would be recognized as a spherulite. When this is done, it will emerge that one must have strong reservations about the ability of the model to reproduce anything resembling a typical lamellar spherulite.

**Figure 5:**
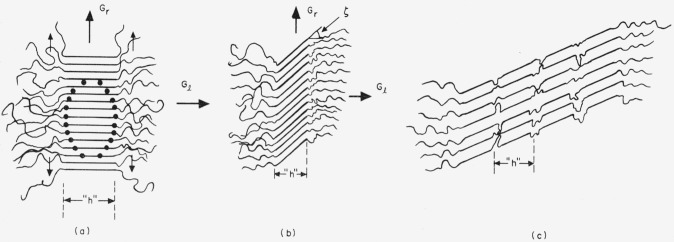
Hypothetical structure of bundlelike lamellae. (a) Isolated lamella with chains perpendicular to flat bundle ends. Light arrows show site of cumulative strain, “h” represents hypothetical step height. Dotted lines illustrate how crystal would round off end to reduce strain and total surface free energy. (b) Isolated lamella with chains tilted with respect to flat bundle end. Cumulative strain is reduced, but “lamella” will grow in both *G_l_* and *G_r_* directions. (c) Stacks of interconnected bundlelike “lamellae” with tilted chains.

Suppose that we attempt to construct a single “lamella” on the strictly bundlelike pattern of the type shown in figure 5(a) where the polymer chain axes are perpendicular to the *σ_e_* plane. It is simply not possible to create a large flat surface of the type commonly seen in lamellar spherulites in this manner because of the density difference between the supercooled liquid and crystalline phases, and the fact that polymer molecules with covalent bonds connect these two phases in the bundlelike system of crystallization. Growth in the “radial” direction, *G_r_*, for such a hypothetical “lamella” would clearly result in cumulative strain at the bundle ends. As pointed out by Matsuoka and Maxwell [25], and Frank [26], the crystal would actually tend to become ellipsoidal (see dotted lines in figure 5(a)) in order to minimize the total surface free energy. If the bundle end were assumed to remain flat, the effective value of *σ_e_* would increase enormously as the crystal grew in the *G_r_* direction, thus aborting its growth.

This effect should become distinctly apparent for nuclei where the radius corresponds to a mismatch between the supercooled liquid and crystal of one molecular diameter. A conservative estimate would place this radius at less than 50 A under normal circumstances. The coherent bundlelike growth model where the polymer chains in the crystal are perpendicular to the hypothetical *σ_e_* plane is clearly defective in its ability to predict anything resembling a typical lamella.

It might be suggested that the cumulative strain problem discussed above for the perpendicular case can be minimized by allowing the chains on the faces of a flat bundlelike lamella to exit from the crystal at an angle *ζ*. In such a “lamella”, the chain axes would thus be tilted with respect to the presumably flat surface of the end, as shown schematically in figure 5(b). The idea is that the chains in the surface region open up to spacings *a*_0_+Δ*a*_0_ and *b*_0_+Δ*b*_0_ that closely corresponds to the mean molecular spacing in the supercooled liquid polymer. It is not entirely clear that this would relieve the cumulative strain of the type that exists in the perpendicular case if the *σ_e_* surface must be kept flat. It must be remembered that the “lamella” will be three dimensional, i.e., both the *a* and *b* spacings in the surface phase must be larger than *a*_0_ and *b*_0_. It seems likely that the end surface would tend to become curved. Even ignoring these effects, it is clear that the work required to bend the polymer bonds at the required angle, and to increase the spacings in the surface phase, will lead to at least a fairly large *σ_e_* value from noncumulative strain as noted in section 2.6. As will be demonstrated below, a lamella with a sensible end surface free energy that is not subject to cumulative strain will not maintain its “step height” or “lamellar” thickness.

Consider now the relative growth rates in the radial (*r*) and lengthwise (*l*) directions for a bundlelike crystal of the type illustrated in figures 5(a) and 5(b) on the assumption that cumulative strain is absent. The growth in the radial direction has already been calculated:

$$G_{r}=G_{0}\;\exp\;\left(-\frac{\Delta H*}{kT}\right)\;\exp\;\left(-\frac{4b_{0}\sigma \sigma_{e}T_{m}^{2}}{T(\Delta h_{f})(\Delta T)kT}\right).$$ (28)

The corresponding growth rate on the *σ_e_* faces of the “lamella” is

$$G_{l}=G_{0}\;\exp\;\left(-\frac{\Delta H*}{kT}\right)\;\exp\;\left(-\frac{4l_{\min}\sigma^{2}T_{m}^{2}}{T(\Delta h_{f})(\Delta T)kT}\right),$$ (29)

where *l*_min_ is the length of the coherent surface nucleus that forms on the bundle end. (If cumulative strain occurred in the bundle ends, both *σ* and *σ_e_* would increase rapidly with coherent growth in the *r* and *l* directions, and the crystal would not grow to form a large crystal like a lamella, but would form instead an ellipsoidal bundlelike microcrystal of limited size as noted in section 7.)

The quantity *l*_min_ may be taken as the minimum length of polymer chain that is crystallizable. It is impossible to escape the fact that the bundlelike crystal will grow at a sensible rate on the *σ_e_* face if growth on the other surfaces is rapid at the same supercooling. Even if *l*_min_ is assumed to be several times larger than *b*_0_, it must be remembered that *σ_e_* will probably be substantially larger than *σ.* Thus we must expect *G_l_* to be roughly comparable to *G_r_*, or possibly even considerably larger. In such a case the bundlelike “lamella” would not maintain its hypothetical step height “*h*” as radial growth progressed. The above calculation accords with the concept that a high energy surface will grow more rapidly than a low energy one.^15^ It may be concluded that an isolated “lamella” that maintained its thickness or “step height” cannot be predicted with either of the bundlelike models depicted in figure 5(a) and 5(b).^16^

We consider it preferable to consider only theories of polymer crystal growth capable of predicting the existence of an isolated lamella in a straight forward manner. It is then a simple manner to generalize to the case where the lamellae occur in stacks. However, there is one rather arbitrary model involving stacks of bundlelike “lamellae” that deserves comment. This is shown in figure 5(c). Here one polymer molecule participates in more than one sheet, with entangled and “amorphous” polymer between. By drawing the model as shown, i.e., with chains tilted with respect to the *σ_e_* plane, the question of cumulative strain is supposedly minimized. Also the point that bundlelike lamellae will grow on the *σ_e_* face is temporarily evaded, except at the outer edges. This model of “lamellar” structure suffers from some drawbacks. First, there would appear to be no simple way to predict the formation of such a structure on theoretical grounds. Second, it is difficult to discern how such a structure could lead to the fracture or surface replica patterns, with many easily distinguishable steps, that characterize lamellar spherulites. To do this, it would apparently be necessary to assume that covalent bonds broke only in the amorphous regions. It seems much more likely that relatively few covalent bonds are broken, and that the lamellar separation is opposed mainly by van der Waals forces, for example those at two chain folded surfaces. Finally, the tipping of crystals at low draw ratios is difficult to explain with the model.

The bundlelike models for “lamellae” criticized above are all in the category where the flat “lamellar” surfaces are assumed to be of the *σ_e_* type, i.e., where polymer chains protrude from the surface. We now consider briefly the possibility that the flat faces of the lamellae might correspond to polymer chains that lie bundle fashion parallel to the large flat faces, but where the chains still subtend a right angle to the radius of the spherulite. Entirely apart from the fact that there is already some evidence that the polymer chains are essentially normal to the large flat lamellar faces (see [4] and [42]), this is an unlikely model for a stable lamella of uniform step height. The flat faces would be *σ* type, and would therefore grow normal to the spherulite radius at a rate comparable to the radial growth rate, as implied by eq (28), if cumulative strain did not occur. If cumulative strain did occur, large lamellae with large flat *σ*-type faces would not be formed since the crystallites would be of limited size, and tend to be ellipsoidal in shape.

Our conclusion is that the coherent bundlelike surface nucleus model does not lead to a tenable representation of a typical lamellar spherulite, where large stacks of more or less separable lamellae comprising the major fraction of the crystallization present exist, and where at a fixed growth temperature each lamella has a relatively uniform step height even with extended growth.

The remaining question concerns whether the coherent bundlelike model of polymer crystal growth can lead to a spherical object that is not lamellar. For the case of large chain ends that are excluded from the crystal, this theory appears to lead to microcrystals scattered here and there throughout the medium, the equilibrium dimensions of these microcrystals being determined by the distribution of molecular weight (see sec. 2.5). Similarly, cumulative strain would lead to ellipsoidal microcrystals of limited size. For the strictly coherent case, there is no reason to suppose that these microcrystals would be arranged into a spherical array. With the additional assumption of noncoherent nucleation on the surfaces, a spherical array of bundlelike but nonlamellar microcrystals can be predicted (sec. 7).

## ★ 5. Radial Spherulitic Growth by Coherent Surface Nucleation With Chain Folds

### 5.1. Details of the Model and Derivation of the Radial Growth Rate Law

**Figure 6:**
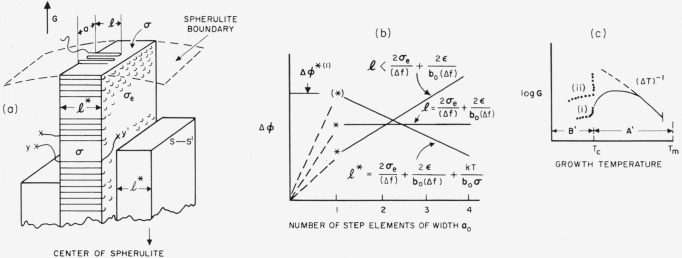
The coherent chain folded surface nucleus model of spherulitic growth and its rate behavior. (a)Lamella with folded coherent nucleus of length **l**, width **a**, and monomolecular layer thickness **b**_0_ on its surface. Heavy arrow marked **G**, indicates direction of radial growth of lamellar spherulite. Arrow s−s′ indicates axis of spiral dislocation. Chain y−y′ indicates molecule incorporated bundle fashion into lamella. (b)Schematic diagram illustrating cause of a certain value of the step height, l*, giving maximum surface nucleation rate. (c) Logarithm of radial growth rate of spherulite as function of temperature.––––log **G** versus T if jump rate effect is small;——most representative case where jump rate lowers log **G** causing maximum to appear near (T*_m_*+T*_g_*)/2;…. exhibits effect of excess nucleation rate in medium surrounding spherulite.

Here it is assumed that the rate determining step is the formation of a coherent surface nucleus of monomolecular thickness **b**_0_, length **l**, and width a on the substrate crystal (fig. 6a). The end surface free energy of this nucleus is ***σ****_e_*, and the two lateral surface free energies are each taken as ***σ***. The quantity ***σ****_e_* is defined as in eq (1), and ***σ*** has the same meaning as in section 2.4. Both of these surface free energies refer to abrupt phase boundaries, and are well defined quantities.

The objective is to calculate the rate of nucleation on the growing face of the lamella, since this establishes the radial growth rate of the spherulite (the direction of radial growth is denoted by the heavy arrow marked **“G”**). The growing face may actually be wedge-shaped, the face on either side of the peak having a surface free energy ***σ***. The assumption of such a geometry would not materially alter the calculations to be given, and it is sufficient for the purpose of calculating the radial growth rate to consider the simpler model with one growing face. Note that the polymer chains are normal to the spherulite radius.

The value of **l** for the initial substrate could be assumed to be that of a primary nucleus, $l_{p}^{*}=4\sigma_{e}/\left(\Delta f\right)$, but this is not necessary for the treatment: one could equally well assume heterogeneous initiation. The model leads in a natural way to many features of lamellar spherulites. The connection between this model and spherulite structure will be discussed in section 5.4.

#### Region **A**′

The free energy of formation of the surface nucleus is

$$\Delta \phi =2ab_{0}\sigma_{e}+2b_{0}l\sigma +2a\epsilon -ab_{0}l\left(\Delta f\right).$$ (30)

The edge free energy ***ϵ*** is included to take account of the possibility that a loop in a surface nucleus where **l**≤l* may be more difficult to form than one where **l**>l*.^17^ The inclusion of the edge free energy leads to only a slight complication of the results, and is useful in discussing the step height and melting behavior of the crystal lamellae that will be observed (see sec. 5.2).

The treatment for the coherent surface nucleus with chain folds differs significantly from that given for the corresponding bundlelike nucleus. The quantity **l** will not change rapidly with growth, and is to be regarded as fixed for a given nucleus as it traverses the nucleation path. (Other folded nuclei may of course have different values of **l**, but in each case **l** is constant for a given nucleus.) In this situation, the barrier ridge that must be overcome to form a stable surface nucleus is at **a**=**a**_0_. Thus, during the formation process for a given nucleus, two of its dimensions, namely, **b=b_0_** and l=l*, are fixed. It remains to be determined what value of **l** leads to the maximum rate of steady state nucleation. This value of **l** is denoted l*.

From eq (30) it is readily determined that a growable nucleus can be formed only when **l** is slightly in excess of

$$l=\frac{2\sigma_{e}}{\left(\Delta f\right)}+\frac{2\epsilon }{b_{0}(\Delta f)}.$$ (31)

If **l** has a value less than this, the embryo becomes increasingly less stable as the width **a** is increased, as shown in figure 6b. Similarly, if **l** has exactly the value given by eq (31), no stable nucleus will be formed, since the free energy of formation is a constant and cannot ever become negative with any increase of **a** (fig. 6b). Hence l* must be slightly in excess of that given by the above expression.

The competition between two effects determines the conditions under which the maximum rate of nucleation will be observed. Notice from the schematic diagram in figure 6b that at **a**=**a**_0_ the activated state with the *highest* free energy is associated with a value of **l** that leads to the formation of a stable nucleus on further addition of step elements, and conversely, the activated state with the *lowest* free energy is associated only with the formation of a metastable embryo. (The free energy of activation at **a**=**a**_0_ is marked “*” in each instance.) Thus there is some length slightly greater than that given by eq (31) which will lead to a maximum steady state rate of nucleation.

It has been shown in detail elsewhere (see eqs (59) to (66) and appendix 5.2 of reference [3]) that

$$l*=\frac{2\sigma_{e}}{\left(\Delta f\right)}+\frac{2\epsilon }{b_{0}(\Delta f)}+\frac{kT}{b_{0}\sigma }$$ (32)

is the value of the length of the growth nucleus that leads to the maximum rate of steady state nucleation. This is, of course, the step height of the crystal that will actually be formed by the coherent nucleation process.

On inserting eq (32) into (30) with **a** = **a**_0_, the free energy of formation is found to be

$$\Delta \phi^{*(1)}=\frac{4(b_{0}\sigma \sigma_{e}+\sigma \epsilon )}{\left(\Delta f\right)}-\frac{a_{0}(\Delta f)kT}{\sigma }+2kT.$$ (33)

The second two terms in eq (33) arise from the term *kT*/**b**_0_***σ*** in eq (32). On inserting Δϕ^*(1)^ into eq (23), there is obtained

$$G_{A\prime }=G_{0(1)}\;\exp\;\left(-\frac{\Delta H*}{kT}\right)\;\exp\;\left(-\frac{4b_{0}\sigma [\sigma_{e}+(\epsilon /b_{0})]T_{m}^{2}}{T(\Delta h_{f})(\Delta T)kT}+\frac{a_{0}(\Delta h_{f})(\Delta T)T}{T_{m}^{2}\sigma }\right),$$ (34a)

if the unimportant factor *e*^2^ arising from 2*kT* in eq (33) is ignored. An evaluation of the term exp $\left[a_{0}(\Delta h_{f})(\Delta T)T/T_{m}^{2}\sigma \right]$ shows that it comes to approximately exp [(Δ*T*)/100] for normal values of the parameters. This dependence on temperature is completely negligible compared to the other two exponential terms in eq (34a), and the term may safely be taken into the pre-exponential factor **G**_0(1)_. Then we have as a good approximation the expression

$$G_{A\prime }=G_{0(1)}\;\exp\;\left(-\frac{\Delta H*}{kT}\right)\;\exp\;\left(-\frac{4b_{0}\sigma [\sigma_{e}+(\epsilon /b_{0})]T_{m}^{2}}{T(\Delta h_{f})(\Delta T)kT}\right)\;[{\left(\Delta T\right)}^{-1}\;\text{law}]$$ (34b)

for the rate of growth of chain folded systems in region **A′**, which extends from near *T_m_* down to *T_c_* (see below). The term ***ϵ*/b**_0_ will not exceed ***σ****_e_* in magnitude, and may be considerably smaller.

It is somewhat misleading to derive eq (34b) by differentiating eq (30) with respect to **a** and **l**, setting the results equal to zero to get l* =2***σ****_e_*/(Δ*f*)+2***ϵ***/**b**_0_(Δ*f*) and a* = 2***σ***/(Δ*f*), inserting these into (30) to get Δϕ*=4(**b**_0_***σσ****_e_*+***σϵ***)/(Δ*f*), and thence to eq (34b) by way of eq (21). Calculations involving this method have been proposed in the literature in connection with chain fold growth for the case **ϵ**=0 [15]. The free energy surface is not a continuum in the region of interest as implied by the differentiation, and the value of l* obtained does not permit growth at all, as is seen in figure 6b.

At very low supercooling, the step height will become large enough so that a considerable number of the molecules cannot fold if large chain ends are excluded from the crystal, as mentioned in section 2.5.

#### Region **B′**

The rate law cited above will be valid from near *T_m_* on down to a temperature **T***_c_* corresponding to a degree of supercooling of approximately Δ*T_c_*=4***σ****T_m_*/(*Δh_f_*)**a**_min_. At this temperature and below, folded nuclei of minimal size of the type described in section 2.5 will appear in profusion in the supercooled medium due to nonsteady state nucleation, and slow up or even stop the radial growth of any fairly well developed spherulites that are already present. The region below *T_c_* is called region **B′**. If it were not for the nonsteady state nucleation effect, Kegion **B′** would exhibit a (Δ*T*)^+1^ radial growth rate law analogous to eq (10). Alternatively, bundlelike nuclei of nonsteady state origin may appear at *T_c_* if *σ_e_* is sufficiently small for embryos of minimal size.

#### Summary

A schematic diagram of the growth rate behavior of this model is shown in figure 6c. Curves (i) and (ii) in region **B′** represent different degrees of interference with the radial growth rate of the spherulite caused by the rapid incursion of crystallites of nonsteady state origin into the surrounding medium. The vertical mark near *T_m_* indicates the restriction that will occur on chain folding when the step height approaches one half the molecular length if large chain ends are excluded from the crystal. In the case of small chain ends that can be accepted as defects in the crystal, this restriction will be relaxed, and chain folding can then occur nearer to *T_m_.*

In these plots, the negative temperature coefficient part of the curves near the melting point is due to the nucleation term, and the positive temperature part occurring at lower temperatures is due to the effect of the jump rate term.

Using normal values of the parameters in the nucleation term in eq (34b), and setting ΔH*=20,000 cal mole^−1^ (or alternatively, using eq (24) in its range of validity), the maximum in log **G_A′_** is found to appear somewhere between 0.8 and 0.9 *T_m_.* Since the glass transition temperature *T_g_* for many polymers obeys the empirical relation *T_g_*=0.66 *T_m_*, it is seen that the maximum in log **G_A′_** falls about midway between *T_m_* and *T_g_. T_c_* will often occur well below the maximum in log **g_A_**_′_, say at 0.75 *T_m_.* It follows that the *T_c_* transition will frequently be obscured by the lowering of log **G_A′_** by the jump rate term. Thus it is to be expected that only a simple maximum of log **G_A′_** versus *T* will appear in many instances, as denoted by the solid line in figure 6c. The *T_c_* transition would be most easily found in a polymer with low ***σ*** and high **a**_min_, the transition being closest to *T_m_* in this case.

It can be shown for a number of systems that an excellent fit of the radial growth rate of the spherulites as a function of temperature can be obtained with an expression of the form of eq (34b), i.e., og*_e_*(*G*/*G*_0_) = −ΔH*/*RT*−*K*_1_/*T*^2^(Δ*T*), where *K*_1_ is a constant. The value of *K*_1_ can be used to determine the product ***σσ****_e_*, or more precisely ***σ***(***σ****_e_*+ **ϵ**/**b**_0_), for a chain folded lamellar spherulite. This product should frequently lie in the range 100 to 1,000 erg^2^ cm^−4^. Such values are obtained, but it is not certain that they all refer to distinctly lamellar splierulites.

### 5.2. Behavior of the Step Height

The step height of a growing lamella, l*, is given by eq (32). This expression holds no matter whether the lamella was originally heterogeneously or homogeneously nucleated. The term 2***σ****_e_*/(Δ*f*) will contribute at least one half of the full value of l*, and possibly almost all of it. The contribution from the *kT*/**b**_0_***σ*** term is rather small, and nearly constant, as indicated previously. The other term, 2**ϵ**/**b**_0_(Δ*f*), will now be considered, especially in relation to how it affects the step height of the growth nucleus compared to the primary one.

The step height of a homogeneous nucleus with folds is 4***σ****_e_*/(Δ*f*).^18^ If ***ϵ*** is negligible, the step height of the coherently grown lamella will be only slightly over one half of this value, namely, 2***σ****_e_*/(Δ*f*)+*kT*/**b**_0_***σ***. As the effect of ***ϵ*** increases, the step height of the growing lamella will eventually approach the value 4***σ****_e_*/(Δ*f*). The step height of the growth nucleus will not exceed that of the primary nucleus because ***ϵ*** is a function of position, and will fall to a low or zero value outside the edge of the primary nucleus. (It will be recalled that ***ϵ*** is a measure of any extra work that might be required to cause a loop to lie on a flat substrate in from the edge or at the edge; it is therefore either zero or quite small for a loop protruding over the edge.)

The main point here is that it is not always to be expected that a growing lamella will have the same step height as the primary nucleus with folds.

#### Temperature Dependence of Step Height

The temperature dependence of the step height is of considerable interest. It is seen from eqs (7) and (32) that the step height of a growing lamella is

$$l*=\frac{(2\sigma_{e}+2\epsilon /b_{0})T_{m}^{2}}{(\Delta h_{f})(\Delta T)T}+\frac{kT}{b_{0}\sigma }.$$ (35)

Ignoring relatively unimportant variations with temperature, this may be written in the approximate form

$$l*=\frac{Y}{\left(\Delta T\right)}+Z.$$ (36)

Thus, l* will increase with rising temperature, reaching large values near *T_m_.* In most experiments, the quantity **Z** *= kT*/**b**_0_***σ*** will be negligible compared to **Y**/(AT). (The value of **Z** will commonly be roughly 20 A.)

It should prove possible in some cases to verify the temperature dependence of the step height predicted by eq (36) by careful low-angle X-ray studies on unoriented bulk polymer specimens crystallized at various temperatures. If the X-ray spacings indicate such a dependence of step height with degree of supercooling, this may be regarded as a confirmation of the kinetic nucleation viewpoint of chain folded growth proposed in this paper. This confirmation would be especially convincing in the case of a polymer that was known from electron microscope studies to have a lamellar texture, since this in itself strongly implies the existence of substantially chain folded crystals. It is very doubtful that strictly bundlelike crystallization could lead to eq (36), because such crystals would either grow in the **l** direction (noncumulative strain) or abort (cumulative strain). However, a demonstration that eq (36) is obeyed may still only mean that a substantial fraction of the crystallization present is in the chain folded configuration, rather than all of it; some bundlelike character is to be expected in the otherwise chain folded lamellae, and numerous small bundlelike embryos may be present. (See also remarks in section 8.3 concerning crystallization from the highly oriented melt.)

An interesting point that arises in connection with the above is that the theory predicts that the characteristic step height of the lamellae in a spherulite can be alternated between different values by successively crystallizing at different temperatures. In this way it should be possible to make lamellae that are alternately thick and thin as the radial growth of a spherulite proceeds.

It is emphasized that the results quoted above refer to *isothermal* crystallization. For slowly growing spherulites, sufficiently isothermal conditions will be maintained, but at strong supercooling where the rate of crystallization becomes high, the heat of crystallization may raise the local temperature and tend to artificially flatten out the l* *versus T* curve predicted by eq (35) or (36).

#### Melting Behavior

Consider now the melting point of the chain folded lamellae. It is readily deduced from eq (30) that the temperature of melting of a lamella that is large in the **a** and **b** dimensions is given by

$$T_{m}^{\prime }\;(l)=T_{m}\;\left[1-\frac{2\sigma_{e}}{(\Delta h_{f})l}\right]$$ (37a)

where **l** is the step height of the crystal [3]. It is seen that the equilibrium melting temperature, *T_m_*, is the melting point of a lamella of infinite step height. From this expression and eq (32) with ***ϵ***=0, it is readily found that a lamellae of step height l* formed at a crystallization temperature *T_x_* will melt only slightly above *T_x_* on rewarming if the edge free energy is unimportant. If on the other hand ***ϵ*** is large, so that the step height of the lamellae is the same as that of a primary nucleus, 4***σ***_e_/(Δ*f*), then its melting point *T*′*_m_* will be just midway between *T_x_* and *T_m_* i.e., *T*′*_m_*=(*T_m_+T_x_*)*/*2.

The melting point of a polymer is commonly understood to refer to the temperature where the last detectible trace of crystallinity disappears. Lamellae will ordinarily exhibit a certain distribution of step heights, and the “observed” melting point, *T*′*_m_* (obs.), will thus refer to the larger of these. We must expect the step height at the observed melting point to reflect the mean step height of the growth or primary nucleus, whose **l** value varies as 1/(Δ*T*). Suppose that the observed melting point corresponds to a step height ***β*** times larger (or smaller) than the mean step height of a primary nucleus. Then $l=\beta l_{p}^{*}≅4\sigma_{e}\beta T_{m}/(\Delta h_{f})(\Delta T)$, where ***β*** is to a first approximation assumed to be constant. For this simple illustrative example one finds

$$T_{m}^{\prime }\;(\mathrm{obs}.)=T_{m}\;\left(1-\frac{1}{2\beta }\right)-\frac{T_{x}}{2\beta }.$$ (37b)

This expression indicates that a plot of the observed melting point (as obtained on warming without recrystallization) as a function of crystallization temperature will be a straight line that intersects the line $T_{x}=T_{m}^{\prime }\left(\mathrm{obs}.\right)$ at *T_m_.* This may prove useful in determining the equilibrium melting temperature. However, the main points here are that the observed melting point will tend to increase as the crystallization temperature is increased, and that it is entirely possible that such melting points may either all be above the line $T_{m}^{\prime }\;(\mathrm{obs}.)\;=(T_{m}+\;T_{x})/2$, as for ***β***>1, or all below as for ***β***<1. If the edge free energy is large, and the distribution fairly broad, ***β*** may be in the vicinity of 1.5 or slightly more. On the other hand, if the edge free energy is negligible and the distribution narrow, ***β*** will tend to fall in the range 0.6 to perhaps 1.0. Experiments showing that ***β*** was in the range 0.6 to say 1.5 or 2.0 would provide strong evidence for the retention of the step height of the crystal near that of the nucleus length.

The above results apply to the case where the lamellae maintain their original step heights as they are rewarmed. Small lamellae will melt out well below *T_m_*, and any subsequent recrystallization at this higher temperature would form higher-melting lamellae (see below). Also, a certain increase in step height may sometimes arise from lengthwise diffusion effects in the chain folded crystal. Either way, ***β*** will increase, and $T_{m}^{\prime }\;\;(\mathrm{obs}.)$ will be correspondingly closer to *T_m_.* Values of ***β*** as high as 5 or so may still be regarded as being consistent with chain folded growth, but the most clear cut case is that where ***β*** lies in the range of about 0.6 to roughly 1.5 or 2.0.

Some recent experiments indicate that a satisfactory explanation of the melting of certain bulk polymers of the lamellar type is given by eq (37b) with reasonable ***β*** values [44].

Combination of eqs (37a) and (38) shows that the melting process will be sharper for an assembly of thick lamellae formed near *T_m_* than the thinner ones produced at lower temperatures. To this approximation, the breadth of the melting process is proportional to (Δ*T*). This result will be modified somewhat when the distribution of step heights within a given lamella is considered.

#### Recrystallization on Warming

Recrystallization may occur on rewarming. The thinner lamellae formed in the original crystallization at *T_x_*_(1)_ will melt out at the $T_{m}^{\prime }$ value characteristic of their step height as described above. Then lamellar crystals with the larger step height characteristic of the crystallization temperature $T_{x(2)}=T_{m}^{\prime }$ will tend to form from the melted material. Thus, on warming from *T_x_*_(1)_ to *T_x_*_(2)_, *a small step height will tend to be replaced by a larger one.* This effect should not be confused with the slow and monotonic increase of step height due to lengthwise diffusion of the chains in the crystal that may sometimes take place. The latter process, if it occurs, should be identifiable, since it will lead to a slow and isothermal increase of step height at the original crystallization temperature.^19^

Since the step height increases on recrystallization, the new lamellae formed in this process will be smaller in the **a** and **b** directions than the patch originally melted out. The recrystallization process may be promoted by the presence of loop type crystals in the immediate vicinity. Something rather like what has been described may occur for a lamella on a heterogeneous surface, but here the problem is more complicated because of the interaction with the foreign substrate.

Growth of the step height to increasingly higher values by recrystallizing at successively higher temperatures through slow warming cannot be carried out indefinitely, since kinetic considerations will at some temperature near the melting point prevent such recrystallization from taking place at a sensible rate. The large step height achieved by recrystallization at the highest practical temperature is *not* to be regarded as a “limiting” step height from a theoretical point of view.

#### Distribution of Step Heights

We turn now to the question of what the model implies about the distribution of step heights of various lamellae. To a good approximation, it has been shown that the mean square deviation of the step heights of different lamellae about the mean value $\bar{l}$ is given by [3]

$${\langle \left(l-\bar{l}\right)\rangle }^{2}=\frac{1}{2}{\left(\frac{kT}{b_{0}\sigma }\right)}^{2}.$$ (38)

For ***σ***=10 erg cm^−2^, *T*= 400 °K, and **b**_0_=5×10^−8^ cm, the quantity ${\langle \left(l-\bar{l}\right)\rangle }^{2}$ comes to 60 A, corresponding to a mean deviation about $\bar{l}$ of only 7.8 A. Thus the mean step height of a given lamella formed in the coherent growth process is apt to be quite close to the average for the entire assembly of lamellae.

If the edge free energy is greater than zero on the face of the crystal, and falls to a lower value at the edge, then the distribution of step heights of different lamellae will be even less than that given by eq (38). The case described amounts to the physical situation where a loop is a little easier to form at the edge rather than upon the flat face of the lamella.

The treatment leading to eq (38) for surface nucleation with chain folds is quite different from the one that establishes the distribution of step heights for primary folded nuclei. The difference is that the distribution of step heights for various folded primary nuclei about $l_{p}^{*}$ may be properly treated in terms of a continuum model of the free energy surface (see p. 83 and appendix 5.1 of reference [3]),^10^ while in the case of the folded surface nucleus of monomolecular thickness the discrete character of the free energy surface must be recognized in the calculation of the distribution of step heights about l* (see pp. 86–87 and appendix 5.2 of reference [3]). However, the narrow range of values of the step height at a certain growth temperature is in both cases a result of the fact that the rate of formation of the appropriate nucleus is at a sharp maximum for some value of the step height, i.e., at l* or $l_{p}^{*}$.

The question of the constancy of the step height within a given coherently growing lamella subsequent to the initial surface nucleation act has been treated in some detail by Lauritzen [29].^20^ Allowing the height of each step element to fluctuate occasionally about l*, and assuming that the polymer chain (because of its segmental character) can take on only discrete values of **l**, it was shown that the step height would maintain itself slightly above the value **l**=2***σ****_e_/(Δf*) for the case ***ϵ***=0 from near *T_m_* down to temperatures corresponding to a rather high degree of supercooling. Though some minor differences exist, the more rudimentary theory given in this paper, where l* is assumed to remain constant after the initial surface nucleation act, is verified on the main points.^20^ Physically, the constancy of the step height of a given lamella during growth is a result of the fact that the step height cannot become smaller than 2***σ****_e_*/(Δ*f*) without becoming unstable, and does not tend to become substantially larger than this value because it is expensive from an energetic standpoint to allow a loop to protrude much out of the fold plane. This causes the lamella to have the maximum steady-state growth rate when its step height is l* (cf. sec. 5.1).

Insofar as the disorder of a folded crystal is a result of the distribution of step heights, i.e., irregularities in the fold plane, Lauritzen’s calculations indicate that crystals formed at low supercooling should be more ordered than those produced at high supercooling, where fluctuations are more pronounced.

### 5.3. Coherent Loop Type Growth and Spherulite Structure: Lamellar Twist

Growth by coherent nucleation of folded nuclei can lead to the formation of a spherical object that in most important respects strongly resembles a lamellar spherulite.

Consider first the case of a heterogeneously initiated spherulite. Here stacks of lamellae of the general type shown in figure 6a will grow outward from various sites on the nucleation center. (The reader is reminded again that the growing surface may actually be wedged-shaped.) The ***σ****_e_* planes of the lamellae (loop containing surfaces) will be more or less parallel to the spherulite radius, and the polymer chains will be essentially normal to the radius.

The spherulite will tend to fill out and become a three-dimensional object by virtue of one or more of several processes. First, new lamellae will tend to form and grow along the ***σ****_e_* faces of those already in existence. Second, spiral dislocations can occur. The axis of these dislocations, *s*−*s*′, will be at a right angle to the ***σ****_e_* plane, i.e., normal to the spherulite radius, and “radial” growth will occur from the edges of such dislocations. Certain types of branching are also possible as secondary nucleation events. For example, noncoherent nucleation may occur on the faces or edges of a lamella.

It is evident from the above that this model leads in a natural way to the lamellar and polymer chain orientation common in spherulites, and it is also clear that a three-dimensional spherical object can be formed. If the number of active heterogeneous nuclei is very large, easily recognizable spherulitse may not form because of early impingement, but the polymer texture will still be basically lamellar. This effect may be greatly reduced by ridding the system of the heterogeneities, which is difficult, or somewhat reduced by destroying some of the embryos in the fissures on the surface of the heterogeneities by heating the sample further above *T_m_* prior to crystallization (see sec. 8.4.).

Homogeneous nucleation can also lead to a lamellar spherulite. Here the nucleus will first grow a small platelet. Then by the mechanisms mentioned above, a three-dimensional object can form from it. Early in its life, the spherulite may be fan or sheaffike. (The same would be true of a heterogeneously nucleated spherulite if only a few nucleation sites on each heterogeneity were active.) The spiral dislocation mechanism is probably important in creating a three-dimensional spherulite, but the radial growth itself is not due to the formation of such spiral ramps. At sufficient supercooling, the number of homogeneous nuclei will become very large even early in the crystallization process, so that obvious spherulites may not appear despite the fact that the texture is actually lamellar.

An essential point to bear in mind in the case of ideal coherent growth with chain folds is that a number of the lamellae should extend outward from the nucleation center without interruption a substantial fraction of the way to the boundary of the spherulite. It should be possible to obtain information on this point by examining electron micrographs of a cross section of a spherulite. Evidence that a single lamella without any obvious disruption extended over a substantial fraction of the spherulite radius would be consistent with the coherent growth with chain folds mechanism, and at the same time give reason for anticipating a (Δ*T*)^−1^ radial growth rate law. Geil [5] has shown that individual lamellae extend over the entire radius in the spherulitic structures of bulk polyoxymethylene.

In the coherent growth process, chain ends will appear from time to time on the growing surface as the loops are laid down. Continuation of growth of the surface layer involved may be accomplished by allowing the incomplete step element to come off the crystal face and protrude like a cilia from the ***σ****_e_* face. This is certainly what will happen in high molecular weight material if the chain ends are sufficiently large. Renucleation with a new molecule at the sites where the other molecule terminated would be more rapid than the formation of a new coherent surface nucleus, so such a process would not materially alter the rate equations given. Small ends that can enter the crystal in sufficient numbers will allow relatively normal chain folding even for material of moderate molecular weight. Chain ends may play a role in initiating dislocations.

It has already been noted that the perfection of the folded surface will increase with increasing crystallization temperature. This will tend to cause more perfect crystals of higher density to be formed at higher temperatures. It might be thought that the inclusion of chain ends in the folded crystal might have a strong effect in the opposite direction. Actually, a simple calculation shows that over a wide range of step height, the number of chain ends included in the crystal per unit volume will not depend strongly on the step height, so increasing density with increasing crystallization temperature is commonly to be expected.^21^ The inclusion of a certain number of chain ends in the folded crystals of a high molecular weight polymer must be expected to slightly lower the melting point. Nevertheless, for polymer crystallized in the usual range of supercooling, the finite step height must be expected to be the main cause of the lowering of the melting point below *T*_m_, as described by eqs (37).

Some homopolymers may be practically completely lamellar when crystallized, but in other cases a considerate amount of amorphous matter may exist between the lamellae, especially if the lamellae are strongly twisted. The amorphous material between lamellae may be oriented, and abnormal in other respects.

The chain fold model of spherulitic growth leads in a straightforward way to the existence of terraces corresponding to the lamellar step height in surface replicas of fractured spherulites. In the case where the number of interlamellar links is low (see below), van der Waals interactions comprise the main forces that must be overcome to separate the lamellae. Steps of the type illustrated in figure 6(a), as viewed from a direction essentially normal to the ***σ****_e_* plane, are to be expected. This appears to correspond closely to what is often observed in electron micrographs of material crystallized from the unoriented melt. If the number of interlamellar links is high, cleavage of the lamellae may be quite difficult, and result in considerable damage to the step structure.

#### Interlamellar Links

A fairly large number of molecules may be incorporated bundle-fashion into a chain folded lamella growing in the bulk supercooled liquid, as illustrated in figure 6a by the molecule marked *y—y*′. This may be done to a certain extent without seriously affecting the step height of the folds or the rate expressions. Even the fairly frequent inclusion of such chains will not tend to incur the cumulative strain effects that may be associated with the formation of purely bundlelike structures. Also, groups of lamellae will tend to grow outward from the nucleation center together. The situation should resemble that shown in figure 6a, except that the growing faces of the lamellae will frequently be closer together. Under these circumstances, it is inevitable that one polymer molecule will occasionally become involved in two or perhaps even three different lamellae, creating interlamellar links. These links should affect the mechanical properties of spherulitic bulk polymers, since the lamellae will be more difficult to separate than would be the case if such links were absent.

Polymer molecules emanating bundle-fashion from the fold plane may sometimes return to the same crystal at a position well removed from the original point of exit. This type of “folding” may be denoted as “nonadjacent re-entry”, as opposed to the usual type of chain folding, which exhibits adjacent re-entry. However, it seems quite unlikely that the basic structure of a well-defined lamellar polymer crystal formed at low to moderate supercooling consists of folds that largely or entirely exhibit nonadjacent re-entry.

In unoriented bulk polymers crystallized under conditions where the number of interlamellar links is low, elongation of the specimens by cold stretching in one direction should cause the lamellae to tend to aline in the direction of stretch; in such a case, the polymer molecules in the crystals would tend to be oriented more or less *perpendicular* to the direction of stress. Such a result would provide strong support for the existence of lamellae with chain folds. The existence of too many interlamellar links, local melting caused by rapid stress, or excessive elongation, could easily lead to the opposite result. (It should be understood that the above refers to polymer crystallized from the unoriented and relatively unstrained supercooled liquid state; experiments carried out on crystallization of highly oriented liquids, e.g., cooling of hot-drawn filaments, are specifically exempted.) Examples where the tipping of crystals at low draw ratios has been observed may be found in the literature [30, 31, 32, 33]. At least some of these studies appear to refer to the required type of experiment. Results of this kind led Storks [34] to originally suggest the existence of chain folds.

The existence of interlamellar links in polymer crystallized in bulk may cause such material to differ from masses of chain folded polymer platelets filtered from dilute solution preparations. In the latter case, interlamellar links should be virtually absent. If numerous, the interlamellar links in the bulk crystallized polymer might cause disturbances in the solubility, mechanical properties, and melting behavior.

#### Lamellar Twist

**Figure 7:**
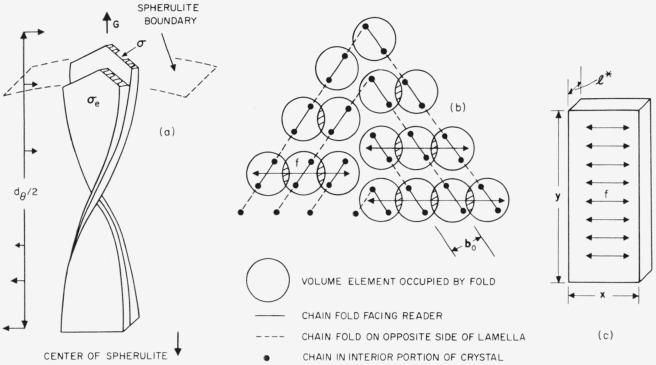
Origin of lamellar twist in spherulites. (a) Schematic representation of lamellar twist. (b) Schematic view normal to fold plane of lamellar crystal showing repulsion (shaded areas) leading to surface stress *f*. (c) Macroscopic model of surface stress. A similar stress pattern exists on the opposite side of the body.

The remaining question is that of the origin of twist of the lamellae. The optical studies noted earlier indicate that the lamellae (or more correctly stacks of lamellae) in many real spherulites are twisted, and it remains to be seen if coherently grown lamellae with chain folds could exhibit such an effect. The type of twist under consideration here is shown schematically in figure 7a. The bladelike lamellae have a definite pitch, and revert from time to time to their original orientation at a repeat distance **d***_θ_* as one proceeds along the radius of the spherulite. The heavy arrow marked **“G”** indicates the direction of radial growth. The leading face of the lamella has been drawn wedgeshaped to correspond to growth in a preferred crystallographic plane (see also fig. 7b).

The twist of the lamellae can arise from stress in the plane of the chain folds. One way in which the required type of stress can arise will now be outlined.

Let the polymer chains in the interior of a lamella be arranged in some definite pattern, say the hexagonal array shown as small heavy black dots in figure 7b. It is assumed that this internal packing arrangement does not lead to twist. Now consider the “lattice” of the chain folds that is consistent with this internal structure. If we consider the chain folds as occupying a roughly spherical volume element, the situation shown in figure 7b results. If the chain folds occupy more than a certain volume (denoted by large open circles) repulsive forces will act between the folds as indicated by the overlap (shaded). The main point to notice is that the “lattice” created by the chain folds on the two ***σ****_e_* surfaces is not the same as that characteristic of the interior of the crystal, and that if the folds are larger than a certain size, an anisotropic surface stress is certain to result. (A different packing of the chain folds can be introduced by alternating the chain folds in the layers of molecules in figure 7b, but this simply leads to straight rows of folds that lie at an angle of 60° to those shown.) The surface stress increases the free energy of the crystal, and the system will tend to undergo slight rearrangements that will minimize the total free energy.

There are two basic ways in which the lamella can reduce the repulsion of the chain folds. First, the lamella can twist slightly to create better surface packing (see below). This will, of course, tend to be balanced by forces due to the internal packing arrangement. Second, the chain folds may tend to become staggered, causing the folded surface to resemble a terra cotta roof. The staggered configuration corresponds to that where the polymer chain axes are no longer perpendicular to the plane of the chain folds.^22^ Specific forces in the interior of the lamella, related to the particular way in which the X-X-X groups that comprise the chain achieve best packing, may tend to resist such staggering. It is possible that the minimum in free energy will be achieved if both effects occur.

Now consider the problem of the origin of the twist in a body with surface stress of the type that may occur in polymer lamellae. The particular type of surface stress arising from the model shown in figure 7b may be illustrated schematically as in figure 7c. Here the solid body of dimensions **x**, **y**, and l* possesses a surface stress *f* in dynes cm^−2^ that is distributed over a surface layer of thickness *γ*l*, where *y* is a small fraction. The situation on the opposite side of the parallelepiped is identical to that just described.

From the theory of the buckling of plates,^23^ it can be shown that such an object will develop twist or warp when *f* exceeds a certain value which is

$$f_{c}=\frac{S}{2\gamma }{\left(\frac{l*}{x}\right)}^{2}.$$ (39)

Here *S* is the shear modulus. Numerical estimates for the extremely thin lamellae characteristic of spherulitic crystallization indicate that twist can arise for surprisingly low values of *f_c_*/*S* if l* is in the usual range for such crystals. It is therefore considered entirely reasonable to propose that surface repulsions of the type described can lead to the twist of the lamellae. Surface stress arising from other causes could also lead to a similar effect.

If for some value of l* the crystal is already twisted, the twist will tend to become more prominent if l* is reduced further. This assumes, of course, that **x** does not fall off as rapidly as l*. The variation of *γ* with l* is unimportant in most applications.

Perhaps the most important prediction that arises from the present conception of the origin of twist in coherently grown lamellae is that **d***_θ_* will tend to increase rapidly as the growth temperature is increased and at some temperature become extremely large. In practical terms this means that the concentric bands observed in many spherulites when viewed with crossed nicol prisms will become separated more and more as the growth temperature is increased, finally disappearing altogether. The lattice forces in the interior of a lamella tend to keep it in the untwisted state, while the surface stress promotes twist. Hence, if the step height l* increases as it does with an increase of growth temperature, the twist will rapidly become less pronounced at the same time.

When a certain value of l* is reached (or more precisely, a certain value of l*/**x**), the twist will disappear completely. Examples where the twist diminishes rapidly with increasing growth temperature are well known in lamellar spherulites. It is conceivable that the situation described above could be reversed in some cases because **x** increases more rapidly than l* with rising growth temperature.

Considering the proposed mechanism leading to twist, it would not be surprising to find that lamellae with a marked warp or twist were also characterized by the polymer chains being not exactly perpendicular to the plane of the chain folds.

A heterogeneously nucleated spherulite may have sectors with both right and left handed twist. At any given nucleation site on a heterogeneity, lamellae with a given sense of twist will be generated, and the sector that grows out from this site will tend to preserve this particular twist (see below). The quantity **d***_θ_* will be the same for both right and left handed sectors. If the polymer is heated above *T*_m_, embryos preserving the sense of twist may be retained in cracks or pores in a heterogeneity, with the result that a spherulite similar to the original one will frequently be regenerated at the same place on subcooling. A boundary will exist between right and left handed sectors. Since the probability of polymer chain connections between these sectors is small, spherulites should frequently fracture under shear at such boundaries.

If the lamellae growing out from a nucleation center are either densely packed, or grow out together as a stack, the sense of the twist in the sector under consideration will be preserved. (This is the “cooperative effect” of Keith and Padden [9].) However, if the individual lamellae are fibril-like and loosely packed, a spherulite where right- and left-handed lamellae are intertwined may appear. Such a spherulite would not exhibit distinct bands when viewed under crossed nicol prisms, despite the fact that the individual lamellae, or groups of lamellae, were actually twisted. Only the maltese cross effect would then be seen.

#### Summary

The foregoing development indicates that the coherent growth mechanism with chain folds can reproduce many of the structural features of lamellar spherulites. The existence of the lamellae themselves, and the behavior of the step height that characterizes them is explained. The orientation of the lamellae, and the orientation of the polymer chains in the lamellae, is in the correct relation with respect to the radius of the spherulite. Moreover, the twist frequently exhibited by stacks of the lamellae can be accommodated by the theory, thus removing one of the objections that had heretofore been leveled at coherent growth mechanism generally.

Our conclusion is that the coherent growth mechanisms with chain folds, with its (Δ*T*)^−1^ radial growth rate law, is worthy of serious consideration in the analysis of the behavior of lamellar spherulites.

Consideration will now be given to the problem of spherulitic growth by noncoherent surface nucleation with chain folds.

## ★ 6. Radial Spherulitic Growth by Noncoherent Surface Nucleation With Chain Folds

### 6.1. Basis of Model]

It is conceivable that coherent growth of the kind described in the previous section will be hampered by certain effects so that such growth practically ceases after a time. For example, continued coherent growth may in some cases lead to *cumulative* surface strain on the ***σ****_e_* faces because of the existence of chain folds that are slightly too large. Then after coherent growth has proceeded for a time, the end surface free energy will take on a value ***σ****_e_*_(_*_s_*_)_ that is larger than ***σ****_e_*, which is sufficient to practically completely arrest coherent growth. The value of ***σ*** on the coherently growing face will probably also take on a higher value which may be denoted ***σ***_(_*_s_*_)_. The subscript (*s*) denotes strain.

The problem that will be studied in this section is whether or not it is possible that radial growth can be reactivated in such a case by the formation of a *noncoherent* surface nucleus on the presumably still wettable face of the stunted lamella. The rate laws for such growth will be derived, and the conditions under which the formation of a noncoherent surface nucleus will be the rate determining step in the radial growth of a spherulite determined. The effect of such nucleation on spherulitic structure will be mentioned.

The concept that noncoherent surface nucleation may be involved in spherulitic growth is due to Price [16]. He suggested that the cessation of coherent growth might result from chain entanglements. The assumption used here that the cessation of coherent growth results from cumulative strain seems justified, if only as an initial hypothesis, by the structural considerations mentioned above. The rate laws to be derived below should hold at least approximately for noncoherent surface nucleation that follows upon the cessation of coherent growth due to any cause.

**Figure 8:**
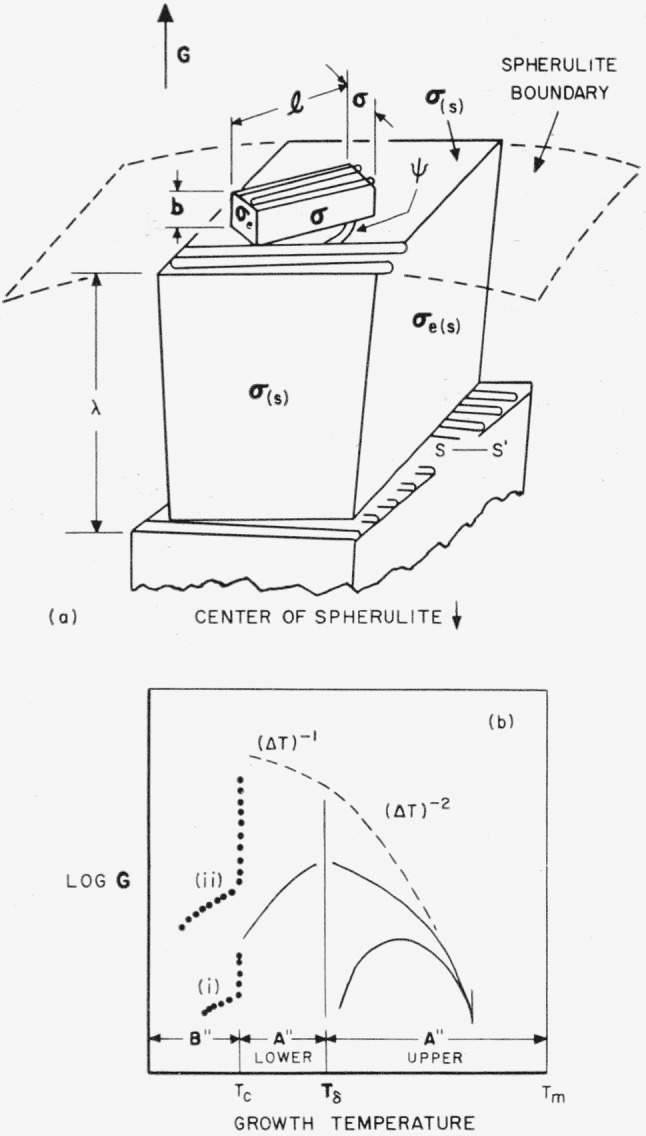
The noncoherent chain folded surface nucleus model of spherulitic growth and its rate behavior (a) Noncoherent surface nucleus of dimensions **a**, **b**, and **l** on strained substrate lamella. Heavy arrow marked **G** shows direction of radial growth of modified lamellar spherulite. (b) Logarithm of radial growth rate of spherulite as a function of temperature. ––––log **G** versus **T**, if jump rate effect is small; ——most representative cases where jump rate causes maximum to appear in log **G**;…. exhibits effect of excess nucleation in medium surrounding spherulite.

The model shown in figure 8a is used. A noncoherent surface nucleus with chain folds in the form of a parallelepiped of dimensions **a**, **b**, and **1** is assumed to form the face of the stunted lamella The noncoherent nucleus has normal “unstrained” values of ***σ***, ***σ****_e_,* and Δ*h_f_.* The substrate lamella bears the strained values of the surface free energies ***σ***_(_*_s_*_)_ and ***σ****_e_*_(_*_s_*_)_, but the volume contribution, Δ*h_f_*, has its normal value. The noncoherent surface nucleus attaches to the substrate lamellar face at an angle, *ψ* or 2*π* — *ψ*, that is consistent with minimizing strain. Thus, there is a true interface between the surface nucleus and the strained substrate crystal. This means that the noncoherent surface nucleus will behave in a three-dimensional manner, provided that neither **a**, **b**, nor **1** falls to minimal dimensions. Observe that the term “noncoherent” does *not* mean that the surface nucleus is not attached to the substrate.

Once a noncoherent surface nucleus of stable size is attained, coherent growth begins again, and the spherulite radius at first grows rapidly in the direction indicated by the heavy arrow marked **“G”**. Then the surface strain gradually accumulates, and the lamella is stunted after growing a distance **λ**. After a time, a noncoherent surface nucleus forms, and the process is repeated. Under appropriate circumstances, the formation of the three-dimensional noncoherent surface nucleus may be the rate determining step in the radial growth process.

### 6.2. Rate of Radial Growth With Chain Folded Noncoherent Surface Nuclei

#### Region **A″** (upper)

Assuming that **a**, **b**, and **l** may be regarded as variables in the sense that they have not reached minimal values, the free energy of formation of the unstrained noncoherent surface nucleus illustrated in figure 8a may be written

$$\Delta \phi =2\mathrm{ab}\sigma_{e}+2\mathrm{bl}\sigma +\left(2\sigma -\delta \right)\mathrm{al}-\mathrm{abl}\Delta f$$ (40)

where ***δ*** is defined by the relation

$$\sigma_{\text{interface}}=\sigma_{\left(s\right)}-\delta +\sigma .$$ (41)

Here ***σ***_interface_ is the total interfacial free energy between the surface nucleus and the lamella to which it is attached; ***σ***_(_*_s_*_)_ is the surface free energy on the face of the strained lamella. The other quantities have the same significance as in section 5. The edge free energy is omitted for simplicity.

The quantity ***σ*** is a measure of the interaction of the noncoherent surface nucleus with the strained substrate lamella. It takes on a value of zero if there is no interaction between the two objects. In this case **Δϕ** is identical to that for a primary nucleus with chain folds. On the other hand, if ***σ***_(_*_s_*_)_→***σ*** and ***δ***→2***σ***, eq (40) becomes identical to the expression for strictly coherent surface nucleation (*cf.* eq (30) with ***ϵ***→0). Thus ***δ*** will in general have a value between zero and 2***σ***.

A large value of ***δ*** thus indicates strong interaction of the noncoherent surface nucleus with the substrate (high degree of wettability), and a small value of ***δ*** indicates weak interaction (low wettability).

Near the melting point, **a, b**, and **l** may be regarded as variables, and it is therefore proper to calculate Δϕ* on the basis that the nucleus is formed from many step elements. Using procedures of the type outlined in sections 2.3 and 2.5, it is found that

$$l*=4\sigma_{e}/\Delta f,$$ (42a)

a*=4σ/Δf, (42b)

and

b*=(4σ−2δ)/Δf, (42c)

so the free energy of formation at the saddle point in the free energy surface described by eq (40) is at

$$\Delta \phi *=\frac{32\sigma (\sigma -\delta /2)\sigma_{e}}{{\left(\Delta f\right)}^{2}}.$$ (43)

Thus from eq (21) the radial growth rate would be^24^

$$G_{A^{\prime \prime }(\text{upper})}=G_{0}\;\exp\;\left(-\frac{\Delta H^{*}}{kT}\right)\;\exp\;\left(-\frac{32\sigma (\sigma -\delta /2)\sigma_{e}T_{m}^{4}}{T^{2}{\left(\Delta h_{f}\right)}^{2}{\left(\Delta T\right)}^{2}kT}\right)\;\left[{\left(\Delta T\right)}^{-2}\;\text{law}\right]$$ (44)

provided that (1) the quantity b* had not fallen to **b_0_** because of too large supercooling and (2) noncoherent surface nucleation was actually the rate determining step.

Let us now consider the range of validity of eq (44). First, it is readily found from eq (42c) that the nucleus will maintain its three-dimensional character only if the degree of supercooling is less than

$$\Delta T_{\delta }≅\frac{4(\sigma -\delta /2)T_{m}}{(\Delta h_{f})b_{0}}.$$ (45)

At temperatures lower than **T***_δ_*, the nucleus is monomolecular, i.e., **b=b_0_**, and the (Δ*T*)^−2^ law does not apply. Further, the condition

$$\;\exp\;\left(-\frac{4b_{0}\sigma \sigma_{e}}{(\Delta f)kT}\right)>>\;\exp\;\left(\frac{-32\sigma (\sigma -\delta /2)\sigma_{e}}{{\left(\Delta f\right)}^{2}kT}\right)>>\;\exp\;\left(-\frac{4b_{0}\sigma_{\left(s\right)}\sigma_{e(s)}}{\left(\Delta f\right)kT}\right)$$ (46)

must bold. The left hand inequality simply states that “unstrained” coherent nucleation must be more rapid than noncoherent nucleation. This will always be true when the degree of supercooling does not exceed **ΔT***_δ_*, and need not be considered further. The right hand inequality states that noncoherent nucleation must be more rapid than “strained” coherent growth, so that noncoherent nucleation will be the rate determining step in the radial growth process.^25^ This will be true when

$$\frac{\sigma_{\left(s\right)}\sigma_{e(s)}}{\sigma \sigma_{e}}>\frac{2(\Delta T_{\delta })}{\left(\Delta T\right)},$$ (47)

a condition that will hold according to the assumptions used in the model.

#### Region **A″** (lower)

At a degree of supercooling equal to or exceeding that given in eq (45), the noncoherent nucleus becomes monomolecular. Here the free energy of formation at **a** = **a**_0_ is

$$\Delta \phi^{*(1)}=\frac{4b_{0}^{2}\sigma (\sigma_{e}+\epsilon /b_{0})}{b_{0}(\Delta f)-(2\sigma -\delta )}$$ (48)

when the edge free energy is included. Somewhat below **T***_δ_***, b**_0_ (Δ*f*) will be considerably larger than (2***σ***−***δ***), and the radial growth rate may be approximated as

$$G_{A^{\prime \prime }(\text{lower})}≅G_{0(1)}\;\exp\;\left(-\frac{\Delta H^{*}}{kT}\right)\;\exp\;\left(-\frac{4b_{0}\sigma (\sigma_{e}+\epsilon /b_{0})T_{m}^{2}}{T\left(\Delta h_{f}\right)\left(\Delta T\right)kT}\right),\left[{\left(\Delta T\right)}^{-1}\;\text{law}\right]$$ (49)

which is identical to the radial growth rate for coherent loop type growth in region **A′**.

#### Region **B″**

The rate law described by eq (49) will hold down to temperatures corresponding to a supercooling Δ*T_c_*≅4***σ****T_m_*/(Δh*_f_*)**a**_min_. There and below, the rate of injection of nuclei in the surrounding medium will increase as described under Region **B′** of section 5.2.

#### Summary

A schematic diagram of the growth rate behavior of this model is shown in figure 8b. Even a fairly large value of ***δ***, say in the vicinity of 3***σ***/2, will still give a (Δ*T*)^−2^ radial growth rate region that extends from near the melting point on down to growth temperatures corresponding to a rather high degree of supercooling. For such values of **δ**, ***σ***_(_*_s_*_)_***σ****_e_*_(_*_s_*_)_ need be only several times larger than ***σσ****_e_* at ordinary supercooling to satisfy eq (47). However, the possibility exists that a rate transition from a (Δ*T*)^−2^ to a (Δ*T*)^−1^ law may occur at **T***_δ_*, as noted in figure 8b. This transition will not be particularly abrupt, and in the case where Δ**T***_δ_* is large, the rate laws will be mixed to the point that they cannot be clearly differentiated.

### 6.3. Noncoherent Loop Type Growth and Spherulite Structure

In broad aspect, a spherulite built up with chain folds by the process (rapid coherent growth→arrested “strained” coherent growth →noncoherent nucleation) will resemble one built on the coherent loop pattern. The spherulite will be basically lamellar, the chain axes will be normal to the radius, and the plane of the chain folds will tend to lie along the spherulite radius. Also, the coherently grown part of the lamellae will behave in a manner similar to that described in the previous section. However, there will be certain differences that may distinguish coherently grown from noncoherently grown loop type spherulites.

During the growth process, the lamellae will be disoriented with respect to the substrate at an angle of *ψ* or 2*π-ψ* at intervals of **λ**. For a completely isolated lamella, the noncoherent nucleus will attach to the substrate with equal probability at *ψ* or **2π**-*ψ*, so that such a lamella might show disturbances in its sense of twist at intervals of **λ**. Also, a distribution of *ψ* values may exist.

Perhaps the most unusual feature of spherulitic growth on this pattern that may become observable in an optical microscope is that if **λ** becomes sufficiently large, spasmodic radial growth may be observed. Each rapid increase in radial growth would correspond to the coherent growth that follows upon the formation of a noncoherent nucleus. The distance traveled in each pulse would correspond to **λ**. If sufficiently small, the repeat distance **λ** may lead to characteristic X-ray spacings, but these would probably be very difficult to identify or observe.

Since ***σ****_e_* increases because of cumulative strain as coherent growth proceeds, the characteristic step height l* will tend to increase somewhat as the coherent growth step takes place. This is shown schematically in figure 8a. This effect would probably be observed only with difficulty.

The extension of the interface between the strained crystallites should provide possible sites for the initiation of spiral dislocations (see *s*−*s'* in figure 8a).

Another interesting point is that the accumulated strain will tend to cause the lamellae to melt out on rewarming just a little above the crystallization temperature. As noted earlier, coherently grown lamellae with chain folds will generally melt out well above the crystallization temperature (eq 37b). The predicted low melting of the lamellae with cumulative strain may prove useful in eliminating the possibility of noncoherent growth in specific cases where such behavior is known not to occur.

Noncoherent nucleation may contribute to the formation of dendritic structures even in the case where the rate determining step is strictly coherent. Thus, noncoherent nucleation on the *σ*-type faces of a coherently formed lamella (such as that facing the reader in figure 6a) could lead to fern- or treelike structures. Such effects probably assist spiral dislocations in forming three-dimensional semicrystalline spherulites in some instances.

The noncoherent loop type growth model is realistic enough to at least warrant testing spherulite growth rate data to see if they accord with a (Δ*T*)^−2^ law. This rate law is to be anticipated in lamellar spherulites that grow in a spasmodic manner near the melting point, since the coherent model with chain folds will not lead to such an effect.^26^

## 7. Polymer Crystal Growth by Noncoherent Bundlelike Surface Nucleation

### 7.1. Basis of Model

Suppose that large chain ends limit coherent bundlelike growth as described in section 4, so that only tiny crystallites can develop by this mechanism. We will now examine the possibility that noncoherent surface nucleation will enter and reinitiate growth, and thereby produce a large microcrystalline and spherical object.

**Figure 9:**
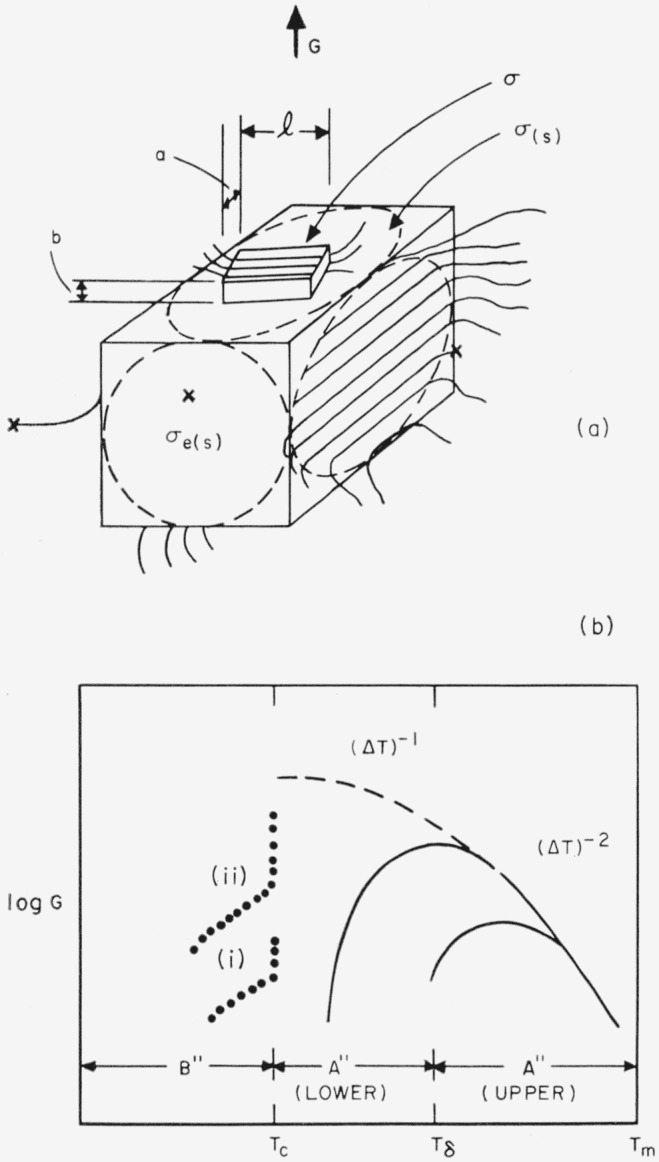
The noncoherent bundlelike surface nucleus model and its growth rate behavior. (a) Noncoherent surface nucleus of length *l*, width *a*, and thickness *b* on strained substrate crystal. Chain ends denoted—X. (b) Logarithm of radial growth rate as a function of temperature. The solid, dashed, and dotted lines have the same significance as in previous diagrams. (See text for assumptions required to obtain spherical object from this model.)

The model employed is shown schematically in figure 9a. The inhibition to continued coherent growth of the substrate crystal is a result of the physical obstruction presented by the chain ends—*x.* We assume that the presence of the chain ends also causes the surface free energies of the substrate crystal to have the “strained” values *σ*_(_*_s_*_)_ and *σ_e_*_(_*_s_*_)_ which are larger than the normal values *σ* and *σ_e_.* The latter refer to the surface free energies of relatively small bundlelike structures as yet unaffected by chain ends.

Alternatively, it could be assumed that cumulative strain resulting from growth in the *a* and *b* directions caused *σ* and especially *σ_e_* to increase.

It is considered that if a large spherical object is to be built at all on the bundlelike pattern, the present model is, at least in the beginning, a more reasonable one than the strictly coherent bundlelike model discussed in section 4, where the possible restrictions imposed on crystallite size by large chain ends or strain were arbitrarily neglected.

We will assume that a noncoherent surface nucleus in the form of a parallelepiped with the dimensions *a, b*, and *l* forms on the strained substrate crystal. This noncoherent bundlelike surface nucleus has normal values of *σ_e_* and *σ*. The noncoherent surface nucleus is assumed to form by virtue of the wettability of some portion of the substrate crystal by a normal crystal. Once it reaches critical size, this noncoherent surface nucleus will lead to coherent growth of a new crystallite of limited size. Then the process will be repeated. It is emphasized that the noncoherent surface nucleus is actually attached to the substrate, and that there is a true interface between the two objects.

### 7.2. Rate of Radial Growth With Noncoherent Bundlelike Surface Nuclei

#### Region A″ (*upper*)

Sufficiently near the melting point, *a*, *b*, and *l* may be regarded as unrestricted by any minimal molecular dimensions. The free energy of formation of the noncoherent bundlelike surface nucleus shown in figure 9a is

$$\Delta \phi =2ab\sigma_{e}+2bl\sigma +(2\sigma -\delta )al-abl(\Delta f)$$ (50)

where *δ* is defined in a manner analogous to eq (41). It is readily determined that

$$l*=4\sigma_{e}/(\Delta f)$$ (51a)

a*=4σ/(Δf) (51b)

b*=(4σ−2δ)/(Δf) (51c)

which on insertion in eq (50) leads to

$$\Delta \phi *=\frac{32\sigma (\sigma -\delta /2)\sigma_{e}}{{\left(\Delta f\right)}^{2}}\cdot$$ (52)

Then with eq (21) we have

$$G_{A^{\prime \prime }(\text{upper})}=G_{0}\;\exp\;\left(-\frac{\Delta H*}{kT}\right)\;\exp\;\left(-\frac{32\sigma (\sigma -\delta /2)\sigma_{e}T_{m}^{4}}{T^{2}{\left(\Delta h_{f}\right)}^{2}{\left(\Delta T\right)}^{2}kT}\right)[{\left(\Delta T\right)}^{-2}\;\text{law}]$$ (53)

if the formation of the noncoherent surface nucleus is indeed the rate determining step in the radial growth process. The quantity l* is *not* a step height in the case of a bundlelike nucleus, since the nucleus will grow to at least a certain extent in the *l* direction.

By comparison of eq (53) with the corresponding expression for homogeneous nucleation of bundlelike nuclei, eq (6) or (18), it is seen that the noncoherent surface nucleus model can provide a simple physical explanation for the reduction in the free energy of formation of a three-dimensional growth nucleus below that of the corresponding three-dimensional primary nucleus proposed by Flory and McIntyre [17]. The reduction factor is 1 − (*δ*/2*σ*), and this results from the assumption that an unstrained surface nucleus can wet a strained crystallite of the same polymer to some extent.

#### Region A″ (lower)

Equation (53) will hold on down to a temperature where *b* approaches *b*_0_. This will occur at a degree of supercooling

$$\Delta T_{\delta }≅\frac{(4\sigma -2\delta )T_{m}}{(\Delta h_{f})b_{0}}.$$ (54)

Below *T*_δ_, the rate law may be approximated as

$$G_{A^{\prime \prime }(\text{lower})}=G_{0}\;\exp\;\left(-\frac{\Delta H*}{kT}\right)\;\exp\;\left(-\frac{4b_{0}\sigma \sigma_{e}T_{m}^{2}}{T\left(\Delta h_{f}\right)\left(\Delta T\right)kT}\right).[{\left(\Delta T\right)}^{-1}\;\text{law}]$$ (55)

#### Region B″

At a growth temperature corresponding to a degree of supercooling of *ΔT_c_*=4*σT_m_*/(*Δh_f_*) *a*_min_, bundlelike embryos of small size will be transported from the superheated state above *T_m_* into the supercooled state by nonsteady state nucleation where they will become nuclei of stable size. As in previous cases, this will lower the radial growth rate of any spherulites born at or near *t*=0.

#### Summary

A diagram of the radial growth rate behavior possible with this model is shown in figure 9b. A (Δ*T*)^−2^ law will appear near and somewhat below the melting point because of the three-dimensional character of the noncoherent surface nucleus in that region. A transition to a (Δ*T*)^−1^ rate law may occur if the *δ* is fairly large. This transition will not be abrupt.

### 7.3. Noncoherent Bundlelike Growth and Spherulite Structure

There is nothing in the present model that suggests the existence of lamellae of large extent that have a uniform step height.

If large chain ends that cannot be assimilated into the crystal are taken to be the cause of the limitation on lengthwise growth of each of the individual crystallites in the coherent growth process, a large distribution of lengths, i.e., “lamellar thicknesses” would result. The mean length of the individual crystallites would depend on the molecular weight and its distribution, rather than on the degree of supercooling. With small chain ends that can occasionally enter the crystal, there is still no reason to suspect anything but a large distribution of such lengths, though the mean length would be larger and in any event not be of the correct magnitude to correspond to the thickness of a lamella. It is in our view extremely improbable that a collection of bodies of varying radii and length would aggregate in such a manner as to form slabs of uniform thickness with flat faces. We therefore conclude that the asumption that chain ends limit lengthwise growth is not consistent with the prediction of typical lamellae. Even if one considers bundlelike crystals before such a mean “equilibrium” length is attained, one must contend with the fact that large bundlelike crystals will tend to be ellipsoidal in order to minimize the total surface free energy and strain [25, 26], and the fact they will continue to grow in the *l* direction.

If cumulative strain at the bundle ends is taken as the cause of the cessation of coherent growth, the “length” of the crystals might be quite uniform. However, the existence of such strain would clearly tend to force the individual bundlelike crystallites to have curved end surfaces, which is inconsistent with lamellar structure. Here again the mean length of the ellipsoidal crystallites would not depend strongly on the degree of supercooling.

In both of the cases mentioned above, the introduction of the noncoherent nucleation step allows the continuation of radial growth, but in no way suggests that true lamellae could be produced from the decidedly irregular or ellipsoidal microcrystals produced in the coherent step.

No contradiction to the concept that bundlelike crystallization will not lead to typical lamellar structures arises when the noncoherent variations of the special models depicted in figures 5b or 5c are considered. If the model illustrated in 5b were actually capable of eliminating cumulative strain (which is by no means certain), such strain could not be the cause of the cessation of coherent growth. The “lamella” would grow in the *l* direction as noted in section 4.2 until chain ends stopped them, but then they would possess a distribution of lengths as noted previously. Alternatively, if cumulative strain were not relieved by the tilting, each crystallite would have curved ends, and therefore not be able to form a lamella. The latter objection also holds for the model shown in figure 5c if cumulative strain is not relieved by tilting. If such strain is eliminated by the tilt for 5c, one is forced to the assumption that unassimilable chain ends must be the ultimate limitation on coherent growth, but this has already been seen to be inconsistent with the existence of typical lamellae. Also, the other objections to 5c noted in section 4.3 still hold.

The bundlelike microcrystals will tend to melt close to the crystallization temperature because of the accumulated strain.

The bundlelike noncoherent growth model might lead to a more or less spherical and semicrystalline aggregate composed of a vast number of small crystallites of nonuniform size. (The introduction of the noncoherent step involves the assumption that suitable surfaces for such nucleation are formed by the individual coherently grown crystals.) If such an object is identified in some bulk polymer by electron microscopy or other methods, it would be reasonable to attempt to treat its radial growth with this model. However, it would seem more appropriate to treat a typical lamellar spherulite in terms of the coherent or noncoherent chain fold models.

If noncoherent nucleation is impossible because no suitable surface is presented by the coherently grown crystal (e.g., because of excessive curvature), small bundlelike crystallites may appear more or less at random in the system. These aborted structures might coexist with folded structures that were meanwhile growing to large size.

## ★ 8. Discussion

### 8.1. Synopsis of Radial Growth Rate Laws

The most important laws describing the rate of radial growth of spherulites derived in this paper may be summarized in the general form:

$$\log_{e}\;(G/G_{0})=-(\Delta H*/RT)-K_{1}/T^{2}(\Delta T)[{\left(\Delta T\right)}^{-1}\;\text{law}]$$ (56)

and

$$\log_{e}\;(G/G_{0})=-(\Delta H*/RT)-K_{2}/T^{3}{\left(\Delta T\right)}^{2}\cdot [{\left(\Delta T\right)}^{-2}\;\text{law}]$$ (57)

Another law was mentioned, but it is probably of little importance and is included mainly for the sake of completeness:

$$\log_{e}\;(G/G_{0})=[-(\Delta H**+\Delta H*)/RT]+K_{3}\left(\Delta T\right)\cdot [{\left(\Delta T\right)}^{+1}\;\text{law}]$$ (58)

A convenient summary of the rate laws as they arise in the various models is given in table 1 for coherent and noncoherent surface nuclei for both the bundlelike and chain folded classes. Given also is the best estimate of the type of “spherulite” that each model implies, and the sequence of rate transitions.

#### General Interpretation of Rate Laws

The (Δ*T*)^+1^ radial growth rate law holds when the rate determining surface nucleus has no dimensions that may be regarded as variables in the expression for the free energy of formation of the nucleus. The nucleus associated with this model, which is of the size *a*_min_ *b*_min_ *l*_min_, may therefore be described as *zero-dimensional*.

The (Δ*T*)^−1^ radial growth rate law bears the general meaning that the rate determining step involves a surface nucleus that has one fixed and two variable dimensions. (Such a body is commonly called a *two-dimensional* surface nucleus.) This meaning holds true for both the (Δ*T*)^−1^ law that appears beginning at the melting point in the coherent growth models, and the (Δ*T*)^−1^ law that arises at moderate or strong supercooling for the noncoherent growth models.

The (Δ*T*)^−2^ radial growth rate law arises when the rate determining step is the formation of a surface nucleus that has three dimensions that are to be regarded as variables in the expression that describes the free energy of formation ; such a body is commonly referred to as a *three-dimensional* surface nucleus. In the present conception, such a nucleus could be formed if the polymer molecules were deposited on the polymer substrate in such a manner that they were no longer colinear with those in the substrate, thus forming an interface between the two bodies. In this event, the surface nucleus takes on the three-dimensional character of a primary nucleus as regards temperature dependence, but is energetically preferred to the corresponding primary nucleus by virtue of the wettability of the substrate by the noncoherent surface nucleus. In our view, then, the observation of a (Δ*T*)^−2^ radial growth rate law would not only mean that the rate determining step was the formation of a three-dimensional surface nucleus, but would also imply that a noncoherent surface nucleus was involved.

#### Experimental Expectations

It is of interest to indicate what the theory implies concerning the probability that the various rate laws will be observed experimentally, together with a number of related points that may prove useful in attempting to apply the theory.

It is considered highly improbable that the (Δ*T*)^+1^ rate law will be frequently encountered in experimental studies. This rate law will generally be obscured or distorted by nonsteady state nucleation effects. If it did appear, it would do so at moderate to strong supercooling where the jump rate term would make it difficult to identify.

The (Δ*T*)^−1^ radial growth rate law would appear to deserve strong consideration in the analysis of data, especially in the case of obviously lamellar spherulites. We refer here specifically to the (Δ*T*)^−1^ law arising from the *coherent model with chain folds.* This model is capable of predicting many of the details of a lamellar spherulite, and coherent growth must be regarded as a probable mechanism, particularly if there is any reason to believe that a small and efficiently packed chain fold that is consistent with the interior lattice structure can be formed. On the grounds that studies on solution-grown single crystals appear to indicate that such folds can be formed in a number of cases, and the fact that lamellae of considerable extension have been observed in spherulitic bulk polymer samples, we believe that it is reasonable to suppose that a (Δ*T*)^−1^ radial growth rate law arising from coherent growth with chain folds should be found experimentally. Linear polymers with no bulky side groups or effective atactic sections are doubtless promising systems for such studies.^27^ The melting behavior of a number of spherulitic polymers seems more consistent with coherent growth than noncoherent growth.

The coherent bundlelike model also leads to a (Δ*T*)^−1^ growth rate law, but we do not consider this a likely source of such a rate law. It cannot produce a lamellar spherulite at all. Under a variety of conditions, the model does not even lead in a straightforward way to any kind of spherical object.

The (Δ*T*)^−2^ radial growth rate law deserves consideration in the analysis of data, most particularly in connection with the noncoherent chain fold model. It seems conceivable that in some polymers the chain folds may be too bulky to allow continued coherent growth, and in such cases it is possible that noncoherent three-dimensional surface nuclei may form, and constitute the rate determining step in the radial growth mechanism. (It is to be expected that such bulky chain folds would first induce a tilting of the polymer chain axes with respect to the ***σ****_e_* plane, lamellar twist, or even a change of crystal structure to a more open form.) If spasmodic growth of the spherulite radius is observed near the melting point, a (Δ*T*)^−2^ law becomes a good possibility.

The (Δ*T*)^−2^ law arising from the noncoherent bundlelike model need only be considered for nonlamellar spherulites.

It is evident that a combination of knowledge obtained from electron and optical microscopy and other appropriate physical methods concerning the texture of a spherulite, together with a knowledge of its radial growth rate law, would be very useful in deciding in detail how these objects were formed.^28^

### 8.2. Transitions in the Radial Growth Rate

The objective of this section is to bring out some points connected with the rate transitions that may occur in the radial growth rate of spherulites when the growth rate is considered as a function of temperature.

Two different types of rate transition have been postulated. In the first, the transition is due to some dimension of the surface nucleus approaching a minimal value, causing a different radial growth rate law to exist at lower temperatures (*transition in surface nuclei*). The second type of transition postulated in the radial growth rate is not due to any change in the surface growth nucleus itself; bundle or loop type nuclei where all three dimensions are of minimal size appear in large numbers as a result of nonsteady state nucleation in the surrounding medium, and interfere with the growth of well developed spherulites (*transition in surrounding medium*).

#### Transitions in Surface Nuclei

In the text, these are denoted **A**″ (upper) → **A**″ (lower) and A″ (upper) → A″ (lower). Such transitions occur only for noncoherent surface nuclei (see table 1). The transition is a result of the *b* dimension of the growth nucleus falling to its minimal value.

If it occurs, this type of transition will not be particularly abrupt. The theory for *G* in the transition region can be worked out for some simple models. The result is that *G* is actually continuous across the transition, provided that *G*_0_ and ΔH* are either constant with growth temperature, or smooth and continuous functions of temperature. It could happen that ΔH* or *G*_0_ was different in the two regions, so that the plot of log *G* against *T* near the transition resembled a branch point, or even exhibited a small discontinuity. In any event, it is emphasized that the rate laws cited in this paper hold at growth temperatures somewhat removed from the transition region.

The coherent folded nucleus model does not exhibit this type of transition, but the noncoherent folded nucleus model does. Therefore, if a rate transition involving a (Δ*T*)^−2^ law near the melting point and a (Δ*T*)^−1^ law beginning somewhat below it is found in a lamellar spherulite, the noncoherent model with chain folds would be indicated. A similar situation in a nonlamellar spherulite would point to the noncoherent bundlelike model.

In general, the appearance of transitions due to a dimension of a surface nucleus approaching a minimal value is not considered very likely, with the possible exception of the one associated with the noncoherent folded model. If found, such transitions would provide valuable insight into the spherulitic growth process.

#### Transition in Surrounding Medium (nonsteady state nucleation)

Consideration will now be given to the transition at *T_c_* resulting from the rapid ingress of loop (or bundlelike) nuclei in the medium surrounding a spherulite that may interfere with its growth.

It is to be expected that the *T_c_* transition can be relatively abrupt, occurring over a temperature range as little as several degrees. It will be accompanied by a noticeable fall in *G.* A transition fitting this general description has been found by Takayanagi [13] in poly (ethylene adipate) as may be seen by noting the lower transition in his figure 7. An even more remarkable drop in the radial growth rate of spherulites in poly (chlorotrifluoroethylene) at 156 °C, which is about 65 °C below *T_m_*, has been found by Hoffman and Weeks [35]. Another point of interest is that the rate of bulk crystallization, as measured dilatometrically, should increase at *T_c_*, even though *G* falls. This effect is particularly striking in the case of poly (chlorotrifluoroethylene).

The *T_c_* transition as such is certainly not to be expected in radial growth rate studies in all polymers. For many polymeric substances, ***σ*** will be around 10 erg cm^−2^, and **a**_min_ will be perhaps 10 A. Then the *T_c_* transition would appear only at a degree of supercooling of about 150 °C or more if (Δ*h_f_*) lies in the usual range. This will usually be below *T_g_*, where the radial growth rate has already been greatly lowered by the jump rate effect. Even if *T_c_* is nearer to the melting point than this, it is rather likely to be obscured by experimental difficulties arising from extremely rapid growth. The *T_c_* transition is most apt to be observed in materials where it is nearest to the melting point, i.e., those with a low lateral surface free energy and a large molecular diameter.

On the basis of the above remarks, the situation where the radial growth rate is low near *T_m_*, rises to a maximum below *T_m_*, and then falls again without any obvious discontinuities anywhere is the one to be most commonly expected. However, it is important to bear in mind the possibility that rate transitions can occur when analyzing radial growth rate data for polymeric systems.

Glass formation might be practically impossible, even with the most rapid quenching, if *T_c_* falls well above the glass transition temperature. Then the supercooled liquid (which would otherwise form a glass on cooling below *T_g_*) would tend to nucleate and crystallize rapidly at or somewhat below *T_c_.* At the very least, such a “glass” would contain a large number of “frozen in” embryos, nuclei, or crystallites in addition to any truly amorphous glassy material. The ordinary homogeneous mechanism (or a heterogeneous one) could, of course, cause crystallization to become very rapid above *T_c_.* In any event, the failure of many linear polymers to easily form truly amorphous glasses may involve the “nucleative collapse” effect beginning at *T_c_.*

The basic effect that causes the *T_c_* transition, namely, transport of a large number of nuclei of minimal size from the melt to the supercooled state, can be employed to explain certain effects associated with prequenching on spherulitic growth. In the case of the theory discussed so far, it has been assumed that we were dealing with specimens that were cooled directly from well above *T_m_* to the growth temperature, and the rate of radial growth observed under these conditions. Suppose, however, that experiments were carried out by first *quenching* the polymer to some low temperature, and then observing radial growth after the specimen is *rewarmed* to a certain growth temperature. Then at this growth temperature, *G* will often be lower than if the value of *G* had been obtained in the normal way at the same growth temperature. Results of this type are known, and may be ascribed to the incursion of small nuclei during the quenching process. The difference might be negligible if *T_c_* is near or below *T_g_.* Growth rate data obtained on prequenched and reheated specimens should be regarded with caution from an interpretive standpoint, since the retardations to radial growth will involve factors that have not been considered in deriving the expressions for *G*.^29^

### 8.3. Crystallization Without Chain Folds or Spherulites

The objective of this section is to emphasize the possibility that (1) crystallization without chain folds may occur, and (2) that crystallization with chain folds but without any obvious spherulites may exist.

#### Normal Bundlelike Crystallization

In the event that *σ_e_*<***σ****_e_* for both nuclei and crystals, and assuming that loop type structures are not initiated at heterogeneities, bundlelike crystallites might occur on a considerable scale. If noncoherent surface nucleation did not take place, the (probably ellipsoidal) crystallites formed would be scattered throughout the medium, i.e., no spherulites would form, but if noncoherent nucleation did take place, a spherical and microcrystalline but distinctly nonlamellar “spherulite” might form (see sections 4 and 7). The condition *σ_e_*>***σ****_e_* will lead to chain folded structures, but under certain conditions these may not be in a readily identifiable spherulite (see below).

#### Crystallization of Oriented Polymers

Suppose it is known for a certain polymer that when it is crystallized by cooling the unoriented melt to a temperature slightly below *T_m_* that lamellar spherulites are formed, and that the principal site of the crystallization is in the spherulites. Typical lamellar spherulitic crystallization frequently occurs under these conditions. We now ask what might change this state of affairs.

Assume now that the polymer liquid is in some manner caused to be in a highly oriented state above *T_m_*, and that this system is then supercooled. The possibility then exists that bundlelike rather than loop type crystals will form. In this event, the sample would contain neither lamellae or lamellar spherulites. A distinct long X-ray spacing may arise from such bundlelike crystallites if they have a fairly uniform size in the *l* direction due to strain or other factors. This spacing will almost certainly not vary as 1/(Δ*T*) as will the spacings from chain folded crystals formed from the unoriented melt, and care should be taken not to confuse the spacings obtained on strongly oriented and unoriented specimens. In moderately oriented systems, chain folded and bundlelike crystals may coexist.

#### Crystallization in Region B

Another possible source of nonspherulitic crystallization in a polymer whose macroscopically observable growth form at low to moderate supercooling is of the lamellar spherulitic type is rapid crystallization well below *T_c_*, i.e., in region B. This will often lead to a hierarchy of very tiny crystallites, most so small they scatter but little visible light. (A similar failure of well developed spherulites to appear may be caused by the presence of large numbers of active heterogeneous nuclei, or a very high rate of homogeneous nucleation.) It is reasonable to suppose that *σ_e_*<***σ****_e_* might hold for nuclei of minimal size even when *σ_e_*>***σ****_e_* for larger nuclei and crystals. This is implied by the concept that cumulative strain may exist at the bundle ends. In such a case, chain folded lamellar spherulites or other lamellar structures would form above *T_C_*, while many of the small crystals and embryos formed below *T_c_* would be bundlelike. The fine-grained crystallization produced below *T_c_* will tend to be mixed with spherulites formed above *T_c_* during the subcooling process.

In summary, the presence (or absence) of typical lamellar spherulites could depend on the previous orientation of the melt, the temperature of crystallization, the number of heterogeneities present, and the ratio *σ_e_*/***σ****_e_.* Conditions may exist where bundlelike and chain folded structures occur together.

### 8.4. The Initiation of Spherulites in Real Systems: Heterogeneous, Pseudohomogeneous, and Homogeneous Nucleation

One important reason for attempting to obtain the homogeneous injection rate of spherulites as a function of temperature lies in the fact that the temperature dependence of this quantity determines certain products involving the surface free energies that are different from those obtained from the radial growth rate. Thus, for a lamellar spherulite near *T_m_*, a knowledge of **I_A_** as a function of temperature would allow the product ***σ***^2^***σ****_e_* to be determined by eq (6) or (18). However, certain phenomena stemming from the presence of heterogeneities can closely imitate homogeneous injection, and lead to “***σ***^2^***σ****_e_*” values that are significantly low. These effects are discussed below, partly with the objective of indicating why the radial growth rate of spherulites, rather than their “homogeneous” injection rate, was stressed in the paper.

Consider first *heterogeneous* nucleation of spherulites. Turnbull [36] has shown that if a substance contains thermally stable (and wettable) heterogeneities containing pores or cavities on their surfaces, crystalline embryos can persist in these on an equilibrium basis well above the melting point. Such a body will act as a center of growth at or near *t*=0 after the material is supercooled. The number of such active embryos is strongly dependent on the temperature *T*_1_ above *T_m_* to which the system is initially heated if a crack-size distribution exists. By sufficient superheating, the embryos in the pores or cavities can be melted out, thus rendering them inactive as nucleation centers in a subsequent crystallization. Turnbull’s theory shows that, other things being equal, the embryos in the larger pores are melted out first as *T*_1_ is increased. In cases where the cavities are small, and where the heterogeneity is rather strongly wetted by the crystalline phase, the embryos may persist hundreds of degrees above the bulk melting point.

Spherulites in many cases are well known to follow this pattern of heterogeneous initiation. In such a case they are all born at or near *t*=0, and their number per unit volume is markedly dependent on *T*_1_—increasing *T*_1_ substantially reduces this number. (In cases where they are born later than *t*=0, they tend to be born in a narrow range of times about an induction time, *τ_i_.*) It is therefore clear that spherulite producing structures can be, and frequently are, maintained in cracks or fissures in heterogeneities.

We turn now to *pseudohomogeneous* nucleation of spherulites. Heterogeneities may contain flat but wettable regions in addition to pores or cracks. No embryos will persist on these flat surfaces above the bulk melting point. However, when a system containing such heterogeneities is supercooled, nuclei will preferentially appear on these flat surfaces by virtue of the wettability of these surfaces by the polymer crystal. If the number of heterogeneities with flat surfaces is large, the resulting crystals can appear in the supercooled system essentially sporadically in time instead of at *t*=0 or *t*=*τ_i_.* Under these conditions the crystals will appear not only sporadically in time, but also (on a macroscopic scale) randomly in space. For convenience we have denoted this as “pseudohomogeneous” nucleation. (This is the case of “heterogeneous” nucleation discussed by Avrami [37, 38].) Sporadic birth is, of course, also a property of crystallization in a truly homogeneous bulk phase.

Spherulites are sometimes seen to appear nearly sporadically in time and space in polymers. Pseudohomogeneous initiation should be suspected in any sample where the number of spherulites formed per unit volume in unit time for a given growth temperature depends on *T*_1_ or where a hierarchy of spherulites born at *t*=0 also appears together with those born sporadically. Such effects indicate the presence of numerous wettable heterogeneities. Some of the studies reported in the literature, where the polymer spherulites were believed to be of truly homogeneous origin for the reason that they appeared more or less sporadically in time and randomly in space, may actually refer to the pseudohomogeneous category.

*Homogeneous* nucleation refers to the process where crystallization centers are spontaneously formed at random positions in the pure (homophase) mother phase by thermal fluctuations. Such a process is characterized by a rate of production of nuclei per unit volume of mother phase that is, after the establishment of the steady state, truly constant in time. In short, the nuclei appear sporadically in time and randomly in space in the supercooled liquid. This process will be independent of *T*_1_ for a wide range of *T*_1_ values. True homogeneous nucleation of a bulk phase is an ideal situation not easily achieved experimentally in any bulk system, heterogeneous or pseudohomogeneous nucleation undoubtedly being much more common.

Spherulites of strictly homogeneous origin may appear in polymers, but a proof that this is true would be quite difficult. The following conditions are consistent with homogeneous initiation: (a) The spherulites appear sporadically in time with no excess population at or near *t*=0; (b) the spherulites appear at random positions in space, and show no memory of previous position in space in successive experiments;^30^ (c) further steps to rid the system of heterogeneities (e.g., filtration, centrifugation, or partial precipitation of solutions) do not alter the results; and (d) the rate of injection of spherulites is not dependent on *T*_1_.

It should be realized that true homogeneous nucleation has rarely been achieved or proved even with very carefully prepared specimens of ordinary size of *any* bulk material. One would hardly expect polymers to be an exception. Only work with logs [19] or dispersions [21], where the heterogeneous nuclei are greatly outnumbered by the number of particles with no adventitious centers, has heretofore been found effective in producing conditions where homogeneous nucleation predominated and could be identified. Accordingly, rate of spherulite injection data on bulk samples should not be treated using homogeneous nucleation theory unless there is substantial reason to believe that the injection mechanism was principally homogeneous. It seems probable that a number of the “*σ*^2^*σ_e_*” values quoted in the literature on the basis of the assumption of homogeneous nucleation in the bulk phase are too low.

It would be of great interest to obtain reliable ***σ***^2^***σ****_e_* values using dispersions of polymers in a manner analogous to that used by Turnbull for the *n*-paraffins [21].

### 8.5. Comment on Alternative Theory of Step Height

An alternative theory for the existence of a “step height” in polymer crystals has been advanced by Peterlin and Fischer [39]. They propose that the length (“step height”) of a polymer crystal is limited because the longitudinal lattice vibrations become incoherent, and raise the free energy of the crystal if its gets too long. The “step height” of lamellae is thus believed by these authors to exist because of equilibrium considerations. They predict that the step height decreases with lowering temperature.

Entirely apart from the question of whether or not such a concept is correct in principle, the following comments are relevant: (1) The Peterlin-Fischer theory does not predict, or even in the mode analysis take account of, the existence of chain folds in bulk or in dilute solution. (2) To the extent that their work may be interpreted as referring to the bundlelike system with chains normal to the bundle ends, the objection illustrated in figure 5a applies to the prediction of bundlelike “lamellae” of large dimensions. (3) Since the “step height” is a phenomenon based on equilibrium considerations in the Peterlin- Fischer theory, it should depend on the *ambıent* temperature rather than the *growth* temperature, the latter being the case for the theory presented in this paper. There is no evidence suggesting, for example, that the lamellae in bulk become thinner with lowering ambient temperature.^31^ It is our conclusion that if the limitation on length proposed by Peterlin and Fischer exists, it evidently refers to a much larger dimension than the step height of a lamella.

The theory presented here and in an earlier paper shows how chain folded crystals can come into being, and provides a reasonably detailed picture of the properties of systems crystallizing in this pattern.

## ★9. Summary and Conclusions

We now give a brief summary of some major points that have been brought out concerning spherulitic crystallization in bulk polymers.

It was demonstrated that if one assumes that the end surface free energy of a bundlelike nucleus is larger than the corresponding quantity for a folded nucleus, i.e., *σ_e_*>***σ****_e_*, then homogeneous nucleation of chain folded structures will prevail in bulk. It was noted that heterogeneous nucleation is much more probable in real polymer systems, but that if *σ_e_*>***σ****_e_*, this type of nucleation will in all likelihood still initiate chain folded structures in bulk. It was then shown that coherent surface nucleation with chain folds will lead to structures possessing a considerable number of physical features commonly associated with lamellar spherulites. (The chain folded mode of crystal growth is highly probable if *σ_e_*>***σ****_e_*, and coherent nucleation is feasible if the folds are such that they do not lead to cumulative strain in the fold plane.) It was also indicated that the assumption of noncoherent nucleation with chain folds could produce a modified lamellar spherulite. In each case, emphasis was placed on predicting the radial growth rate of the spherulite as a function of the crystallization temperature. This property follows a different law for coherent and noncoherent growth, and a differentiation of the two is amenable to careful experiment. Also, the behavior of the step height of a chain folded lamella in bulk was discussed. This included the step height as a function of growth temperature, the behavior on rewarming (melting, recrystallization), and the uniformity of the step height. The predictions given are mostly subject to experimental verification, and some are known to be at least qualitatively correct. It is highly significant that a kinetic theory of crystal growth, wherein a nucleus length maintains itself during growth because of chain folds, can reproduce so many of the known features of spherulitic crystallization in bulk.

Given the basic lamellar structure produced by the kinetic crystal growth theory with chain folds, it was shown how surface stress could cause the lamellae to warp or twist. A slight and noncumulative repulsion of the chain folds in the ***σ****_e_* plane is sufficient to create the required type of surface stress. Others have previously shown that such twist can explain many aspects of the optical extinction patterns of spherulites, most particularly the complex rings that are often seen with a polarizing microscope.

An effort was made to indicate the nature of the reasons that *σ_e_* might exceed ***σ****_e_.* It was concluded that the value of ***σ****_e_* will be considerably larger than had been supposed heretofore, because of the fact that a bundlelike nucleus clearly must possess a density gradient of considerable size at the bundle ends: calculations with a simple model were given showing that even the minimal value of ***σ****_e_* would be quite large because of the work required to construct this density gradient region. When considered together with the theoretical and experimental estimates for ***σ****_e_*, the result is that it is certainly not implausible to suppose that *σ_e_*>***σ****_e_*, at least in some situations. In the one specific case that was considered (polyethylene), it was clear enough that this condition might indeed apply. If *σ_e_*>***σ****_e_* for a polymer, chain folded growth is to be regarded as an intrinsic mode of crystallization in the bulk phase.

Certain circumstances were mentioned whereby chain folded structures might be prevalent, though in competition with numerous small bundlelike crystallites, in a bulk polymer even if *σ_e_*<***σ****_e_* (poisoning of growth of extended bundlelike structures by cumulative strain or large chain ends; heterogeneous nucleation with special interactions). In sufficiently dilute solution, chain folded platelets will form, no matter whether *σ_e_*>***σ****_e_* or *σ_e_*<***σ****_e_*, because of entropy considerations.

Assuming that the condition *σ_e_*>***σ****_e_* does exist, at least for fairly large crystals, we regard the most probable cause of this condition to be cumulative or noncumulative strain at the ends of the bundlelike nucleus or crystal. Such strain arises ultimately from the fact that for a bundlelike system, the crystal and liquid phases are “connected” through covalent bonds, a situation that does not occur to a significant extent in the folded system. Again we emphasize the fact that the condition *σ_e_*>***σ****_e_* virtually assures the predominance of the chain folded growth mechanism, whatever the type of initiation.

Considerable attention was directed toward a critical examination of whether the classical bundlelike model of polymer crystal growth, or certain variations of it, could lead to a lamellar spherulite. It was concluded that this was highly improbable.

In general, the bundlelike models suffered one or more of the following drawbacks: (1) The bundlelike nucleus with noncumulative strain is found to grow in the polymer chain direction, thus destroying any semblance of the stability or uniformity of the step height as observed experimentally in spherulites. (2) Bundlelike crystallites will have a tendency to exhibit rounded ends because of considerations based on strain or minimization of total surface free energy, or both, and this is not consistent with the existence of lamellae with large and flat *σ_e_*-type faces. The bundlelike nucleus with cumulative strain will not grow to large size, and will definitely have rounded ends. (3) The assumption that the exclusion of large chain ends from the crystal ultimately causes the cessation of lengthwise growth of the bundles (on an equilibrium basis) leads to a wide distribution of crystallite lengths that is not consistent with either the uniform thickness, or the surface smoothness of a typical lamella. (4) The tipping of polymer crystals at low draw ratios is not readily understood in terms of bundlelike crystals. (5) It seems improbable that assemblies of strictly bundlelike “lamellae” would cleave along the required planes.

The deficiencies of the bundlelike models, as contrasted with the ability of the chain fold models to reproduce many of the significant structural feaures of lamellar spherulites, leads to the conclusion that it is highly probable that lamellar spherulites formed in bulk consist of structures that are built on a basically chain folded pattern.

To this it must be added that some bundlelike character, in the form of interlamellar links, or chains protruding from the fold plane, must be expected in lamellar spherulites. Further, the existence of a microcrystalline but nonlamellar spherulite built on the bundlelike pattern by noncoherent nucleation is by no means excluded. Therefore, the possiblilty exists that there is more than one basic scheme for the construction of spherulites in bulk despite the evidence that a number that have been carefully studied are lamellar.

Finally, some limitations that may exist on spherulitic growth with chain folds were noted. Exclusion of chain ends from the crystal for reasons of large size may hinder fold formation at low supercooling where the step height is large. At high supercooling, nonsteady state nucleation may occur in the medium surrounding a spherulite, and seriously hinder its growth.

## Figures and Tables

**Figure 10:**
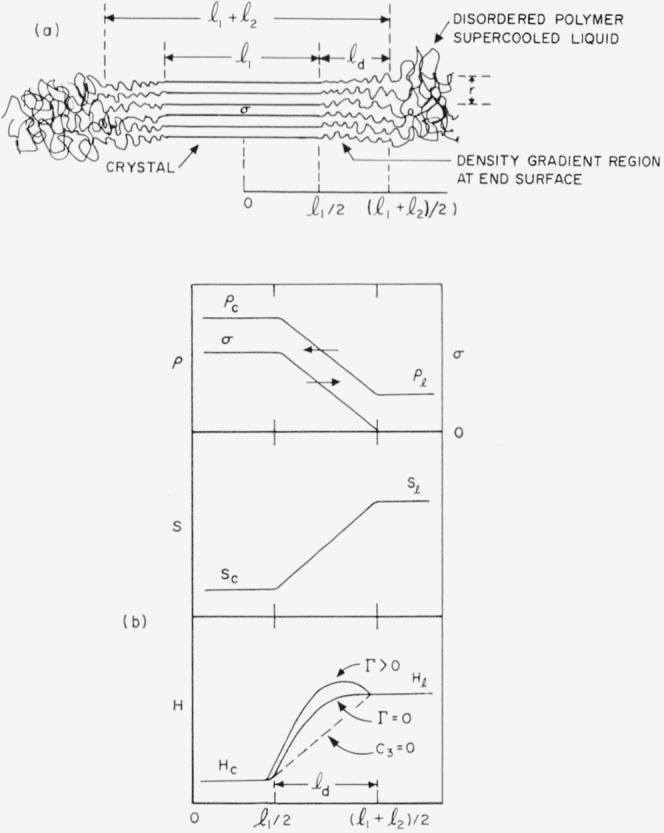
Density gradient model of the primary bundlelike nucleus (a) Schematic diagram of model showing dimensions and coordinates. (b) Density, lateral surface free energy, entropy, and heat content as a function of length in the density gradient region at a bundle end.

**Table 1:** Spherulitic growth

Model	log*_e_*(*G*/*G*_0_)	Temperature range	Rate law	Remarks

Coherent surface nucleus models^a^				

Coherent folded nuclei^b^	$-\frac{\Delta H*}{kT}-\frac{4b_{0}\sigma \sigma_{e}}{(\Delta f)kT}$ (two-dimensional nucleus)	Region **A**′, *T_m_* to *T_c_.*	(Δ*T*)^−1^	Predicts a typical lamellar spherulite.
				
Coherent bundlelike nuclei	$-\frac{\Delta H*}{kT}-\frac{4b_{0}\sigma \sigma_{e}}{(\Delta f)kT}$ (two-dimensional nucleus)	Region A′, *T_m_* to *T_c_*.	(Δ*T*)^−1^	Does not lead to lamellar spherulite. Will give microcrystals scattered throughout medium; formation of spherical object of macroscopic size doubtful.

Noncoherent surface nucleus models^a^				

Noncoherent folded nuclei^b^	$-\frac{\Delta H*}{kT}-\frac{32\sigma (\sigma -\delta /2)\sigma_{e}}{{\left(\Delta f\right)}^{2}kT}$ (three-dimensional nucleus)	Region **A**″ (upper), *T_m_* to $T_{\delta }^{\;\prime \prime }$	(Δ*T*)^−2^	Produces a modified lamellar spherulite. Radius will increase spasmodically in Region **A**″ (upper).
		
	$-\frac{\Delta H*}{kT}-\frac{4b_{0}\sigma \sigma_{e}}{(\Delta f)kT}$ (two-dimensional nucleus)	Region **A**″ (lower), $T_{\delta }^{\;\prime \prime }$ to *T_c_.*	(Δ*T*)^−1^	
				
Noncoherent bundlelike nuclei.	$-\frac{\Delta H*}{kT}-\frac{32\sigma (\sigma -\delta /2)\sigma_{e}}{{\left(\Delta f\right)}^{2}kT}$ (three-dimensional nucleus)	Region **A**″ (upper), *T_m_* to $T_{\delta }^{\;\prime \prime }$.	(Δ*T*)^−2^	May lead to a microcrystalline nonlamellar spherulite.
		
	$-\frac{\Delta H*}{kT}-\frac{4b_{0}\sigma \sigma_{e}}{(\Delta f)kT}$ (two-dimensional nucleus)	Region A″ (lower), $T_{\delta }^{\;\prime \prime }$ to *T_c_.*	(Δ*T*)^−1^	

a ***σ*** and ***σ****_e_* are the lateral and end surface free energies, respectively, for folded nuclei; *σ* and *σ_e_* are the corresponding quantities for bundlelike nuclei. The quantity (Δ*f*) is given by [(Δ*h_f_*) (Δ*T*)/*T_m_*][*T*/*T_m_*].

b In the expressions for log*_e_*(*G*/*G*_0_) for the folded nuclei, the edge free energy ***ϵ*** has been set equal to zero to simplify them (see text for complete expressions).

## Footnotes

1 It is recommended that only the sections marked with a star (★) be considered in a first reading of the paper. These sections treat many of the main issues, and include the most important models of lamellar spherulitic growth (sections 5 and 6).

2 Figures in brackets indicate the literature references at the end of this paper.

3 The schematic representation of the orientation of the lamellae and the polymer molecules in the lamellae, and the general nature of lamellar twist shown in figures 6 and 7 may prove useful in understanding the above general description of a lamellar spherulite. In real spherulites, the lamellae may be more fragmented and imperfect than shown in these figures.

4 For many models treated in this paper, *K* varies as (Δ*T*)^−2^, neglecting less important temperature variations. Even such a dependence on temperature in the pre-exponential term is trivial in the analysis of data, the other terms being much more important.

5 Turnbull finds *σ*=9.6 erg cm^−2^ for *n*-octadecane and *σ*=7.2 erg cm^−2^ for *n*-heptadecane. The high end surface free energy that may exist in large bundlelike nuclei or crystals in a high molecular weight polymer will not appear in such short chain materials.

6 (△*h_f_*) is within a factor of 2 of 1.5×10^9^ erg cm^−3^ for many linear polymers.

7 This reverses an earlier opinion [3] that detailed consideration should be accorded the case where *σ_e_* is substantially smaller than *σ*. Calculations based on the assumption *σ_e_<<σ* lead to a shift in the nucleation rate from the customary (Δ*T*)^−2^ to a (Δ*T*)^−1^ law at some moderate degree of supercooling [3]. It is improbable that this will commonly occur. Equation (3) indicates that it is highly unlikely that *σ_e_* will be zero, as has sometimes been assumed for bundlelike nuclei.

8 The usual expression Δ*f*=Δ*h_f_*(Δ*T*)/*T_m_* is less exact than eq (7) for glass-forming bulk systems. The extra factor *T*/*T_m_* corrects for the fact that the entropy difference between the supercooled liquid and crystal falls below Δ*h_f_/T_m_* as the temperature falls below *T_m_.*

9 Even if the nonsteady state nucleation effect did not interfere at strong supercooling, the (Δ*T*)^+1^ law would not be closely obeyed near *T_c_* because of disturbances in the nucleation rate related to the segmental character of the chain that will occur as *l* approaches *l*_min_.

10 In reference [3], it was shown in eqs (42-48) that the homogeneous nucleation rate for folded nuclei between the length l and l+*d*l is proportional to exp (−Δ*H**/*kT*)exp(−Δ*ϕ**/*kT*)exp $\left\{-(\Delta \phi */kT)\;[{\left(l/l_{p}^{*}-1\right)}^{2}]/[1+2(l/l_{p}^{*}-1)]\right\}$, where Δ*ϕ** is the free energy of formation at the saddle point, which is 32*σ*^2^*σ_e_*/(Δ*f*)^2^ in the present geometry. This expression shows that the nucleation rate is a maximum at $l=l_{p}^{*}$. This was extended to show that primary nuclei with step heights much different than $l_{p}^{*}$ are highly improbable.

11 The derivation of eq (14) is patterned after one given by Mandelkern [24] for a cylindrical bundlelike nucleus.

12 The expressions for *I*_loop_ and *σ*_end loop (tot.)_ do in fact contain a small term in log_e_ *v*_2_, but this is entirely insignificant compared to the ones shown for *I*_bundle_ and *σ*_end bundle (tot.)_ [3].

13 The pre-exponential is sometimes written as *G*_0_*T*, apparently in the belief that the main temperature dependence of the pre-exponential arises from the factor (*kT*/*h*) that applies to the elementary jump rate processes for each individual forward and backward reaction. Actually *I_s_*_(0)_ contains a factor analogous to *K* in eq (1) that varies as (Δ*T*)^−2^ for a number of models. Even this dependence on temperature is negligible in the problems of interest here, and *G*_0_ may be considered to be independent of temperature to a sufficient approximation.

14 Note that ao has a different meaning than *a*_min_. The quantities *a*_0_ and *b*_0_ refer to the appropriate lateral lattice spacings in the polymer crystal, while *a*_min_ and *b*_min_ refer to the minimum values that *a* and *b* may take on in a homogeneous nucleus. In general, *a*_min_ and *b*_min_ wlll be larger than *a*_0_ and *b*_0_.

15 An exception to this occurs in the case of a high energy surface comprised of chain folds, which for obvious reasons cannot grow in the same sense as a bundlelike crystal in the chain direction.

16 It is extremely improbable that chain entanglements would cause all bundlelike nuclei to abruptly stop growing in the *G_l_* direction in such a manner as to produce a uniform length comparable to the thickness of a lamella.

17 The edge free energy is taken as *ϵ* for **l**≤**l***, and zero for **l**>**l***. In a more detailed analysis, the edge free energy might be taken as *ϵ*_1_ for **l**<**l** *, *ϵ*_2_ for **l**=**l** *, and zero for **l**>**l***.

18 The primary nucleus may have a characteristic value of the edge free energy *ϵ_p_*, which will cause $l_{p}^{*}$ to slightly exceed 4*σ_e_*/(Δ*f*) [3], but this may be ignored.

19 If the step height does not increase to any marked extent on storage under isothermal conditions at *T_x_*, and if on rewarming in the manner indicated, a small step height is replaced by a larger one, melting followed by recrystallization is reasonable as the mechanism of the increase **l**.

20 The authors are deeply indebted to Prof. F. C. Frank and Dr. M. P. Tosi for sending them a manuscript [41] of their work on fluctuations of step height prior to publication. The paper of Lauritzen’s referred to above, also unpublished, borrows heavily from the methods outlined by Frank and Tosi. However, they do not treat a polymer chain that is explicitly made up of discrete units. As a result of this and possibly other differences, Frank and Tosi predict that chain folded growth will not occur above a certain temperature because **l***→2*σ_e_*/(Δ*f*). Lauritzen’s calculations indicate that this upper limit is removed by the assumption of discrete chain segments. Both of the treatments with fluctuations suggest that **l***, after falling initially with increasing supercooling more or less according to eq (36), will begin to rise again at strong supercooling. However, Lauritzen’s analysis suggests that this would probably be seen experimentally as a certain flattening out of the **l*** versus *T* curve; the sharp rise in **l*** occurs at such strong supercooling that it will frequently not be in the experimentally accessible range, and may be below *T_c_.* Hence eq (36) applies as a good approximation from moderate to very low supercooling.

21 Other manifestations of increased perfection, such as a sharpening of certain X-ray lines, are also to be expected as the crystallization temperature is increased.

22 The individual chain folded crystals formed from dilute solution may either be flat, or resemble hollow pyramids. In the latter case the folds are staggered, and the chains are not perpendicular to the ***σ****_e_*, plane. (See for example ref. [43].)

23 The author is indebted to J. N. Frankland of NBS for deriving this result, and for several helpful discussions of the problem of twist in lamellar systems.

24 If an edge free energy 2*aϵ* had been included in eq (40)**l*** would have been 4*σ_e_*/Δ*f*+2*ϵ*/(2*σ*−*δ*), and eq (44) would have an additional term exp $\left[-8\sigma \epsilon T_{m}^{2}/T(\Delta h_{f})(\Delta T)kT\right]$. This will not seriously alter the (Δ*T*)^−2^ law predicted by eq (44) in its range of validity.

25 The right hand term in eq (46) will be even smaller than indicated when the growth is strongly arrested by cumulative strain.

26 In making this comment, it is assumed that other possible causes of spasmodic growth, such as fluctuating temperature at the spherulite boundary due to experimental conditions (e.g., rise and fall of bath temperature) or heat dissipation effects, have been eliminated.

27 Even when present in low concentration, noncrystallizable units in a copolymer must be expected to disrupt the formation of regularly folded crystals.

28 It is actually quite difficult to distinguish experimentally between the (Δ*T*)^−1^ and (Δ*T*)^−2^ laws in many cases, largely because of the uncertainty in *T_m_.* The value of Δ*H** must not either implicitly or explicitly be set equal to zero in attempting to determine the Δ*T* law. This strongly and incorrectly favors the (Δ*T*)^−2^ law over the (Δ*T*)^−1^ law. Methods of determining the radial growth rate law from data on spherulites will be discussed in detail in a forthcoming publication [35].

29 Abnormally large Δ*H** values can result for spherulitic growth taking place in a medium that contains competing nuclei or crystallites.

30 In polymers, retained orientation might create memory effects, but it should be possible to eliminate these by storing the samples above *T_m_* for a time.

31 Experiments aimed at differentiating between the “equilibrium” theory of Peterlin and Fischer, where the step height is a function of ambient temperature, and the “kinetic“ theory presented in this paper, where the step height is a function of the growth temperature, must be carried out and interpreted with caution. For example, if a lamella with a certain step height in bulk is warmed, it will melt, and if kinetics permit, recrystallize at a new and larger step height (sec. 5.2). This must not be mistaken for an “equilibrium“ increase in step height. Experiments conducted by lowering the temperature after crystallization are free of this objection provided no additional crystallization of supercooled liquid polymer take place at the lower temperatures, thus introducing thinner lamellae. Studies of the changes of step height of chain folded lamellae in dilute solution are of problematical value in this connection. They may show an increase of step height on warming due to melting followed by recrystallization. If the solubility is finite, thin lamellae will form from thicker ones in dilute solution on lowering the temperature. Neither of these effects in dilute solution may be interpreted as supporting the “equilibrium” theory. In some polymers, a slow increase of step height due to internal diffusion mechanisms in the crystal may take place, and further complicate matters. However, as noted in section 5.2, such a diffusion mechanism is distinguished by the fact it can take place isothermally at the original crystallization temperature.

## 10. Appendix Simplified Density Gradient Model of the Primary Bundlelike Nucleus

### Density Gradient Model

The treatment given below is not represented as a rigorous or complete solution of the problem of the bundlelike nucleus with a density gradient at the bundle ends. Our intention in presenting the treatment below is to illuminate with a simple example some of the factors that might contribute to *σ_e_.*

Consider a bundlelike nucleus with a middle section of normal crystal density *ρ_c_* and lateral surface free energy *σ*, which has length *l*_1_ and radius *r.* In addition, the nucleus has two diffuse bundle ends where the density gradually falls off from *ρ_c_* to *ρ_l_*, the latter being the density of the supercooled liquid. Call the radius of the diffuse end *r*, and let the total length of the nucleus be *l*_1_+*l*_2_. Let the *l* coordinate be zero at the center of the nucleus. Then the normal crystalline section of density *ρ_c_* extends from zero to *l*_1_/2, and the diffuse section, where the density falls to *ρ_l_*, extends from *l*_1_/2 to (*l*_1_+*l*_2_)/2. (See fig. 10a.)

For this model, the free energy of formation may be approximated as

$$\Delta \phi =2\pi rl_{1}\sigma +4\pi rW_{s}+2\pi r^{2}W_{e}-\pi r^{2}l_{1}(\Delta f),$$ (A-1)

where

$$W_{s}=\int_{\frac{1}{2}l_{1}}^{\frac{1}{2}(l_{1}+l_{2})}\sigma (\rho )dl$$ (A-2)

and

$$W_{e}=\int_{\frac{1}{2}l_{1}}^{\frac{1}{2}(l_{1}+l_{2})}\Delta F(\rho )dl.$$ (A-3)

Here *σ*(*ρ*) is the lateral surface free energy as a function of density in the diffuse end region, and Δ*F*(*ρ*) the free energy of formation as a function of density in the same region.

2*πrl*_1_ is the work required to form the lateral surface of the normal crystalline section of length *l*_1_, and 4*πrW_s_* is the work required to form the lateral surface of both diffuse ends. The quantity *σ*(*ρ*) will be equal to *σ* at *l*_1_/2, and zero at (*l*_1_+*l*_2_)/2. *W_s_* will have a positive value, and not depend strongly on temperature.

2*πr*^2^*W_e_* represents the free energy of formation of the two diffuse bundle ends. This will contain some negative contributions, since Δ*F*(*ρ*) is equal to −(Δ*f*) at *l*_1_/2, and zero at (*l*_1_+*l*_2_)/2. However, Δ*F*(*ρ*) will be positive for a certain range of *l* values between these limits, causing the net value of *W_e_* to be positive. *W_e_* may depend on temperature to some extent.

The model does not explicitly treat cumulative strain caused by radial growth. To do so would greatly complicate the model.

By the usual methods one finds *r** = 2*σ/*(Δ*f*) and $l_{1}^{*}=2W_{s}/\sigma +4W_{e}/\Delta f$. On substitution of these in eq (A-1), there is obtained.

$$\Delta \phi *=\frac{8\pi W_{s}\sigma }{\left(\Delta f\right)}+\frac{8\pi \sigma^{2}W_{e}}{{\left(\Delta f\right)}^{2}}.$$ (A-4)

Hence, the homogeneous nucleation rate is,

$$I=I_{0}\;\exp\;\left(-\frac{\Delta H*}{kT}\right)\;\exp\;\left(-\frac{8\pi W_{s}\sigma }{(\Delta f)kT}\right)\;\exp\;\left(-\frac{8\pi \sigma^{2}W_{e}}{{\left(\Delta f\right)}^{2}kT}\right).$$ (A-5)

The (Δ*T*)^−1^ term involving *W_s_* will in some cases be nearly cancelled by components of opposite sign arising from *W_e_* (see below). In any event, eq (A-5) will lead to a (Δ*T*)^−2^ nucleation rate behavior sufficiently near *T_m_.*

### Comparison With Sharp Boundary Model

The corresponding cylindrical bundlelike model with *σ_e_* treated as a single constant representing the surface free energy as if it were concentrated at an abrupt phase boundary has the free energy of formation

$$\Delta \phi =2\pi rl_{1}\sigma +2\pi r^{2}\sigma_{e}-\pi r^{2}l_{1}(\Delta f),$$ (A-6)

which leads to

$$\Delta \phi *=\frac{8\pi \sigma^{2}\sigma_{e}}{{\left(\Delta f\right)}^{2}}$$ (A-7)

and

$$I=I_{0}\;\exp\;\left(-\frac{\Delta H*}{kT}\right)\;\exp\;\left(-\frac{8\pi \sigma^{2}\sigma_{e}}{{\left(\Delta f\right)}^{2}}\right).$$ (A-8)

Except for unimportant differences in geometry, eqs (A-6) and (A-8) are analogous to eqs (4) and (6) or (18) of the text.

Comparison of eqs (A-4) and (A-7) show that

$$\sigma_{e}=W_{e}+\frac{(\Delta f)W_{s}}{\sigma }.$$ (A-9)

A similar result is obtained if the parallelepiped geometry is used to treat the bundlelike density gradient model. Therefore, to the approximations inherent in eq (A-1), the above expression may be regarded as showing some of the factors that contribute to the effective value of *σ_e_* for the bundlelike nucleus treated as if it had a sharp phase boundary at the bundle ends.

### Application

A highly approximate but nevertheless instructive application of the density gradient model will now be given. The objective is to calculate *σ_e_* as given by eq (A-9) in terms of the appropriate quantities. This requires that *W_s_* and *W_e_* be evaluated.

In order to calculate *W_e_*, some assumptions must be made concerning Δ*F*(*ρ*). A crude model will suffice for the purpose of illustrating what contributes to *W_e_*,

Begin by assuming that, at constant temperature, the entropy as a function of density in the diffuse bundle end has the form *C*_1_(*ρ*_0_/*ρ*), where *C*_1_ is a positive constant in the units erg deg^−1^ cm^−3^, *ρ*_0_ a reference density (*ρ_l_*+*ρ_c_*)/2, and *ρ* the density characteristic of some value *l* in the bundle end. No claim is made that this is exactly the relationship between entropy and density. However, the proposed expression does lead to an increase in entropy with a decrease in density, corresponding to the increase of entropy that must occur as a path is traversed from *ρ* = *ρ_c_* at *l*_1_/2 at the normal crystal, out through the increasingly disordered bundle end to *ρ* = *ρ_l_* at (*l*_1_+*l*_2_)/2. From the proposed empirical function we see that

$$S-S_{l}=\Delta S(\rho )=C_{1}\rho_{0}\left(\frac{1}{\rho }-\frac{1}{\rho_{l}}\right).$$ (A-10)

Assume further that the heat content as a function of density in the bundle ends is of the form *C*2(*ρ*/*ρ*_0_) + *C*_3_(*ρ*_0_/*ρ*). Then

$$H-H_{l}=\Delta H(\rho )=\frac{C_{2}(\rho_{l}-\rho )}{\rho_{0}}+C_{3}\rho_{0}\left(\frac{1}{\rho_{l}}-\frac{1}{\rho }\right).$$ (A-11)

The constants *C*_2_ and *C*_3_ are in erg cm^−3^. This function is proposed with the express intent of causing a maximum to exist in Δ*H*(*ρ*) as one goes from *l*_1_/2, where *ρ* = *ρ_c_* and *H* = *H_c_*, out through the bundle end to (*l*_1_+*l*_2_)/2, where *ρ* = *ρ_l_* and *H*=*H_l_.* (Both *C*_2_ and *C*_3_ must have positive values to give this maximum.) This maximum, whose magnitude is related to *C*_3_ and Γ (see below), may be considered to result from noncumulative volume strain (repulsions or abnormal separations, of the chain segments) in the bundle end. The maximum in Δ*H*(*ρ*) in turn contributes to a maximum in Δ*F*(*ρ*) whose height depends on *C*_3_ (or Γ). *This maximum in the free energy must exist in the surface region in order to cause phase separation.* (There is a small maximum in Δ*F*(*ρ*) even if *C*_3_=0.) No assertion is made that eq (A-11) is an accurate representation of the heat content as a function of density in the bundle end; it is merely an empirical function meeting certain boundary conditions and other physical requirements of the problem at hand.

Accordingly, the free energy *F*−*F_l_* in the boundary region is

$$F-F_{l}=\Delta F(\rho )=\frac{C_{2}(\rho_{l}-\rho )}{\rho_{0}}+C_{3}\rho_{0}\left(\frac{1}{\rho_{l}}-\frac{1}{\rho }\right)-TC_{1}\rho_{0}\left(\frac{1}{\rho }-\frac{1}{\rho_{l}}\right).$$ (A-12)

Applying the conditions *F_l_*−*F_c_* = Δ*h_f_*(*T_m_*−*T*)/*T_m_*, and *F_l_*−*F_c_*= 0 at *T_m_*, one finds *C*_1_ = (Δ*h_f_*/*ρ*_0_*T_m_*) (*ρ_c_ρ_l_*/Δ*ρ_l_*) and $C_{2}=\rho_{0}^{2}\left(C_{3}+T_{m}C_{1}\right)/\rho_{c}\rho_{l}$, where Δ*ρ* = *ρ_c_* − *ρ_l_*. This gives

$$F-F_{l}=\rho_{0}C_{3}\left[\frac{1}{\rho_{c}}+\frac{1}{\rho_{l}}-\frac{\rho }{\rho_{c}\rho_{l}}-\frac{1}{\rho }\right]+\frac{\Delta h_{f}\rho_{c}\rho_{l}}{T_{m}(\Delta \rho )}\left[\frac{T_{m}}{\rho_{c}}+\frac{T}{\rho_{l}}-\frac{T}{\rho }-\frac{T_{m}\rho }{\rho_{c}\rho_{l}}\right].$$ (A-13)

The expression for *W_e_* may be written

$$W_{e}=\int_{\rho_{c}}^{\rho_{l}}(F-F_{l})\frac{dl}{d\rho }d\rho .$$ (A-14)

Assuming that the density falls off linearly with *l* in the density gradient region, *dl/dρ*= −*l_d_*/(Δ*ρ*), where *l_d_* is the length of the gradient region at one of the bundle ends. Carrying out the integration and expanding ln(*ρ*_c_/*ρ_l_*) as $\left(\Delta \rho /\rho_{l}\right)-\frac{1}{2}{\left(\Delta \rho /\rho_{l}\right)}^{2}+\frac{1}{3}{\left(\Delta \rho /\rho_{l}\right)}^{3}-\ldots ,$ the result is

$$W_{e}=\frac{l_{d}{\left(\Delta \rho \right)}^{2}}{6\rho_{l}^{2}\rho_{c}}\left[\rho_{0}C_{3}+\frac{\Delta h_{f}\rho_{l}\rho_{c}}{\left(\Delta \rho \right)}\right]-\frac{l_{d}(\rho_{c}/\rho_{l})(\Delta f)}{2}.$$ (A-15)

Both of the terms in the brackets make positive contributions to *W_e_*, since *C*_3_ is positive. In arriving at (A-15) it was assumed that terms involving (Δ*ρ*/*ρ*)^4^ could be neglected.

The behavior of *W_s_* may now be examined. Assuming that *σ*(*ρ*) falls off linearly from *σ* at *ρ_c_* to zero at *σ_l_* as

$$\sigma (\rho )=\sigma \left(\frac{\rho -\rho_{l}}{\Delta \rho }\right),$$ (A-16)

one gets

$$W_{s}=\frac{l_{d}\sigma }{2}.$$ (A-17)

Combining (A-15) and (A-17) according to (A-9) we get

$$\sigma_{e}=\frac{l_{d}{\left(\Delta \rho \right)}^{2}}{6\rho_{l}^{2}\rho_{c}}\left[\rho_{0}C_{3}+\frac{(\Delta h_{f})\rho_{l}\rho_{c}}{\left(\Delta \rho \right)}\right].$$ (A-18)

A term [*l_d_*(Δ*f*)/2](1 − *ρ_c_*/*ρ_l_*) has been omitted from eq (A-18) since it is too small to be of any consequence in this case. In other instances, however, the term involving (Δ*f*) could be larger.

The nature of the various thermodynamic functions in the end surface region are shown schematically in figure 10b as a function of the distance traversed out through the bundle end. These were converted from *σ*(*ρ*), Δ*H*(*ρ*) and Δ*S*(*ρ*) by assuming *ρ* is a linear function of *l.*

For *C*_3_=0, which corresponds to a mono tonic increase of heat content in the bundle ends, it is seen that *σ_e_=l_d_*(Δ*ρ*)(Δ*ρ*)/6*ρ_l_.* This comes to roughly 9 erg cm^−2^ for the particular example relating to polyethylene cited in section 2.2. However, it is clear on physical grounds that the heat content must possess at least a flat maximum in the boundary region corresponding to a nonzero value of *C*_3_. Consideration of this leads to a larger and more realistic minimum value of *σ_e_.*

In a system where each molecule is forced to participate in the crystal and liquid (or supercooled liquid) phases, as is the case at the end of a bundlelike nucleus or crystal in a polymer, we consider it highly probable that the heat content at some point in the surface phase must be even higher than it is in the liquid. This effect may be taken to be a result of the volume strain that must occur in such situations. (The above remarks refer to nuclei of substantial size, say with a radius of 50 A or more, where density differences between the liquid and crystal will become effective. The volume strain effect would tend to be unimportant if only three or four chains were involved. The model is in any case not valid for such small radii.)

Consider now the value of *C*_3_ that will lead to a maximum in Δ*H*(*ρ*) between *l*_1_/2 and (*l*_1_+*l*_2_)/2. It is easily shown that *C*_3_ must be larger than (Δ*h_f_*) $\rho_{l}^{2}\rho_{c}/\rho_{0}{\left(\Delta \rho \right)}^{2}$ in order that this maximum exist.^32^ Therefore we may write

$$C_{3}=\frac{(\Delta h_{f})\rho_{l}^{2}\rho_{c}}{\rho_{0}{\left(\Delta \rho \right)}^{2}}+\Gamma$$ (A-19)

where Γ ≧ 0. Hence, from (A-18) and (A-19), one gets

$$\sigma_{e}=\frac{l_{d}(\Delta h_{f})\rho_{c}}{6\rho_{l}}+\frac{l_{d}{\left(\Delta \rho \right)}^{2}\rho_{0}\Gamma }{6\rho_{l}^{2}\rho_{c}}.$$ (A-20)

The quantity Γ is in erg cm^−3^. The case Γ=0 corresponds to that where there is no maximum in Δ*H*(*ρ*). This in turn corresponds to the smallest maximum in Δ*F*(*ρ*), and therefore the smallest value of *σ_e_*, that is physically realistic according to the model. Actual values of *σ_e_* would almost certainly involve Γ>0.

The particular model treated above does not lead to a large temperature dependence of *σ_e_*, though the term (Δ*ρ*)^2^ will fall with lowering temperature. However, there are a number of reasons for expecting *σ_e_* to depend somewhat more on temperature in the more general case. For example, the residual term involving (Δ*f*) that practically cancelled in eq (A-18) may be larger if other assumptions concerning Δ*F*(*ρ*)*, σ*(*ρ*), and *dl/dρ* are used.
